# Solid-Phase Synthesis and Circular Dichroism Study of β-ABpeptoids

**DOI:** 10.3390/molecules24010178

**Published:** 2019-01-05

**Authors:** Ganesh A. Sable, Kang Ju Lee, Hyun-Suk Lim

**Affiliations:** Department of Chemistry and Division of Advanced Material Science, Pohang University of Science and Technology (POSTECH), Pohang 37673, Korea; sganesh105@postech.ac.kr (G.A.S.); dlrkdwn2tlfd@postech.ac.kr (K.J.L.)

**Keywords:** solid-phase synthesis, peptidomimetics, foldamers, peptoids, β-ABpeptoids

## Abstract

The development of peptidomimetic foldamers that can form well-defined folded structures is highly desirable yet challenging. We previously reported on α-ABpeptoids, oligomers of *N*-alkylated β^2^-homoalanines and found that due to the presence of chiral methyl groups at α-positions, α-ABpeptoids were shown to adopt folding conformations. Here, we report β-ABpeptoids having chiral methyl group at β-positions rather than α-positions as a different class of peptoids with backbone chirality. We developed a facile solid-phase synthetic route that enables the synthesis of β-ABpeptoid oligomers ranging from 2-mer to 8-mer in excellent yields. These oligomers were shown to adopt ordered folding conformations based on circular dichroism (CD) and NMR studies. Overall, these results suggest that β-ABpeptoids represent a novel class of peptidomimetic foldamers that will find a wide range of applications in biomedical and material sciences.

## 1. Introduction

Peptidomimetic foldamers are a class of artificial oligomers that can form defined and predictable structures that are capable of mimicking the three-dimensional structure and function of natural peptides and proteins [1,2]. Given their improved proteolytic stability and structural diversity compared to native peptides, synthetic peptidomimetic foldamers that adopt well-defined protein secondary structures can be highly useful in biological applications and material sciences [3]. As such, the development of such synthetic foldamers is of great interest and a number of different classes of peptidomimetic foldamers have been described over the last two decades, including β- and γ-peptides [4,5,6,7,8], oligoureas [9,10], oligopyrrolidines [11], γ-AApeptides [12,13], oligotriazoles [14], azapeptides [15,16], oligocarbamates [17], α-aminoxy acid containing oligomers [18], and peptoids [19,20,21].

**Figure 1:**
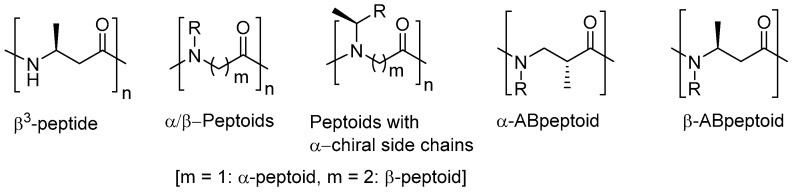
General structures of β^3^-peptides, α- and β-peptoids, α- and β-peptoids with α-chiral side chains, α-ABpeptoids and β-ABpeptoids.

Peptoids are oligomers of *N*-alkylated glycines [22]. They are efficiently synthesized by submonomer solid-phase synthesis [23]. Like other unnatural peptidomimetic foldamers, peptoids are resistant to proteolysis [24,25]. While native peptides and most peptidomimetics are generally unable to penetrate cell membranes, presumably due to the presence of amide protons, peptoids are relatively cell permeable [26,27,28]. Although peptoids possess many of the desirable features of peptidomimetics, they are structurally flexible and generally do not form folding structures because of the lack of amide protons and chirality in their backbones. To overcome the intrinsic limitation of peptoids associated with their flexible nature, various strategies have been developed. For instance, the development of macrocyclic peptoids has been of significant interest to restrict the conformational flexibility of linear peptoids [29,30,31,32,33,34,35]. Alternatively, it has been proven that the introduction of chiral centers to peptoid frameworks enables them to have ordered folding structures, which is in contrast to regular peptoids lacking chirality. For example, peptoid oligomers substituted with α-chiral side chains are found to adopt well-defined folding conformations like polyproline-type helical structures (Figure 1) [36,37,38,39,40,41]. 

Recently, our group reported α-ABpeptoids (α-alkyl beta-peptoids) as a new class of peptoids [42,43]. They are oligomers of *N*-alkylated β^2^-homoalanines and structurally similar to β-peptoids, except that they have chiral methyl groups at α-positions (Figure 1). As a result, and in contrast to general peptoids, α-ABpeptoids contain backbone chirality. α-ABpeptoid oligomers were shown to adopt folding conformations based on circular dichroism (CD) and NMR studies. This finding demonstrates that chiral methyl groups in the peptoid backbone act like those in peptoid with α-chiral side chains. Here we report the design, solid-phase synthesis, and preliminary structural studies of β-ABpeptoids having chiral methyl group at β-positions rather than α-positions as a different class of peptoids with backbone chirality.

## 2. Results and Discussion

### 2.1. Synthesis of Monomer Building Blocks

**Scheme 1:**
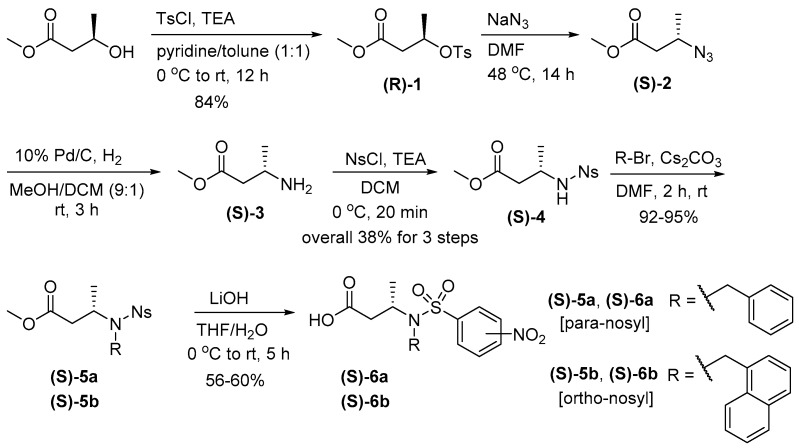
Synthesis of *N*-Alkyl *β*^3^-Homoalanine **(S)-6** building block monomers (rt = room temperature).

As demonstrated in α-ABpeptoids, we envisioned that the introduction of chiral methyl groups at the backbone β-positions would also influence the structural conformation of peptoids. To explore this, we synthesized a series of β-ABpeptoid oligomers using a facile solid-phase synthetic method. Initially, we prepared β^3^-homoalanines substituted with benzyl or naphthyl group as monomer building blocks because it is known that aromatic side chains such as *N*-benzyl and *N*-naphthyl groups in peptoids with chiral side chains play a critical role in forming ordered structures (Scheme 1) [41,42,43,44,45]. (*R*)-methyl 3-hydroxy-3-methylpropanoate, a commercially available and inexpensive starting material, was tosylated to give **(R)-1** in 84% yield. The tosyl group of **(R)-1** was then replaced with the azide group by treatment with sodium azide to produce azide **(S)-2** with an inversion of configuration. The resulting azide **(S)-2** was reduced to obtain **(S)-3**, which was then nosylated to afford **(S)-4**. *N*-Alkylation was performed by treatment with benzyl bromide or naphthyl bromide in the presence of cesium carbonate and *N*,*N*-dimethylformamide (DMF) at room temperature (rt), providing **(S)-5** in 92–95% yields. Finally, hydrolysis with lithium hydroxide gave *N*-alkylated nosyl-protected **(S)-6**. *N*-Benzylated monomer with opposite chirality **(R)-6a** was also prepared using the same procedure starting with (*S*)-methyl 3-hydroxy-3-methylpropanoate (Scheme 1). This synthetic route was highly efficient, yielding the desired monomer building blocks in enantiomerically pure forms without significant racemization as determined by comparing their optical rotation (Table S1). In addition, the enantiomeric purity was further confirmed by using chiral HPLC (Supplementary Information Figure S1). The enantiomeric excess (ee) for **(R)-6** and **(S)-6** was 94% and 85%, respectively, showing the synthetic efficiency.

### 2.2. Solid-Phase Synthesis of β-ABpeptoid Oligomers

**Scheme 2:**
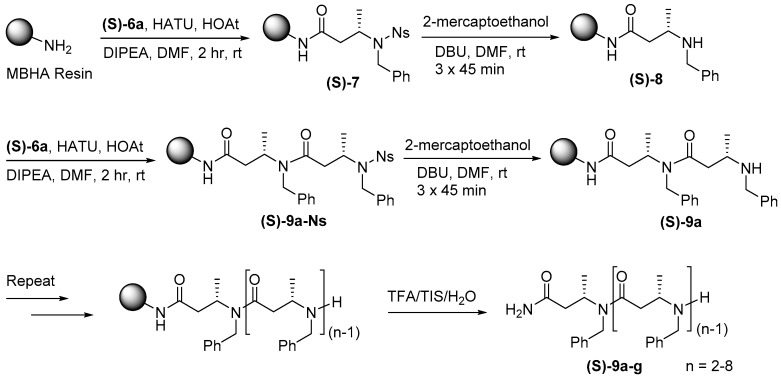
Solid-phase synthesis of oligomers of *N*-Alkyl β^3^-Homoalanine.

**Figure 2:**
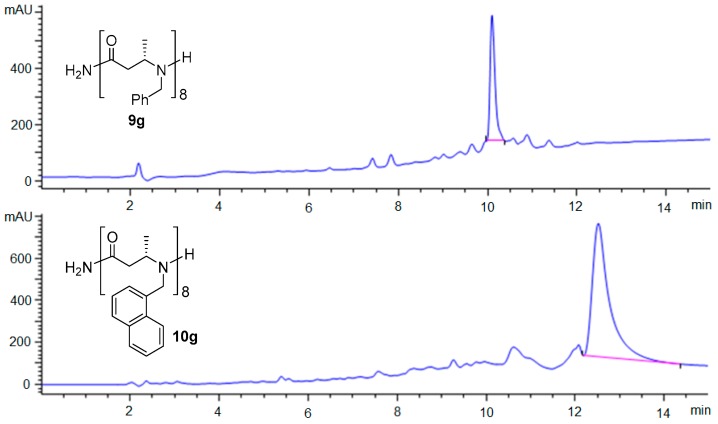
HPLC chromatograms of crude β-ABpeptoid oligomers **9g** and **10g**.

For solid-phase synthesis of *N*-benzyl substituted β-ABpeptoid oligomers, **(S)-6a** was first loaded on Rink amide MBHA resin in the presence of hexafluorophosphate azabenzotriazole tetramethyl uronium (HATU)/1-hydroxy-7-azabenzotriazole (HOAt)/*N*,*N*-diisopropylethylamine (DIPEA) in DMF at rt for 2 hr (Scheme 2). The nosyl group of **(S)-7** was then deprotected by treating it with 2-mercaptoethanol and 1,8-diazabicycloundec-7-ene in DMF, giving resin bound secondary amine **(S)-8**. In our previous method to synthesize α-ABpeptoid oligomers, we used *N,N*′-diisopropylcarbodiimide (DIC) as a coupling reagent. In the present work, we initially employed the same DIC condition, but the reaction was not successful. After testing several different coupling conditions, we found that the use of HATU/HOAt/DIPEA [46,47] provided the desired dimer **(S)-9a-Ns** with excellent yield (Supplementary Information Figure S2). Iterative deprotection and coupling reactions allowed for the generation of *N*-benzylated β-ABpeptoid oligomers ranging from 2- to 8-mers **(S)-9a**–**g** (Table 1 and Supplementary Information Figure S3). After the completion of solid phase synthesis, the resulting β-ABpeptoids were cleaved from resin by using cleavage cocktail containing 95% trifluoroacetic acid (TFA), 2.5% water, and 2.5% triisopropylsilane (TIPS). The resulting crude products were purified by HPLC, and their identity was determined by mass spectrometry (Table 1 and Supplementary Information Figure S4, Table S2–S4). To examine the efficiency of our solid-phase method, we synthesized β-ABpeptoid oligomers substituted with *N*-naphthyl groups **(S)-10a**–**g** using *N*-napthylated monomer **(S)-6b**. Using the same solid-phase synthetic route, *N*-naphthylated β-ABpeptoids ranging from 2- to 8-mers **(S)-10a**–**g** were prepared in high yield (Table 1 and Supplementary Information Table S2). As shown in Figure 2, the crude purity of the 8-mer oligomers substituted with benzyl or naphthyl group was excellent, demonstrating the robustness of our solid-phase method. Although the synthetic method developed by Sando and co-workers is elegant and convenient, the synthetic route was not sufficiently efficient for generating longer oligomers (20–30% for pentamers) [48]. On the contrary, our solid-phase method is able to synthesize longer β-ABpeptoid oligomers with remarkable efficiency (71–81% for 8-mer β-ABpeptoids as determined by analytical HPLC of the crude products, Figure 2 and Table 1). 

### 2.3. Structural Studies

**Figure 3:**
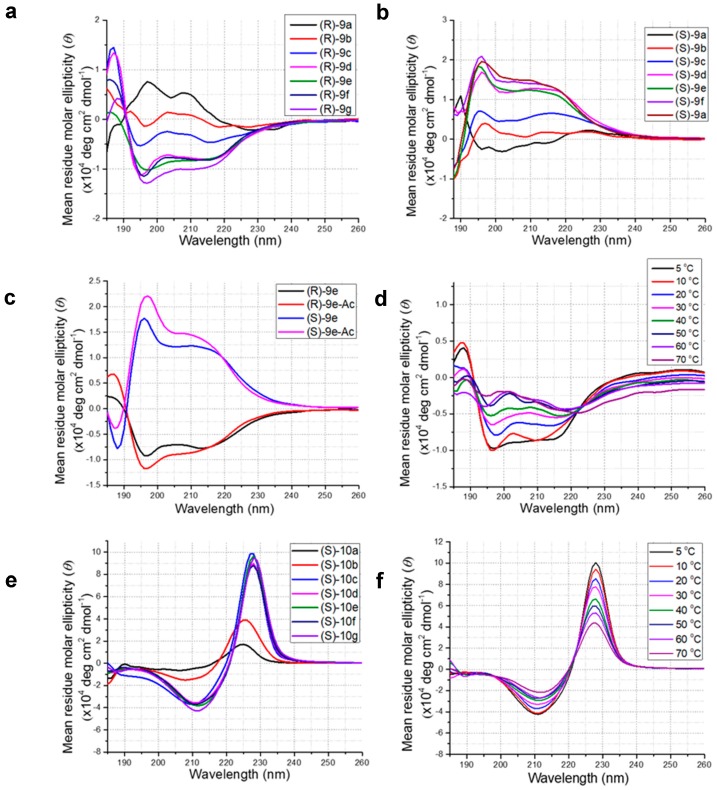
CD data; (**a**) CD spectra of (*R*)-form of *N*-benzylated β-ABpeptoid oligomers (**R**)**-9a**–**g** in acetonitrile (60 μM), (**b**) CD spectra of (*S*)-form of *N*-benzylated β-ABpeptoid oligomers (**S**)**-9a**–**g** in acetonitrile (60 μM), (**c**) CD spectra of (*S*)- and (*R*)-forms of *N*-benzylated β-ABpeptoid hexamers with and without *N*-acetyl in acetonitrile (60 μM). (**d**) CD spectra of (**R)-9e** at 5−70 °C in acetonitrile (60 μM), (**e**) CD spectra of (*S*)-form of *N*-napthylmethyl β-ABpeptoid oligomers (**S**)**-10a**–**g** in acetonitrile (60 μM), (**f**) CD spectra of (*S*)-form of *N*-napthylmethyl β-ABpeptoid (**S**)**-10g** at 5–70 °C in acetonitrile (60 μM).

Next, we measured circular dichroism (CD) spectra to explore the folding propensity of the synthesized β-ABpeptoid oligomers. The CD spectra of (R) form of *N*-benzylated β-ABpeptoids **(R)-9a**–**g** were first recorded in acetonitrile (ACN) at 20 °C (Figure 3a). While dimer **(R)-9a** did not display an apparent CD signal, trimer **(R)-9b** showed a distinct CD signature. The CD intensity was gradually increased as the residues were added, and oligomers with more than four residues exhibited nearly identical CD spectra with an intense minimum at near 196 nm and a shallow minimum near 218 nm. Notably, the CD spectra of β-ABpeptoid oligomers are quite different from those of α-ABpeptoids [42,43]. Their spectral shapes resemble the previously reported CD spectra for β-peptoids [49] and α-peptoids [37] bearing α-chiral, aromatic side chains, suggesting that *N*-benzylated β-ABpeptoid oligomers might adopt a similarly ordered structure. This result was further supported by the nuclear Overhauser effect (NOE) experiments using short oligomers **(R)-9a**–**c** (Supplementary Information Figure S10)_._ We found that the number of NOEs for trimer **(R)-9b** and tetramer **(R)-9c** was markedly increased compared to that of dimer **(R)-9a**. Because these additional NOEs are normally observed in ordered structures and unfolded structures do not produce such NOEs, this observation, in agreement with the CD results, demonstrates that β-ABpeptoids with more than three residues can adopt ordered structures. However, we cannot rule out the possibility that the increased NOE signals could result from the presence of multiple conformational states, and further structural studies are necessary.

We then investigated the CD spectra of *N*-benzylated β-ABpeptoid oligomers with opposite chirality **(S)-9a**–**g** (Figure 3b). (S)-Form oligomers ranging in size from 2-mer to 8-mer were synthesized using *N*-benzylated monomer with opposite chirality **(R)-6a** through the same solid-phase synthetic method (Scheme 2). As anticipated, (*S*)-form β-ABpeptoid oligomers revealed mirror image CD spectra compared to (R)-form oligomers (Figure 3b), demonstrating that β-ABpeptoids possessing opposite backbone chirality adopt identical structures consisting of opposite handedness. These results are in good agreement with the findings by Wu et. al. where they showed that the peptoid with aromatic, α-chiral (*S* or *R*) side chains show mirror image helical CD spectra [37]. This also indicates that the ordered conformation of β-ABpeptoid oligomers is due to the presence of chiral methyl groups on their backbone. We then tested whether the *N-*terminus amine could affect the conformation of these oligomers through terminal intramolecular hydrogen bonding. We synthesized (*R*)- and (*S*)-forms of *N-*acylated hexamers **(S)-9e-Ac** and **(R)-9e-Ac** by reacting with acetic anhydride/DIPEA/DMF (Supplementary Information Table S2 and S3). The CD spectra of the acetylated hexamers **(S)-9e-Ac** and **(R)-9e-Ac** were almost identical in shape and intensity to those of **(S)-9e** and **(R)-9e** (Figure 3c), showing that *N-*terminal amine had no effect on the formation of the ordered structure.

To assess the solvent effects on the conformations of oligomers, CD spectra were recorded of oligomers of **(S)-9a**–**g** in various polar solvents such as phosphate-buffered saline (PBS) buffer-ACN (1:3), MeOH, and trifluoroethanol (TFE) (Supplementary Information Figures S5 and S6). The oligomers maintain similar CD spectral shapes in PBS buffer-ACN (1:3) solvents, however, in MeOH and TFE solvents, the peaks are intensified and the overall spectral shapes are broadened, suggesting that the formation of conformation could be a result of steric interactions between side chains that may alter in a solvent dependent manner, as demonstrated in *N*-benzylated peptoids with α-chiral side chains [39,50]. Next, we investigated the temperature dependence of the ordered structure. The CD spectrum of hexamer **(R)-9e** was measured at different fixed temperatures ranging from 5 to 70 °C (Figure 3d). We found that signal intensity was gradually decreased upon heating, indicating the temperature-dependent denaturation of the folded structure. The thermal denaturation behavior was similarly observed for previously reported β-peptoids, which had stable helical conformations [41].

In addition to *N*-benzylated β-ABpeptoids, we also obtained the CD spectra of *N*-naphthylated β-ABpeptoid oligomers **(S)-10a-g** at 20 °C in acetonitrile (Figure 3e). The spectrum of dimer **(S)-10a** displayed no apparent signal, while trimer **(S)-10b** exhibited characteristic spectral features with a minimum at near 212 nm and an intense maximum at near 228 nm. For tetramer **(S)-10c** and longer oligomers **(S)-10d**–**g**, CD intensities were significantly increased and nearly identical. This result indicates that tetramer or longer oligomers of *N*-napthyl-β^3^-homoalanine form oligomeric length dependent stable ordered structures. Interestingly, the spectral shape differs considerably from those of *N*-benzylated β-ABpeptoids and is somewhat similar to the CD spectra of β-peptoids bearing a similar α-chiral napthyl ethyl side chain [41]. As demonstrated in the previous study by Laursen et al. [41], the notable differences in the CD shapes between the *N*-benzylated oligomers **(S)-9a**–**g** and *N*-naphthylated ones **(S)-10a**–**g** could be attributed to the different side chains, rather than to the formation of different secondary structures. Further studies are needed to determine their three-dimensional structures. 

Next, we examined the thermal stability of the ordered structure of *N-*napthyl β-ABpeptoid. The CD spectrum of octamer **(S)-10g** was recorded at various temperatures ranging from 5 to 70 °C (Figure 3f). The intensity of both peaks at 212 nm and 228 nm was gradually decreased upon heating, but the CD spectra returned to the original shape when the temperature decreased to 20 °C (Figure S8). This finding showed that the observed temperature-dependent changes in the CD spectra was due to the thermal denaturation of the folded structure on heating, as observed in *N*-naphthylated β-peptoids with chiral side chains [37,41]. We then examined the solvent effect by recording the CD spectra of oligomers of **(S)-10a**–**g** in polar protic solvents such as PBS-ACN (1:3), MeOH and trifluoroethanol (TFE) (Supplementary Information Figure S7). Like *N-*benzyl substituted oligomers, *N*-naphthylated β-ABpeptoid oligomers exhibited nearly identical CD spectral shapes in polar protic solvents compared to those observed in acetonitrile. Overall, CD spectroscopic data for β-ABpeptoid oligomers indicate that they form ordered three-dimensional structures, while further structural studies such as high-resolution X-ray studies are needed to fully ascertain their structures. 

## 3. Materials and Methods

### 3.1. General

All chemicals and reagents were purchased from commercial suppliers (Sigma-Aldrich, Saint Louis, MO, USA; TCI, Tokyo, Japan) and used without further purification, unless specified. In particular, chiral starting materials (methyl (*R*)-3-hydroxybutanoate and methyl (*S*)-3-hydroxybutanoate) were purchased from Alfa Aesar (Haverhill, MA, USA). Rink amide MBHA resin (0.45 mmol/g) was purchased from BeadTech (Seoul, South Korea). Analytical HPLC and LC/MS were performed on Agilent systems (Santa Clara, CA, USA) with a C18 reversed phase HPLC column (Eclipse XDB, 3.5 μm, 4.6 mm × 150 mm). Linear gradients of A/B solvents (solvent A: 100% H_2_O, 0.1% TFA; B: 100% acetonitrile, 0.1% TFA) were used with the flow rate of 0.7 mL/min (10–60 to 100% B gradient over 7 min followed by 100% B until 13–15 min). Preparative HPLC purification was performed on an Agilent 1120 Compact LC system (Agilent Technology) with a C18 reversed-phase column (Agilent Technology, 5 μm, 21.2 mm × 150 mm) using a linear gradient from 20% B to 90% B by changing solvent composition over 50 min with the flow rate of 5 mL/min. Chiral HPLC was performed on AQUITY UPLC system Waters with CHIRALPAK IG 5μm (4.6 × 250 mm ID) column and UV detection at 230 nm. For analysis 0.5 µL sample (1 mg/mL) was injected with the flow rate of 1.0 mL/min of isocratic mobile phase (*n*-hexane/EtOH/DEA 80:20:0.1 or *n*-hexane/EtOH/TFA 80:20:0.1) over 25 to 30 min. CD spectra were recorded on a Jasco J-815 spectropolarimeter (Jasco Corporation, Tokyo, Japan). The CD spectra were measured in the quartz cuvette with a 2 mm path length. The spectra were averages of 5 successive accumulations, employing 100 nm/min scan rate. Data were expressed in terms of per-residue molar ellipticity (deg cm^2^/dmol), as calculated per mole of amide groups present and normalized by the molar concentration of peptoids. Smoothing and correction of the background spectra was performed afterwards. ^1^H- and ^13^C-NMR spectra were collected at resonance frequencies of 300, 500 or 600 MHz and 125.7 MHz, respectively using Bruker AVANCE 600 spectrometer (Bruker, Billerica, MA, USA). Proton 2D NOESY experiments were carried out on a Bruker AVANCE 600 spectrometer equipped with a xyz gradient triple-resonance probe. The samples were dissolved in CD_3_CN and the NOESY spectra were recorded with a mixing time of 300 ms.

### 3.2. Chemistry

#### 3.2.1. Solution-Phase Synthesis Building Block Monomers and Intermediates

*Methyl (R)-3-(tosyloxy)butanoate* [**(R)-1**]. A solution of *p-*toluenesulfonyl chloride (35.1 g, 184 mmol, 1.15 equiv) in toluene (50 mL) was added in a dropwise manner to a stirred solution of methyl (*R*)-3-hydroxybutanoate (20 g, 160 mmol) in anhydrous pyridine (50 mL) under N_2_. The reaction mixture was stirred overnight after warming to room temperature and quenched with 1N aqueous HCl (100 mL). The resulting mixture was then extracted with EtOAc (3 × 200 mL) and the combined organic layers were washed with brine (100 mL), dried over Na_2_SO_4_, filtered and concentrated in vacuo. The crude residue was purified by SiO_2_ flash chromatography to give the title compound **(R)-1** (38.7 g, 84%) as a white solid. [α]20D −8.3 (c 3.0, CH_3_CN); ^1^H-NMR (500 MHz, CDCl_3_): δ 7.79 (d, *J* = 8 Hz, 2H), 7.35 (d, *J* = 8 Hz, 2H), 4.97 (sextet, *J* = 6.5 Hz, 1H), 3.6 (s, 1H), 2.73 (dd, *J* = 6.5 and 15.5 Hz, 1H), 2.52 (dd, *J* = 6.5 and 16 Hz, 1H), 2.45 (s, 3H), 1.35 (d, *J* = 6.5 Hz, 3H). ^13^C-NMR (500 MHz, CDCl_3_): δ 179.69, 144.71, 134.01, 129.77 (×2), 127.78 (×2), 75.74, 51.81, 41.29, 21.60, 20.94. Mass *m*/*z* calcd. for [M + H]^+^ 273.8, found [M + H]^+^ 273.8.

*Methyl (S)-3-((4-nitrophenyl)sulfonamido)butanoate* [**(S)-4**]. NaN_3_ (5.26 g, 80.8 mmol, 1.1 equiv) was added to a mixture of **(R)-1** (20 g, 73.5 mmol) in DMF (70 mL). The mixture was stirred at 48 °C for 14 h. The mixture was cooled down to room temperature and filtered to remove insoluble solid. The filtrate was diluted with water (150 mL) and the product was extracted with 5% EtOAc in diethyl ether (2 × 200 mL). The organics were washed with brine (200 mL), dried over Na_2_SO_4_ and concentrated under reduced pressure to afford the crude intermediate methyl (*S*)-3-azidobutanoate [**(S)-2**], 8.2 g], which was used for the next step without further purification (caution: product is volatile). The crude material **(S)-2** (8.2 g) was dissolved in MeOH/CH_2_Cl_2_ (9:1, 120 mL) and then hydrogenated with catalytic Pd/C 10% (0.82 g) at 30 psi for 4 h. The catalyst was removed by filtration through Celite^®^, and the filtrates were evaporated to afford crude methyl (*S*)-3-aminobutanoate [**(S)-3**, 6.7 g], which was used for the next step without further purification. (caution: product is volatile). The concentrated product **(S)-3** (6.7 g) was dissolved in CH_2_Cl_2_ (130 mL), and Et_3_N (15.9 mL, 114.6 mmol, 2 equiv) was added. To the solution, cooled to 0 °C, was slowly added *p-*nitrobenzenesulfonyl chloride (12.6 g, 57.3 mmol, 1 equiv). The mixture was stirred for 30 min to complete the reaction. Then, the reaction mixture was washed with water (3 × 150 mL) and the combined organic phase was dried over Na_2_SO_4_ and concentrated under reduced pressure. The crude residue was purified by flash column chromatography to afford a title compound **(S)-4** (8.5 g, 38% overall yield for 3 steps) as yellowish powder. [α]20D +14.8 (c 3.13, ACN) for the (R)-enantiomer [α]20D −16.3 (c 3.0, ACN); TLC R*_f_* = 0.40 (EtOAc/hexane 2:3); ^1^H-NMR (300 MHz, CDCl_3_): δ 8.35 (d, *J* = 7.8 Hz, 2H), 8.06 (d, *J* = 7.8 Hz, 2H), 5.56 (d, *J* = 8.4 Hz, 2H), 3.77 (m, 1H), 3.63 (s, 3H), 2.48 (d, *J* = 5.4 Hz, 2H), 1.18 (d, *J* = 6.9 Hz, 3H). ^13^C-NMR (300 MHz, CDCl_3_): δ 171.69, 150.18, 147.12, 128.44 (×2), 124.58 (×2), 52.12, 47.21, 40.55, 21.35. Mass *m*/*z* calcd. for [M + Na]^+^ 325.05, found [M + Na]^+^ 325.0.

*Methyl (S)-3-((N-benzyl-4-nitrophenyl)sulfonamido)butanoate* [**(S)-5a**]. To a solution of **(S)-4** (3.0 g, 9.9 mmol) in DMF (40 mL) was added Cs_2_CO_3_ (5.8 g, 17.8 mmol, 1.8 equiv) and benzyl bromide (2.36 mL, 19.8 mmol, 2 equiv). The mixture was stirred for 2 h at room temperature. After completion of reaction, the mixture was diluted with water (100 mL), extracted with EtOAc (2 × 100 mL), washed with brine (100 mL), dried over Na_2_SO_4_ and concentrated under reduced pressure. The residue was purified by flash column chromatography to afford title compound **(S)-5a** (3.7 g, 95%) as yellowish powder. [α]20D +5.4 (c 3.0, ACN) for the (R)-enantiomer [α]20D −5.8 (c 3.25, ACN); TLC R*_f_* = 0.4 (EtOAc/hexane 1:3); ^1^H-NMR (500 MHz, CDCl_3_): δ 8.30 (d, *J* = 9.0 Hz, 2H), 7.94 (d, *J* = 9.0 Hz, 2H), 7.33–7.26 (m, 5H), 4.55 (d, *J* = 15.5 Hz, 1H), 4.46–4.40 (m, 1H), 4.33 (d, *J* = 15.0 Hz, 1H), 3.56 (s, 3H), 2.53 (dd, *J* = 6.5, 16.0 Hz, 1H), 2.26 (dd, *J* = 8.0, 16.0 Hz, 1H), 1.13 (d, *J* = 7.0 Hz, 3H). ^13^C-NMR (500 MHz, CDCl_3_): δ 171.01, 149.93, 146.92, 136.73, 128.84 (×2), 128.53 (×2), 128.41 (x2), 128.22, 124.38 (x2), 52.02, 51.96, 48.52, 40.66, 19.51. Mass *m*/*z* calcd. for [M + H]^+^ 393.1, found [M + H]^+^ 393.1.

*Methyl (S)-3-((N-(naphthalen-1-ylmethyl)-2-nitrophenyl)sulfonamido)butanoate* [**(S)-5b**]. Cs_2_CO_3_ (8.14 g, 25.0 mmol, 1.8 equiv) and 2-(Bromomethyl)naphthalene (4.61 gm, 20.8 mmol, 2 equiv) was added to a solution of **(S)-4** (4.2 g, 13.9 mmol) in DMF (45 mL). The mixture was stirred for 2 h at room temperature. After completion of the reaction, the mixture was diluted with water (100 mL), extracted with EtOAc (2 × 100 mL), washed with brine (100 mL), dried over Na_2_SO_4_ and concentrated under reduced pressure. The residue was purified by flash column chromatography to afford title compound **(S)-5b** (5.6 g, 92%) as a yellowish powder. [α]20D +7 (c 2.5, ACN); TLC R*_f_* = 0.42 (EtOAc/hexane 2:3); ^1^H-NMR (500 MHz, CDCl_3_): δ 8.17 (d, *J* = 8.1 Hz, 1H), 7.94 (d, *J* = 7.5 Hz, 1H), 7.83 (d, *J* = 8.4 Hz, 1H), 7.75 (d, *J* = 8.1 Hz, 1H), 7.63 (m, 2H), 7.58–7.46 (m, 4H), 7.40–7.34 (m, 1H), 5.06 (q, *J* =10.2 Hz, 2H), 4.64–4.53 (m, 1H), 3.55 (s, 3H), 2.58 (dd, *J* = 15.6, 5.1 Hz, 1H), 2.20 (dd, *J* = 15.6, 9.3 Hz, 1H), 1.18 (d, *J* = 6.6 Hz, 3H). ^13^C-NMR (500 MHz, CDCl_3_): δ 171.12, 147.90, 133.82, 133.72, 133.68, 132.38, 131.92, 131.79, 131.28, 129.01, 128.75, 127.12, 126.74, 126.09, 125.34, 124.38, 123.03, 51.97, 51.65, 46.21, 40.24, 18.95. Mass *m*/*z* calcd. for [M + Na]^+^ 465.11, found [M + Na]^+^ 465.1.

*(S)-3-((N-benzyl-4-nitrophenyl)sulfonamido)butanoic acid* [**(S)-6a**] and [**(R)-6a**]. The compound **(S)-5a** (3.5 g, 8.93 mmol) was dissolved in THF/water (20 mL/10 mL), cooled to 0 °C, and a solution of LiOH (0.24 g, 9.82 mmol, 1.1 equiv) in water (10 mL) was subsequently added. The mixture was stirred at room temperature for 2 h. After complete consumption of the starting material, volatiles were removed from the reaction mixture using a rotary evaporator and the remaining aqueous layer was washed with diethyl ether (2 × 60 mL). The aqueous phase was acidified with 1N HCl solution until the pH of solution reached ~2–3. The precipitated compound was extracted by EtOAc (3 × 40 mL). The organic layer was dried over Na_2_SO_4_ and concentrated. The remaining residue was dried in vacuo to afford title compound **(S)-6a** (2.05 g, 60%) as a white powder. [α]20D +8.3 (c 3.0, ACN/CHCl_3_ 2:1); TLC R*_f_* = 0.25 (EtOAc/hexane/AcOH 1:2:0.1); ^1^H-NMR (500 MHz, CDCl_3_): δ 8.27 (d, *J* = 9.0 Hz, 2H), 7.93 (d, *J* = 9.0 Hz, 2H), 7.37–7.30 (m, 5H), 4.52–4.33 (m, 3H), 2.58 (dd, *J* = 7.2, 16.2 Hz, 1H), 2.27 (dd, *J* = 7.2, 16.2 Hz, 1H), 1.12 (d, *J* = 6.9 Hz, 3H). ^13^C-NMR (500 MHz, CDCl_3_): δ 176.84, 149.99, 146.80, 136.71, 128.97 (×2), 128.62 (×2), 128.45 (×2), 128.39, 124.39 (×2), 51.78, 48.54, 40.23, 20.15. Mass *m*/*z* calcd. for [M + Na]^+^ 401.08, found [M + Na]^+^ 401.1. HRMS (HR-FAB-MS) *m*/*z*: calcd for C_17_H_18_N_2_O_6_S [M + H]^+^ 379.0964; found 379.0964. (*R*)-Enantiomer **(R)-6a** was synthesized by the same procedure as described above. [α]20D −8.7 (c 3.0, ACN/CHCl_3_ 2:1); ^1^H-NMR (600 MHz, CDCl_3_): δ 8.31 (dd, *J* = 7.2 Hz, 1.8 Hz, 2H), 7.96 (dd, *J* = 7.2 Hz, 1.8 Hz, 2H), 7.39–7.33 (m, 5H), 4.54–4.4 (m, 3H), 2.61 (dd, *J* = 6.6, 16.2 Hz, 1H), 2.30 (dd, *J* = 7.8, 16.2 Hz, 1H), 1.16 (d, *J* = 6.6 Hz, 3H). ^13^C-NMR (600 MHz, CDCl_3_): δ 175.58, 149.86, 146.70, 136.51, 128.76 (×2), 128.44 (×2), 128.27 (×2), 128.18, 124.16 (×2), 51.66, 48.44, 40.00, 19.84. Mass *m*/*z* calcd, for [M + Na]^+^ 401.08, found [M + Na]^+^ 401.1. HRMS (HR-FAB-MS) *m*/*z*: calcd for C_17_H_18_N_2_O_6_S [M + H]^+^ 379.0964; found 379.0961.

*(S)-3-((N-(naphthalen-1-ylmethyl)-2-nitrophenyl)sulfonamido)butanoic acid* [**(S)-6b**]. The compound **(S)-5b** (4.6 g, 10.4 mmol) was dissolved in THF/water (24 mL/12 mL), cooled to 0 °C, and a solution of LiOH (0.28 g, 11.44 mmol, 1.1 equiv) in water (12 mL) was subsequently added. The mixture was stirred at room temperature for 2 h. After the complete consumption of the starting material, volatiles were removed from the reaction mixture using a rotary evaporator and the remaining aqueous layer was washed with diethyl ether (2 × 60 mL). The aqueous phase was acidified with 1 N HCl solution until the pH of solution reached ~2–3. The precipitated compound was extracted by EtOAc (3 × 50 mL). The organic layer was dried over Na_2_SO_4_ and concentrated. The crude was dried in vacuo to afford title compound **(S)-6b** (2.49 g, 56%) as an off-white solid. [α]20D +8.1 (c 3.0, ACN/CHCl_3_ 2:1); TLC R*_f_* = 0.25 (EtOAc/hexane/AcOH 1:2:0.1); ^1^H-NMR (500 MHz, CDCl_3_): δ 8.17 (d, *J* = 8.5 Hz, 1H), 7.92 (d, *J* = 7.5 Hz, 1H), 7.84 (d, *J* = 7.5 Hz, 1H), 7.76 (d, *J* = 8.0 Hz, 1H), 7.63 (m, 2H), 7.58–7.48 (m, 4H), 7.39–7.36 (m, 1H), 5.06 (q, *J* = 16 Hz, 2H), 4.59–4.54 (m, 1H), 2.62 (dd, *J* = 15.5, 4.5 Hz, 1H), 2.22 (dd, *J* = 16.0, 9.0 Hz, 1H), 1.20 (d, *J* = 6.5 Hz, 3H). ^13^C-NMR (300 MHz, CDCl_3_): δ 176.09, 147.89, 133.85, 133.81, 133.59, 132.24, 131.87, 131.30, 129.07, 128.87, 127.21, 126.82, 126.15, 125.37, 123.02, 51.37, 46.28, 40.11, 31.17, 19.05. Mass *m*/*z* calcd. for [M + Na]^+^ 451.09 found [M + Na]^+^ 451.1. HRMS (HR-FAB-MS) *m*/*z*: calcd for C_21_H_20_N_2_O_6_S [M + H]^+^ 429.1120; found 429.1116.

Note: *Para*-nosyl group was used for all nosyl protected compounds except for **(S)-5b** and **(S)-6b** where *ortho*-nosyl group was used. No notable difference was observed throughout the syntheses (solution/solid-phase) between *ortho*- and *para*-nosyl protecting group.

#### 3.2.2. General Solid-Phase Synthesis Method for Oligomers Synthesis 

Solid-phase synthesis was conducted in a 3 to 5 mL fritted syringe (Torviq, Tucson, AZ, USA). Rink amide MBHA resin (100 mg, 0.045 mmol) was swelled with DMF (1 mL) for 2 h. Fmoc protecting group of resin (100 mg, 0.045 mmol) was removed by 20% piperidine in DMF (2 × 10 min). For sub-monomer coupling, resins were treated with the solution of synthesized monomer **6** (3.0 equiv), HATU (3.0 equiv), HOAt (3.0 equiv), and DIPEA (4.5 equiv) in DMF (1.0 mL) at room temperature. After shaking for 3 h, the reaction mixture was drained, and the resin was washed with DMF (×5) and CH_2_Cl_2_ (×5). Then, the nosyl protecting group was removed by treating with 2-mercaptoethanol (2% in DMF, 0.6 mL) and 1,8-diazabicycloundec-7-ene (2.1% in DMF, 0.6 mL) for 45 min (×2) at room temperature. After a thorough washing cycle, the next monomer conjugation and nosyl deprotection steps were repeated to provide oligomers with the desired chain length. For *N*-terminal acetylation, oligomers on solid support were treated with acetic anhydride (10 equiv) and DIPEA (10 equiv) in DMF (1 mL) for 2 h at room temperature. Final products were cleaved from the resin using a cleavage cocktail (95% TFA, 2.5% triisopropylsilane, and 2.5% water) for 2 h (1 h × 2) at room temperature and purified by reverse-phase HPLC.

## 4. Conclusions

In summary, we have developed a facile and efficient solid-phase method for the synthesis of β-ABpeptoids, which consists of oligomers of *N*-substituted β^3^-alanines. Iterative on-resin coupling reactions of *N*-alkylated β^3^-homoalanines as monomer building blocks allow for the preparation of β-ABpeptoid oligomers ranging from 2- to 8-mers in excellent yields. Although our solid-phase method is highly efficient, each monomer building blocks should be synthesized individually via a somewhat cumbersome solution-phase synthesis and purification process. We are currently developing a submonomer solid-phase synthetic method that does not require the preparation of each monomers, and the results will be reported in due course. Notably, β-ABpeptoids have backbone chirality in contrast to regular peptoids, and thus, are anticipated to form ordered structures. Indeed, these β-ABpeptoids with longer than 4-mer exhibited characteristic CD spectra and increased NOE signals, suggesting that they adopt well-defined folding structures. Further structural studies including X-ray study are needed to determine their detailed 3-dimensional structure. Given their potential to form folding conformation, along with advantages as peptoids such as good cell permeability and stability, β-ABpeptoids represent a novel class of peptidomimetic foldamers and will find a wide range of applications in biomedical and material sciences.

## Figures and Tables

**Table 1:**
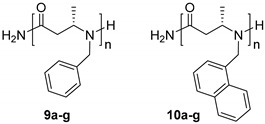
Structures and analytical data of synthesized (*S*) form of β-ABpeptoid oligomers.

Compd No.	Chain Length	% Purity ^a^	Calcd Mass	Obsd Mass ^b^
**(S)-9a**	2	65	367.23	368.2 [M + H]^+^
**(S)-9b**	3	68	542.33	543.3 [M + H]^+^
**(S)-9c**	4	77	717.43	718.4 [M + H]^+^
**(S)-9d**	5	78	892.53	893.4 [M + H]^+^
**(S)-9e**	6	79	1067.62	1068.5 [M + H]^+^
**(S)-9f**	7	73	1242.72	1243.6 [M + H]^+^
**(S)-9g**	8	71	1417.82	1440.9 [M + H]^+*c*^
**(S)-9e-Ac ^d^**	6	70	1109.64	1132.5 [M + H]^+^
**(S)-10a**	2	94	467.26	468.2 [M + H]^+^
**(S)-10b**	3	82	692.37	693.4 [M + H]^+^
**(S)-10c**	4	94	917.49	918.4 [M + H]^+^
**(S)-10d**	5	92	1142.60	1144.5 [M + H]^+^
**(S)-10e**	6	87	1367.72	1368.6 [M + H]^+^
**(S)-10f**	7	85	1592.83	1614.8 [M + Na]^+*c*^
**(S)-10g**	8	81	1817.95	1839.9 [M + Na]^+*c*^

^a^ Determined by analytical reversed-phase HPLC of crude products. ^b^ Mass spectrometry data were acquired using ESI techniques. ^c^ Mass spectrometry data were acquired using MALDI technique. ^d^ *N*-terminal was acetylated.

## Supplementary Materials

The following are available online: supplemental figures and tables regarding characterization data including HPLC chromatograms, mass spectrometry data, CD spectra, and NMR data.
