# Supplementary material for: Solid-Phase Synthesis and Circular Dichroism Study of β-ABpeptoids
Source: Molecules. 2019 Jan 5;24(1):178. doi: 10.3390/molecules24010178 (PMC6337665; doi:10.3390/molecules24010178)

Supporting Information for

Solid-phase Synthesis and Circular Dichroism Study of β-ABpeptoids

Ganesh A. Sable, Kang Ju Lee, and Hyun-Suk Lim*

Department of Chemistry and Division of Advanced Material Science, Pohang University of Science and Technology (POSTECH), Pohang 37673, South Korea

***Correspondence: hslim@postech.ac.kr

TABLE OF CONTENTS

1. Optical rotations of the products and intermediates (Table S1**)....................................** S03

2. Chiral HPLC analysis of compound **(S)/(R)-5a**, **(S)/(R)-6a** (Figure S1**) ……….......** S04

3. Isolated yields and nature of synthesized *β*-ABpeptoid oligomers (Table S2) **…........** S06

4. Synthesized *β*-ABpeptoid oligomer sequence, purity, and mass data (Table S3)**…......** S07

5. HRMS data for Synthesized *β*-ABpeptoid oligomers (Table S4)**……………...…......** S08

6. LC/MS spectra of crude product of nosyl protected dimer **(S)-9a-Ns (**Figure S2)**..….** S09

7. HPLC chromatograms of crude products **(S)-9a-g** and **(S)-10a-g** (Figure S3)**….........** S10

8. LC/MS spectra of purified oligomers **(S)-** and **(R)-9a-g,** and **(S)-10a-g** (Figure S4)**…** S14

9. CD spectroscopic data (Figure S5-S8)**…………………………………………...** S38

10. NMR data for; **(R)-1**, **(S)-4**, **(S)-5a**, **(S)-5b**, **(S)/(R)-6a**, and **(S)-6b** (Figure S9) **……** S40

11. NOESY spectra for **(R)-9a**, **(R)-9b** and, 4mer **(R)-9c** (Figure S10) **…...……….......** S58

Table S1. The optical rotations of (S)- and (R)-forms of the products and intermediates

structure	(S)-isomer	(R)-isomer
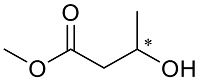	+22.9 ^{o} (ACN)	-23.3 ^{o} (ACN)
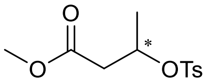	+7.8 ^{o} (ACN)	-8.3 ^{o} (ACN)
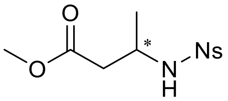	+14.8 ^{o} (ACN)	-16.3 ^{o} (ACN)
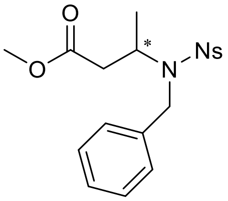	+5.4 ^{o} (ACN)	-5.8 ^{o} (ACN)
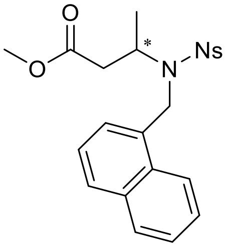	+7.0 ^{o} (ACN)	not synthesized
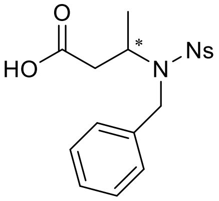	+8.3 ^{o} (ACN/$CHCl_3$ 2:1)	-8.7 ^{o} (ACN/$CHCl_3$ 2:1)
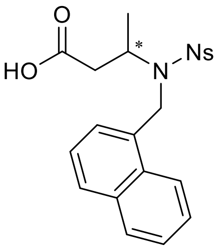	+8.1 ^{o} (ACN/$CHCl_3$ 2:1)	not synthesized

Figure S1. Chiral HPLC analyses of compounds **(S)/(R)-5a**, and **(S)/(R)-6a.**

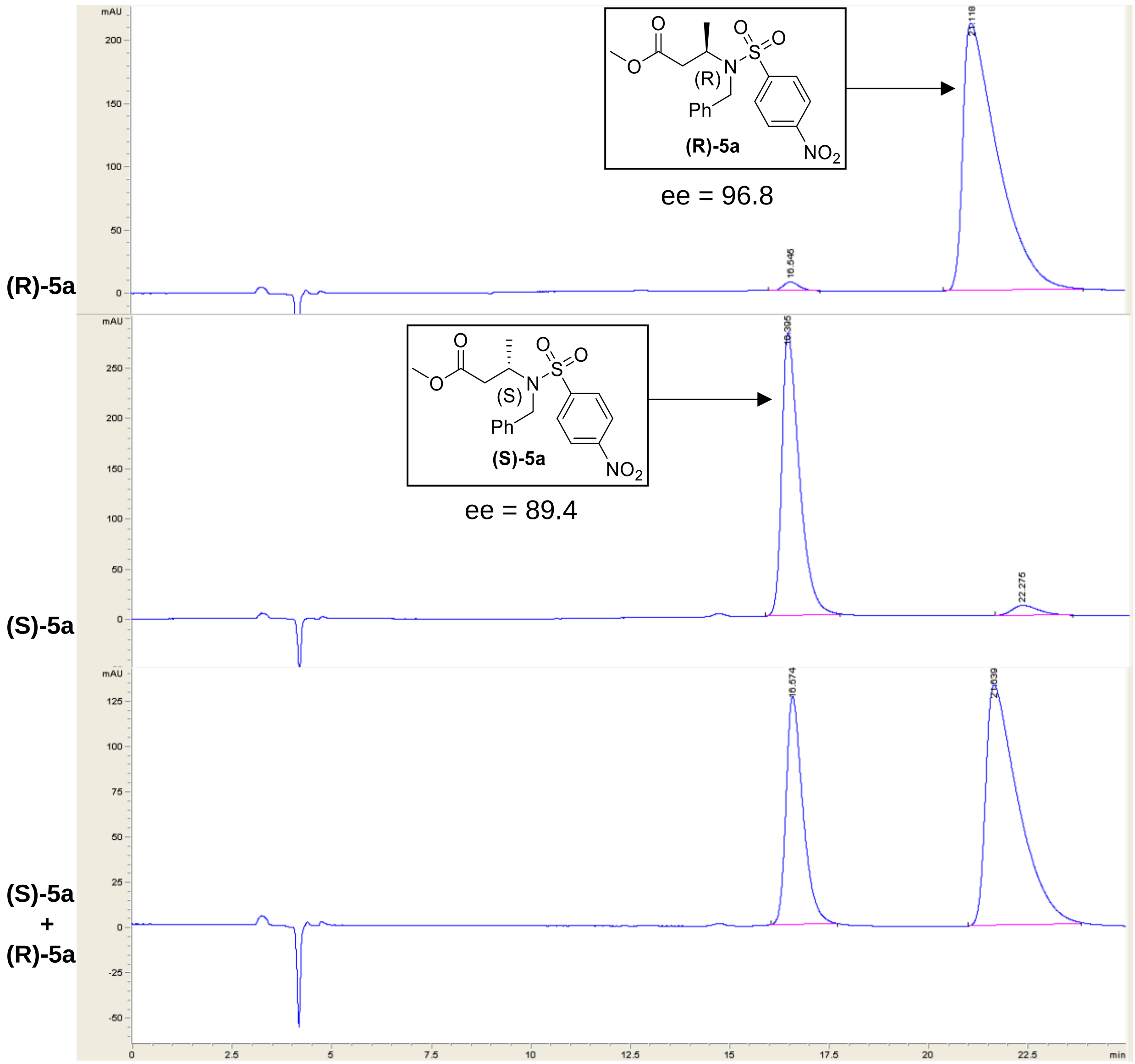

Figure S1, continued

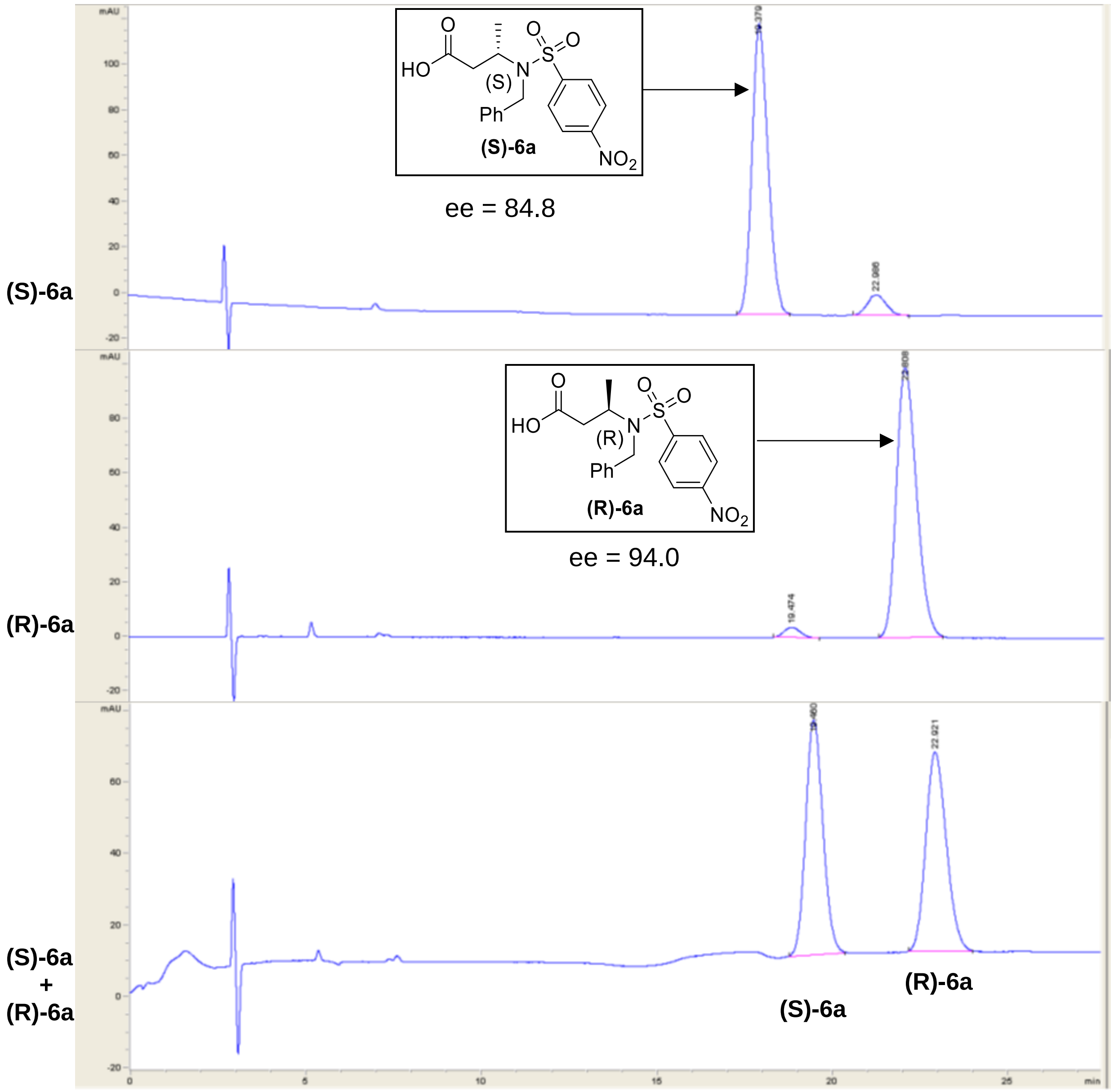

Table S2. Isolated yields and nature of synthesized β-ABpeptoid oligomers

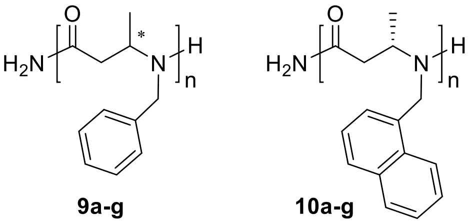

9a-g **10a-g**

compd. no.	resin Qty. (mg)[a]	isolated product Qty. (mg)[b]	isolated product yield (%)[c]	compound nature
**(S)-9a**	40	4.8	72%	colorless sticky oil
**(S)-9b**	40	6.8	70%	colorless sticky semi-solid
**(S)-9c**	40	8.9	69%	white solid
**(S)-9d**	30	8.2	68%	white solid
**(S)-9e**	30	10.4	72%	white solid
**(S)-9f**	20	7.3	66%	white solid
**(S)-9g**	20	8.1	63%	white solid
**(S)-9e-Ac**[d]	40	12.6	64%	white solid
**(R)-9a**	30	3.5	71%	colorless sticky oil
**(R)-9b**	30	5.3	73%	colorless sticky semi-solid
**(R)-9c**	30	6.5	67%	white solid
**(R)-9d**	20	5.0	62%	white solid
**(R)-9e**	20	6.1	63%	white solid
**(R)-9f**	20	6.6	59%	white solid
**(R)-9g**	20	7.2	57%	white solid
**(R)-9e-Ac**[d]	20	5.7	58%	white solid
**(S)-10a**	25	4.9	91%	colorless sticky oil
**(S)-10b**	25	6.0	78%	white semi-solid
**(S)-10c**	20	9.1	89%	white solid
**(S)-10d**	20	7.8	76%	white solid
**(S)-10e**	20	8.6	70%	white solid
**(S)-10f**	20	9.6	68%	white solid
**(S)-10g**	20	10.3	63%	white solid
**(S)-10e-Ac**[d]	20	7.6	60%	white solid

[a]Initial resin loading was 0.45 mmol/gm. [b]Determined based on 100% initial resin loading of the first residue. [c]Quantities of isolated products are given after lyophilization of purified products. [d]*N*-terminal is acetylated.

Table S3. Synthesized *β*-ABpeptoid Oligomer Sequence, Purity, and Mass Confirmation

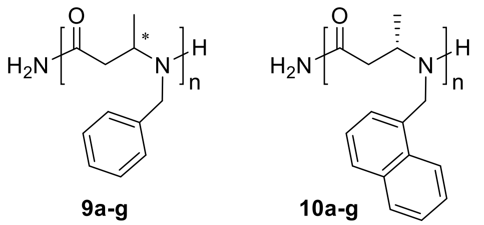

compd no.	chain length	% purity[a]	calcd mass	obsd mass[b]
**(S)-9a**	2	99	367.23	368.2 $[M+H]^+$
**(S)-9b**	3	98	542.33	543.3 $[M+H]^+$
**(S)-9c**	4	98	717.43	718.4 $[M+H]^+$
**(S)-9d**	5	99	892.53	893.4 $[M+H]^+$
**(S)-9e**	6	97	1067.62	1068.5 $[M+H]^+$
**(S)-9f**	7	99	1242.72	1243.6 $[M+H]^+$
**(S)-9g**	8	99	1417.82	1440.9 $[M+Na]^{+c}$
**(S)-9e-Ac**[d]	6	99	1109.64	1132.5 $[M+H]^+$
**(R)-9a**	2	97	367.23	368.2 $[M+H]^+$
**(R)-9b**	3	98	542.33	543.3 $[M+H]^+$
**(R)-9c**	4	98	717.43	718.4 $[M+H]^+$
**(R)-9d**	5	97	892.53	893.5 $[M+H]^+$
**(R)-9e**	6	98	1067.62	1068.6 $[M+H]^+$
**(R)-9f**	7	99	1242.72	1265.8 $[M+Na]^{+c}$
**(R)-9g**	8	98	1417.82	1439.8 $[M+H]^{+c}$
**(R)-9e-Ac**[d]	6	98	1109.64	1133.5 $[M+Na]^+$
**(S)-10a**	2	99	467.26	468.2 $[M+H]^+$
**(S)-10b**	3	98	692.37	693.4 $[M+H]^+$
**(S)-10c**	4	99	917.49	918.4 $[M+H]^+$
**(S)-10d**	5	99	1142.60	1144.5 $[M+H]^+$
**(S)-10e**	6	99	1367.72	1368.6 $[M+H]^+$
**(S)-10f**	7	99	1592.83	1614.8 $[M+Na]^{+c}$
**(S)-10g**	8	97	1817.95	1839.9 $[M+Na]^{+c}$
**(S)-10e-Ac**[d]	6	98	1409.73	1431.7 $[M+Na]^{+c}$

[a]Determined by analytical reversed-phase HPLC of purified products. [b]Mass spectrometry data were acquired using ESI techniques. [c]Mass spectrometry data were acquired using MALDI-TOF technique. [d]*N*-terminal is acetylated.

Table S4. HRMS Data for Synthesized β-ABpeptoid Oligomers

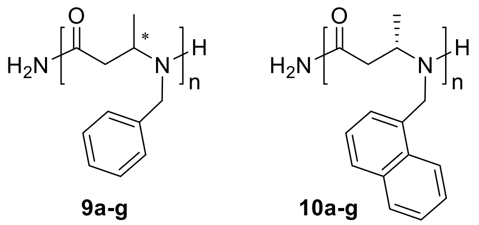

9a-g **10a-g**

compd no.	chain length	chemical formula	calcd mass	obsd mass
**(S)-9a**	2	$C_{22}H_{29}N_3O_2$	368.2338 $[M+H]^+$	368.2339
**(S)-9b**	3	$C_{33}H_{42}N_4O_3$	543.3335 $[M+H]^+$	543.3332
**(S)-9c**	4	$C_{44}H_{55}N_5O_4$	718.4332 $[M+H]^+$	718.4334
**(S)-9d**	5	$C_{55}H_{68}N_6O_5$	893.5329 $[M+H]^+$	893.5332
**(S)-9e**	6	$C_{66}H_{81}N_7O_6$	1068.6327 $[M+H]^+$	1068.6323
**(S)-9f**	7	$C_{77}H_{94}N_8O_7$	1243.7324 $[M+H]^+$	1243.7321
**(S)-9g**	8	$C_{88}H_{107}N_9O_8$	1418.8321 $[M+H]^+$	1418.8329
**(S)-9e-Ac**[a]	6	$C_{68}H_{83}N_7O_7$	1132.6252 $[M+Na]^+$	1132.6252
**(R)-9a**	2	$C_{22}H_{29}N_3O_2$	368.2338 $[M+H]^+$	368.2335
**(R)-9b**	3	$C_{33}H_{42}N_4O_3$	543.3335 $[M+H]^+$	543.3334
**(R)-9c**	4	$C_{44}H_{55}N_5O_4$	718.4332 $[M+H]^+$	718.4329
**(R)-9d**	5	$C_{55}H_{68}N_6O_5$	893.5329 $[M+H]^+$	893.5333
**(R)-9e**	6	$C_{66}H_{81}N_7O_6$	1068.6327 $[M+H]^+$	1068.6330
**(R)-9f**	7	$C_{77}H_{94}N_8O_7$	1243.7324 $[M+H]^+$	1243.7329
**(R)-9g**	8	$C_{88}H_{107}N_9O_8$	1418.8321 $[M+H]^+$	1418.8328
**(R)-9e-Ac**[a]	6	$C_{68}H_{83}N_7O_7$	1132.6252 $[M+Na]^+$	1132.6247
**(S)-10a**	2	$C_{30}H_{33}N_3O_2$	468.2651 $[M+H]^+$	468.2653
**(S)-10b**	3	$C_{45}H_{48}N_4O_3$	693.3805 $[M+H]^+$	693.3808
**(S)-10c**	4	$C_{60}H_{63}N_5O_4$	918.4958 $[M+H]^+$	918.4956
**(S)-10d**	5	$C_{75}H_{78}N_6O_5$	1143.6112 $[M+H]^+$	1143.6110
**(S)-10e**	6	$C_{90}H_{93}N_7O_6$	1368.7266 $[M+H]^+$	1368.7272
**(S)-10f**	7	$C_{105}H_{108}N_8O_7$	1593.8419 $[M+H]^+$	1593.8427
**(S)-10g**	8	$C_{120}H_{123}N_9O_8$	1818.9573 $[M+H]^+$	1818.9580

[a]*N*-terminal is acetylated. ***Note***- High Resolution Mass Spectrometry (HRMS) data were acquired using Fast Atom Bombardment (FAB^+) ionization techniques.

Figure S2. LC/MS spectra of crude product of nosyl protected dimer **(S)-9a-Ns**

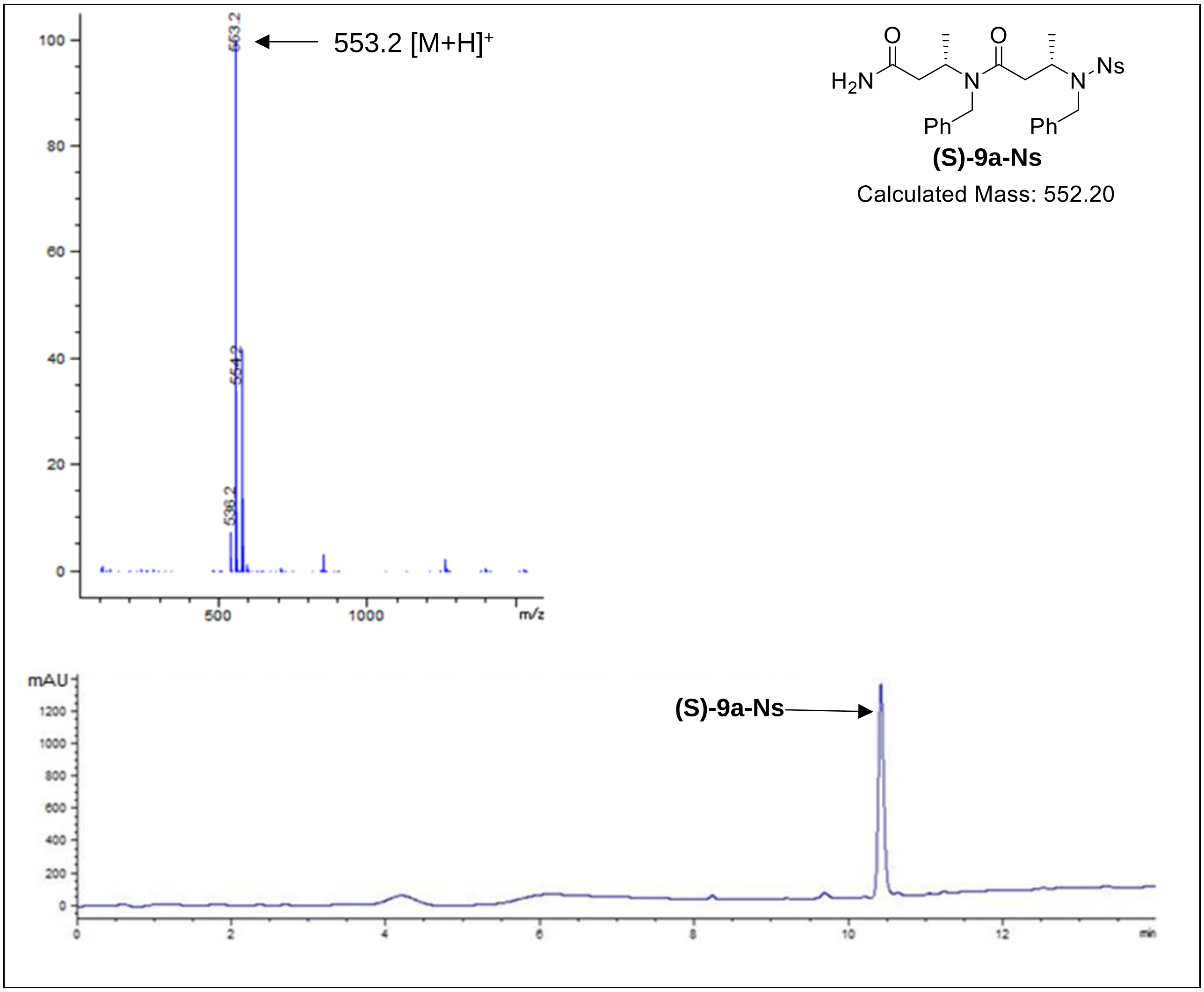

Figure S3. HPLC chromatograms of crude products **(S)-9a-g** and **(S)-10a-g**.

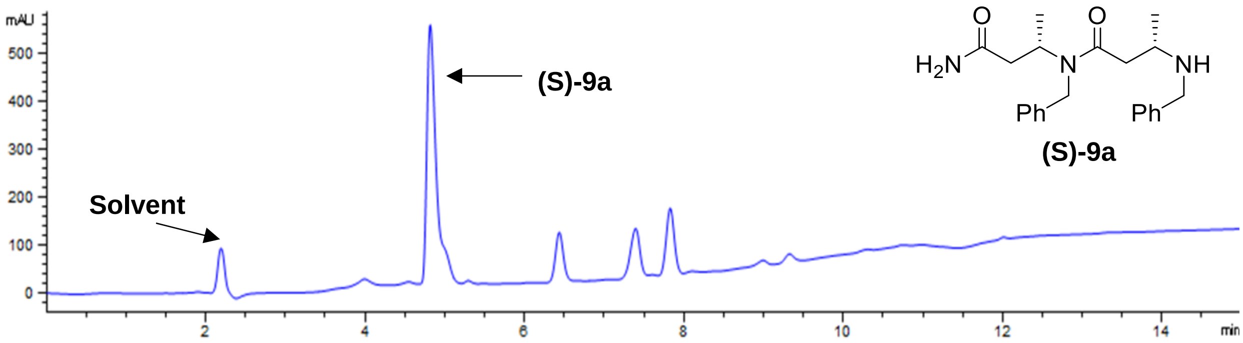

Figure S3, continued

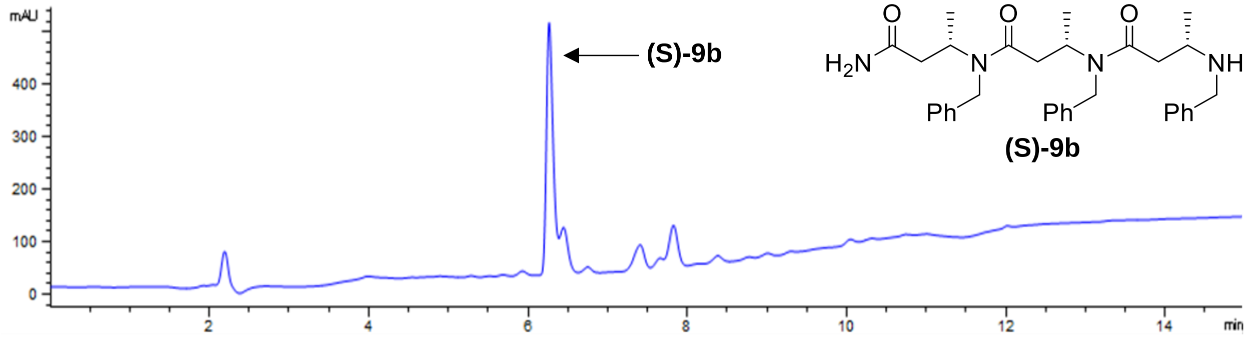

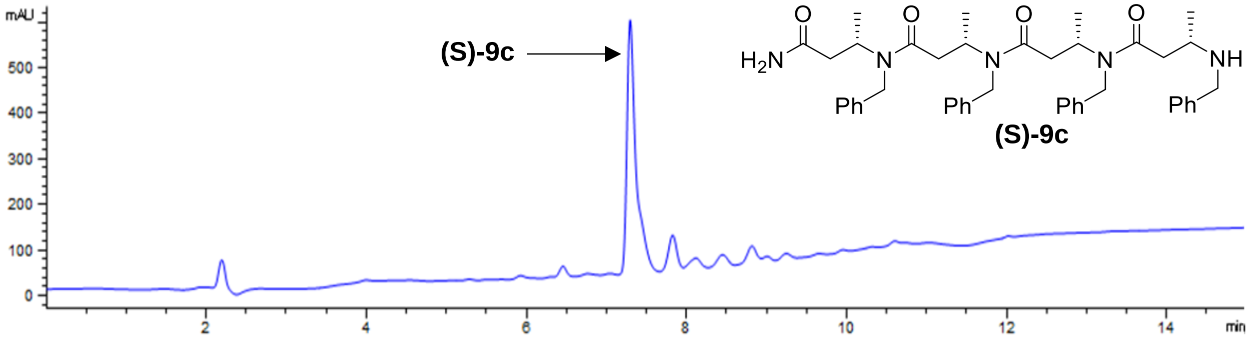

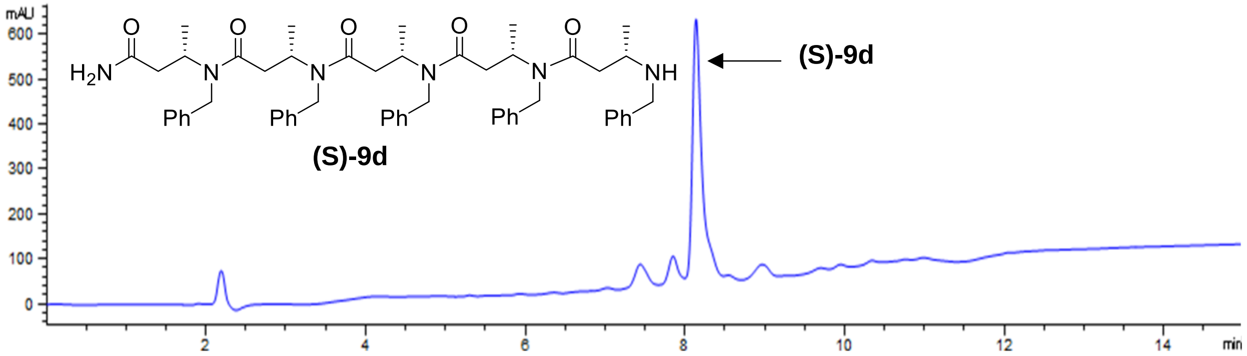

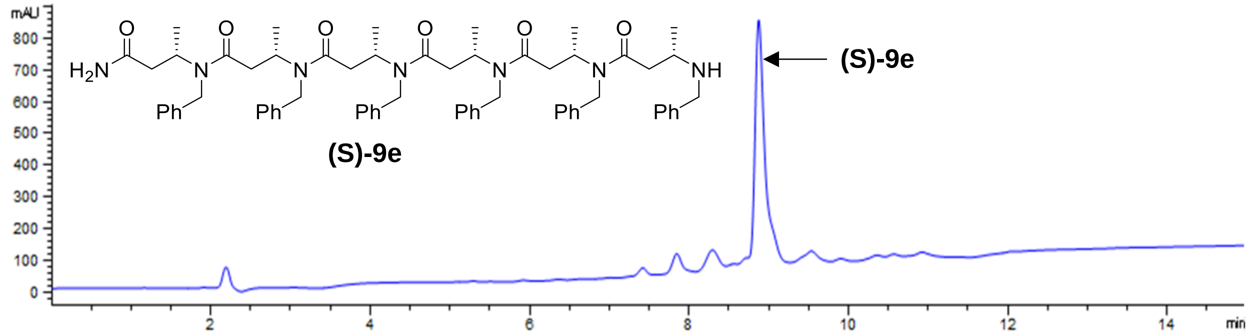

Figure S3, continued

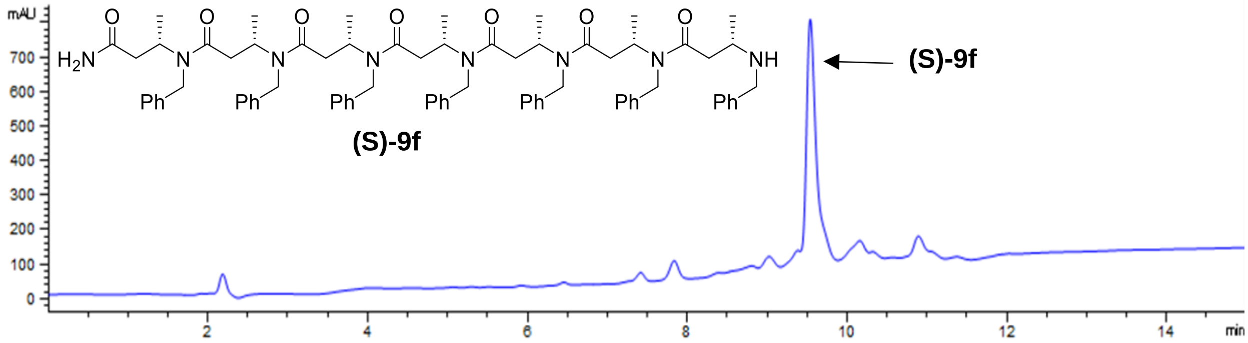

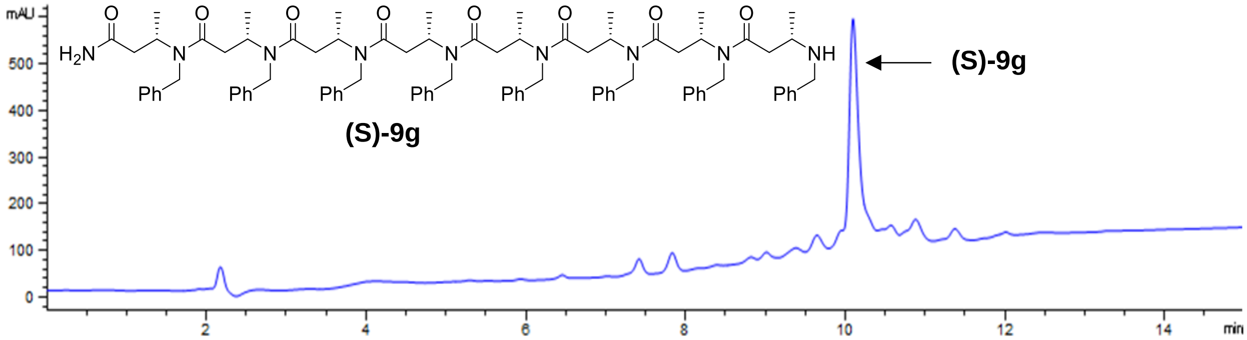

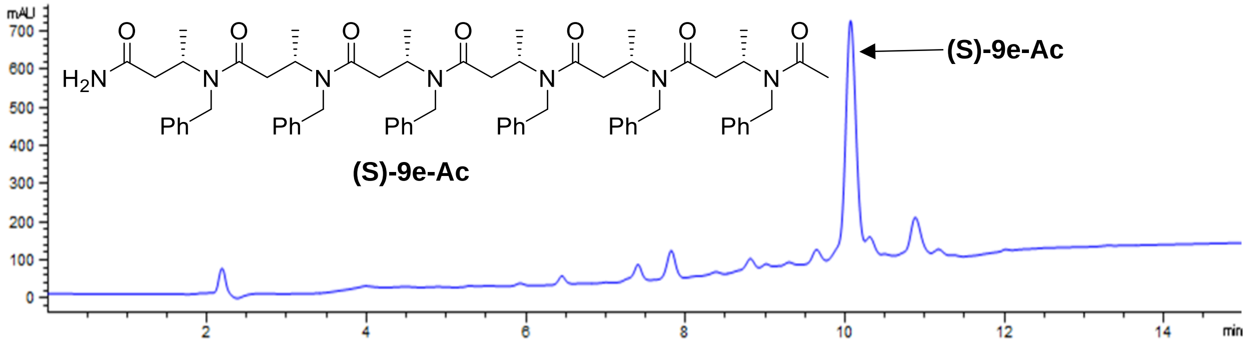

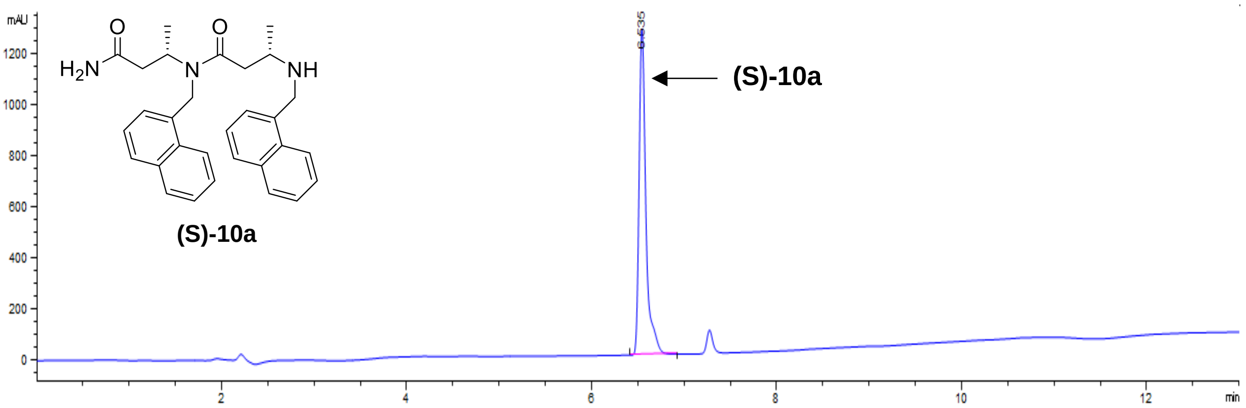

Figure S3, continued

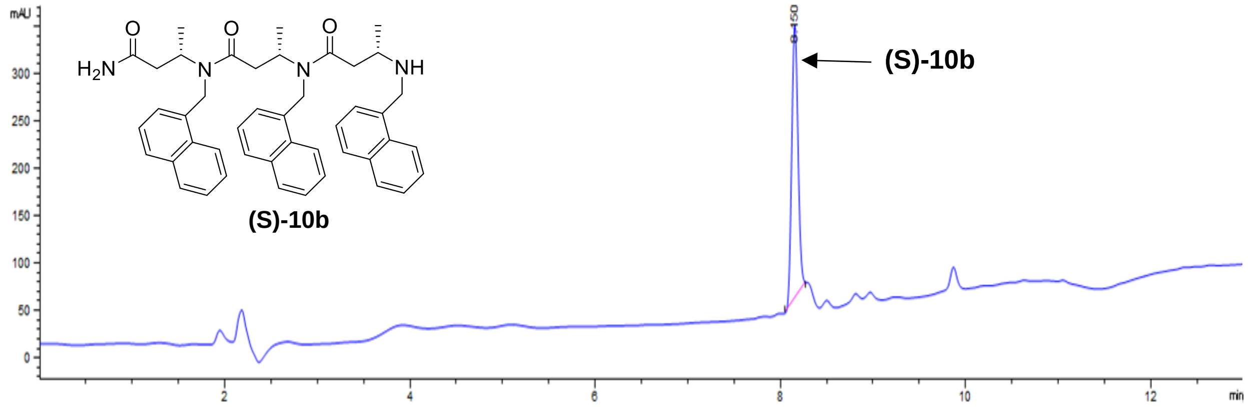

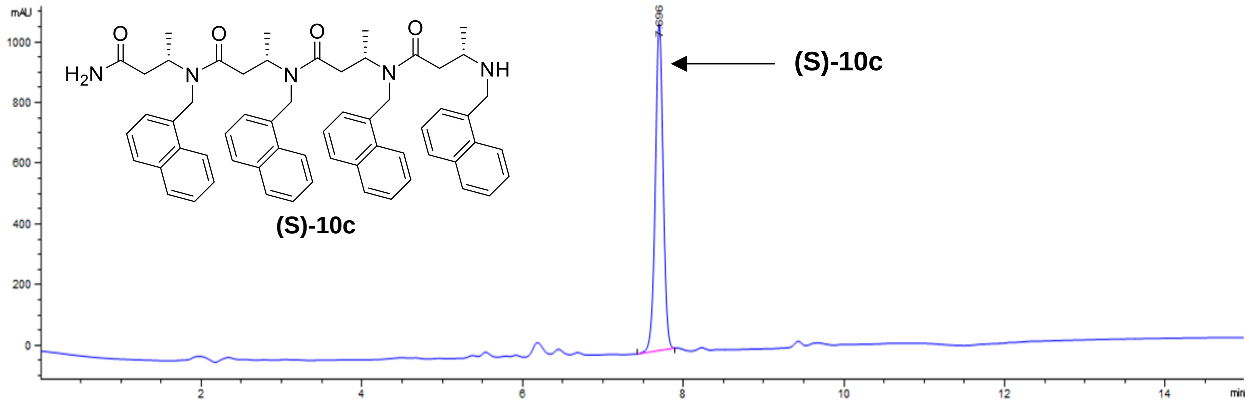

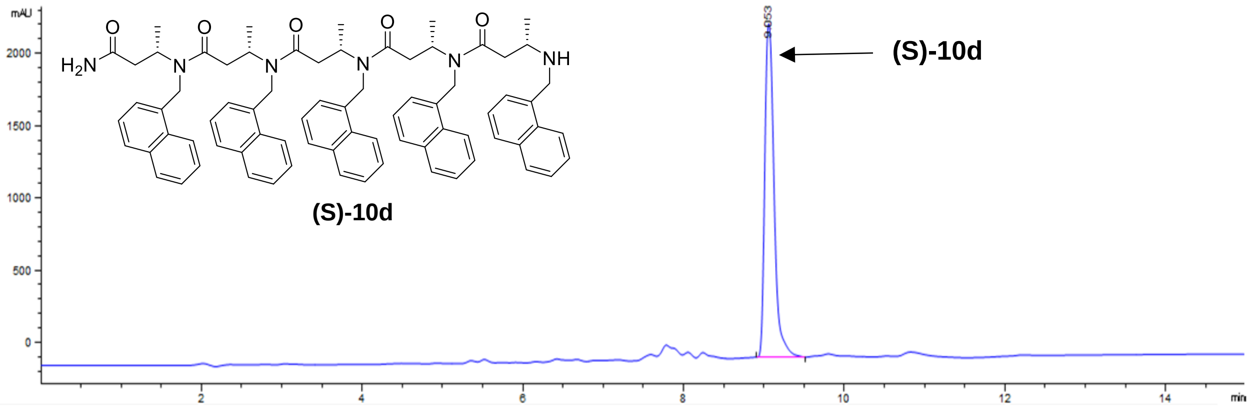

Figure S3, continued

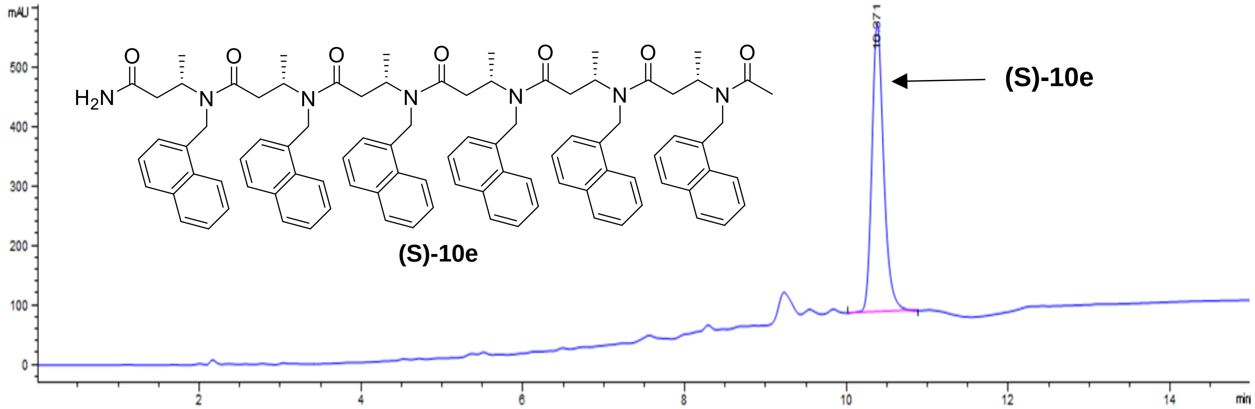

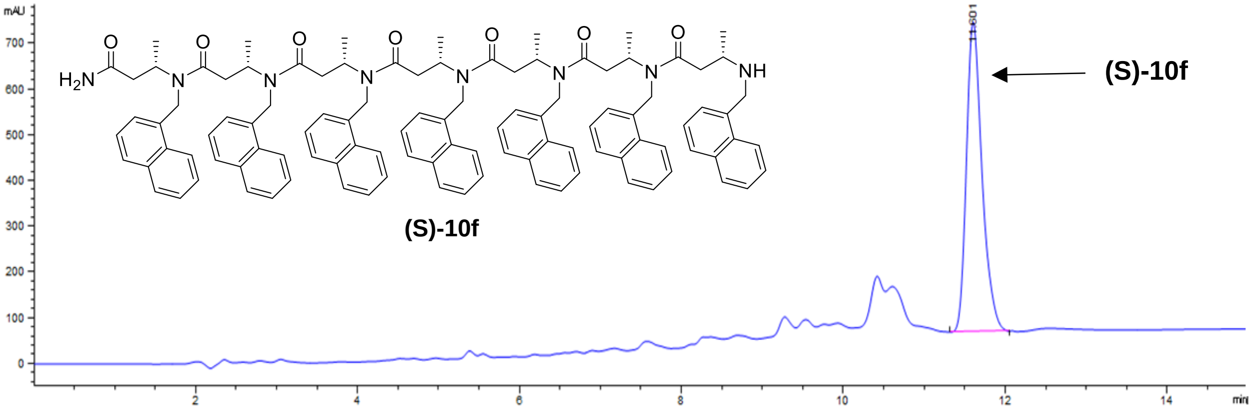

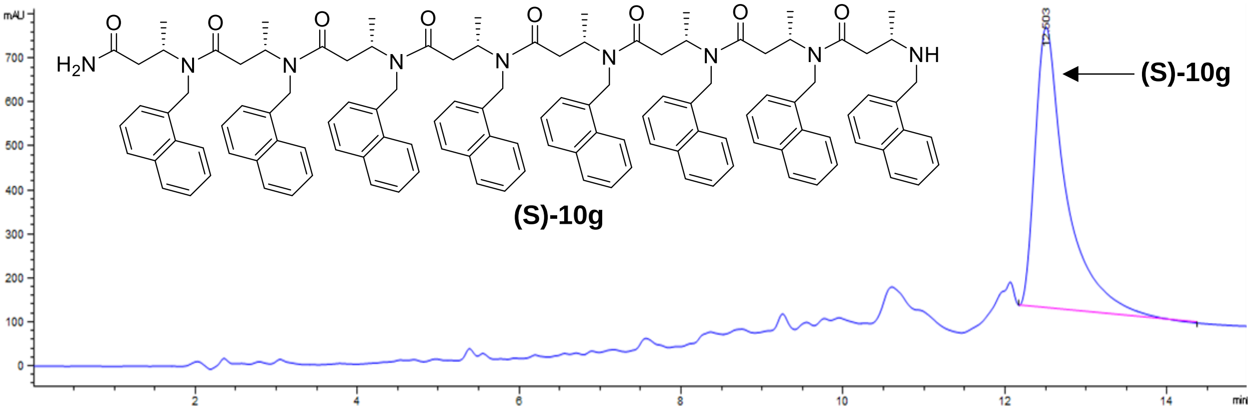

Figure S4. LC/MS spectra of purified oligomers **(S)-** and **(R)-9a-g,** and **(S)-10a-g**.

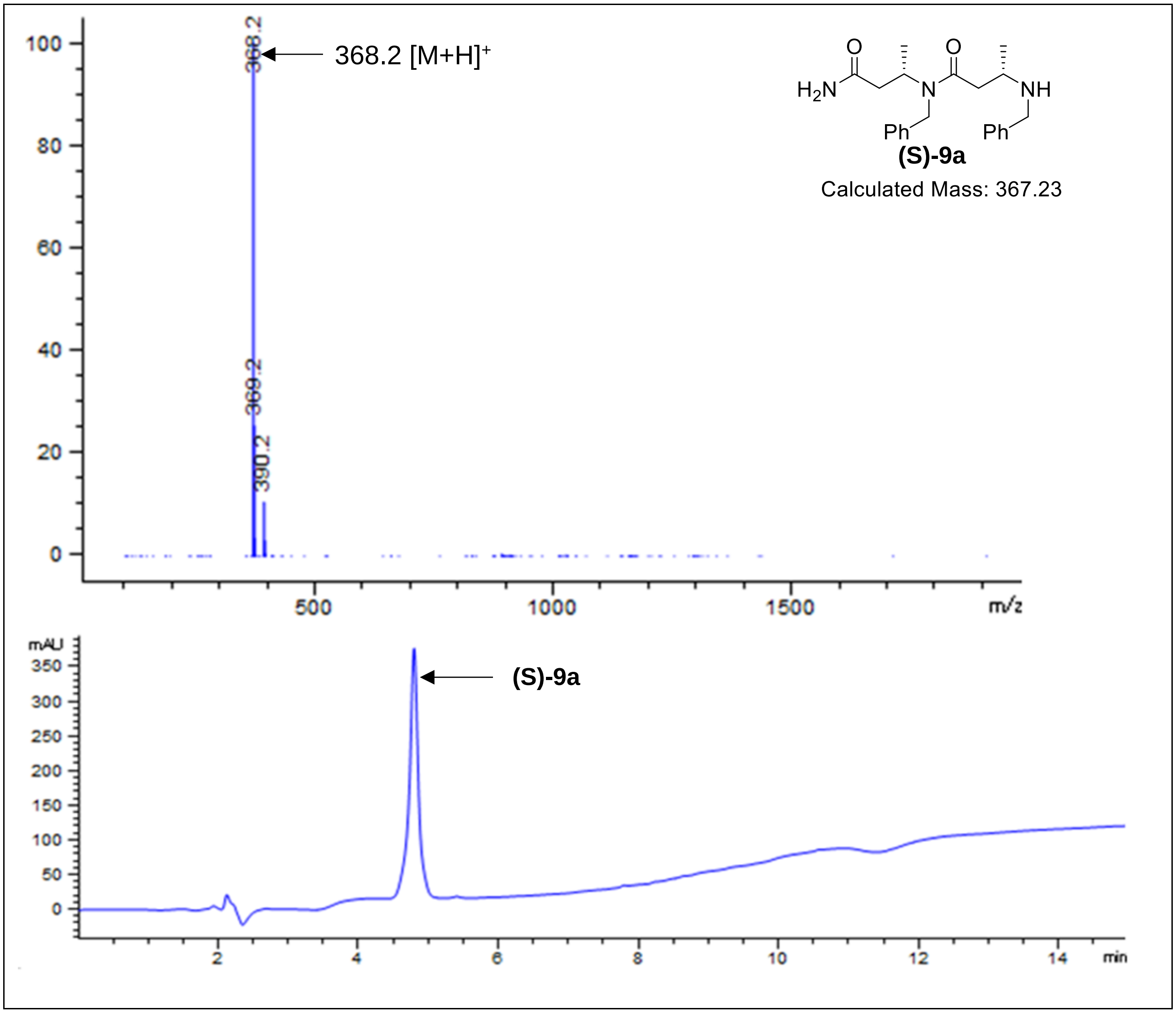

Figure S4, continued

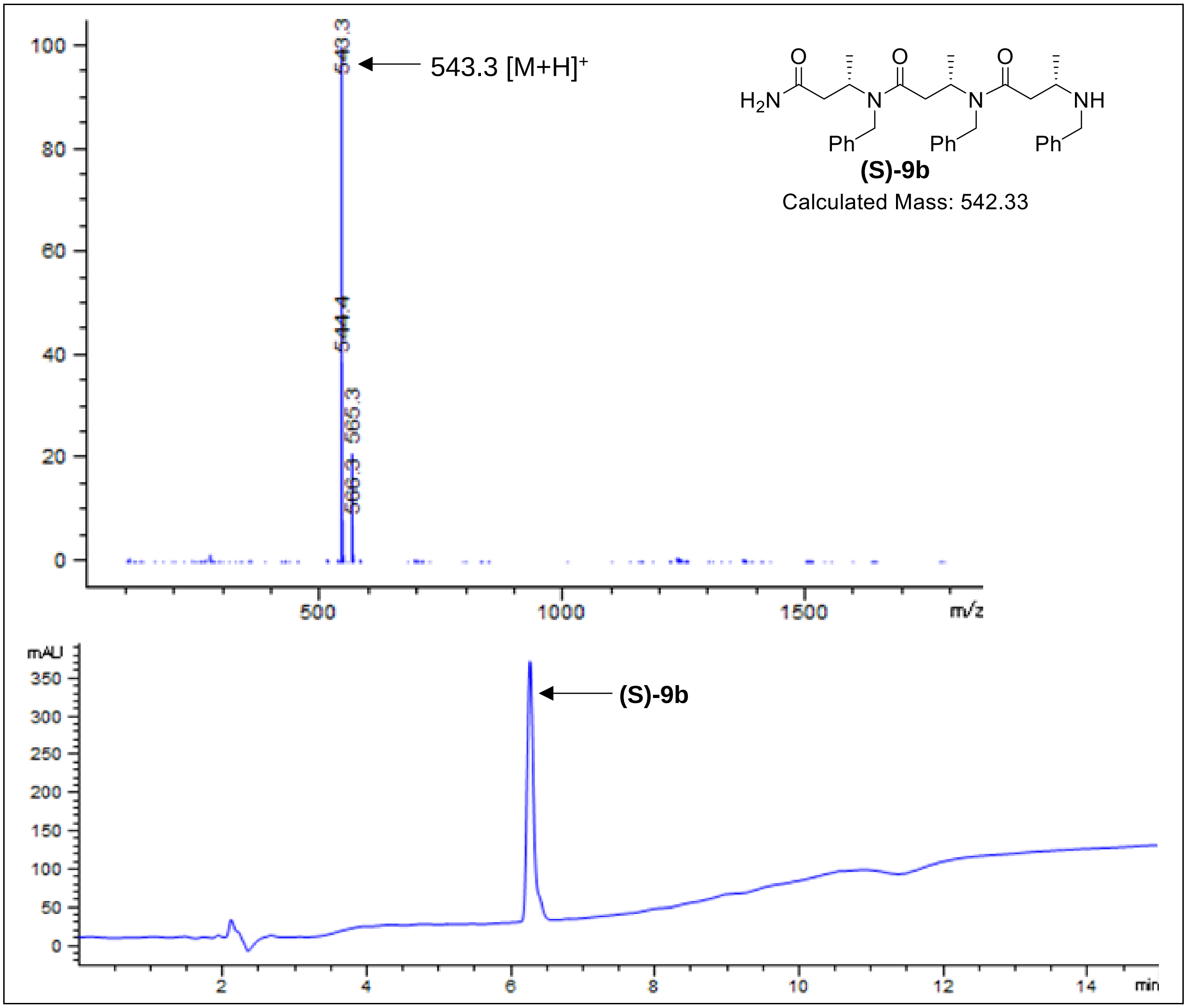

Figure S4, continued

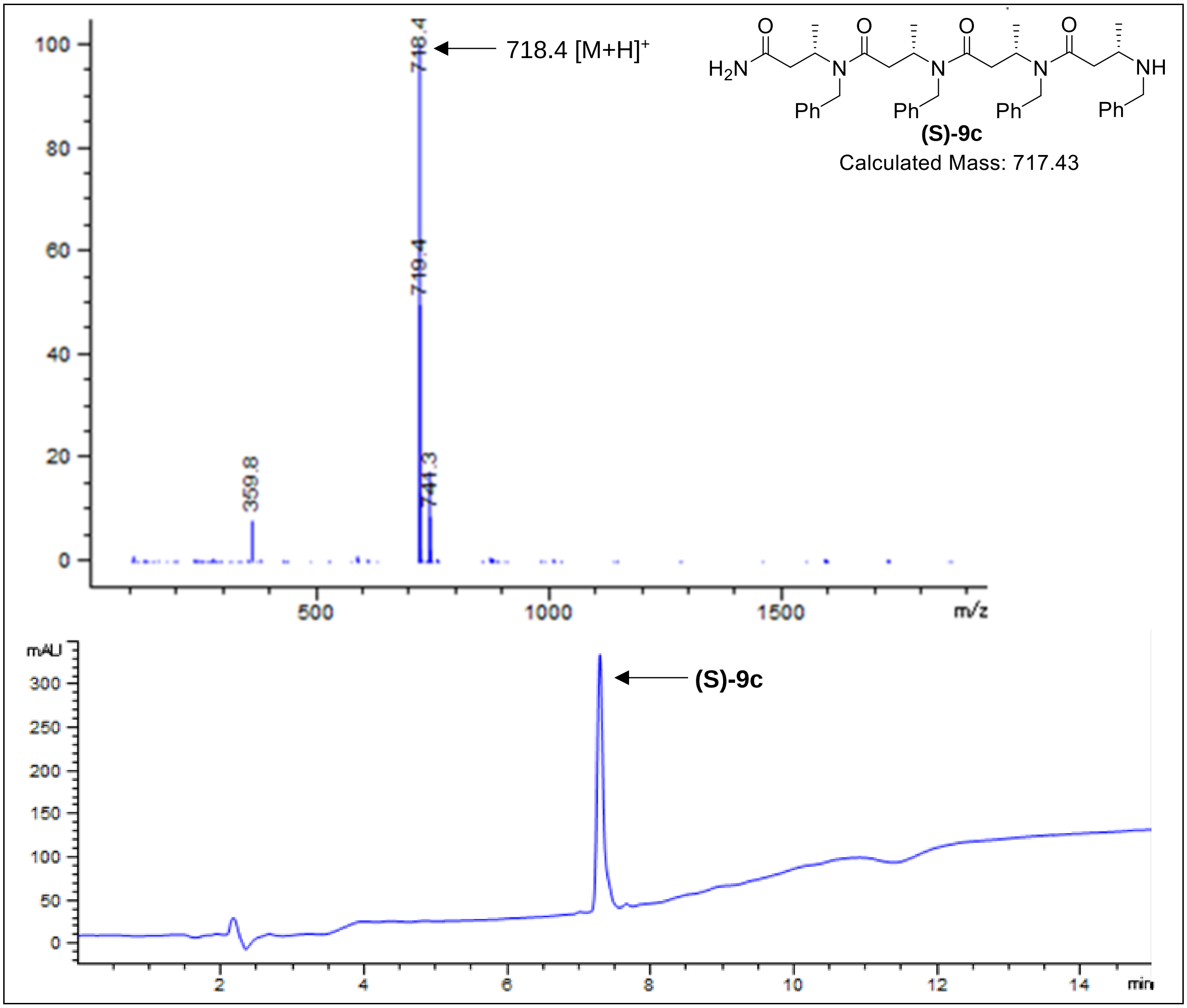

Figure S4, continued

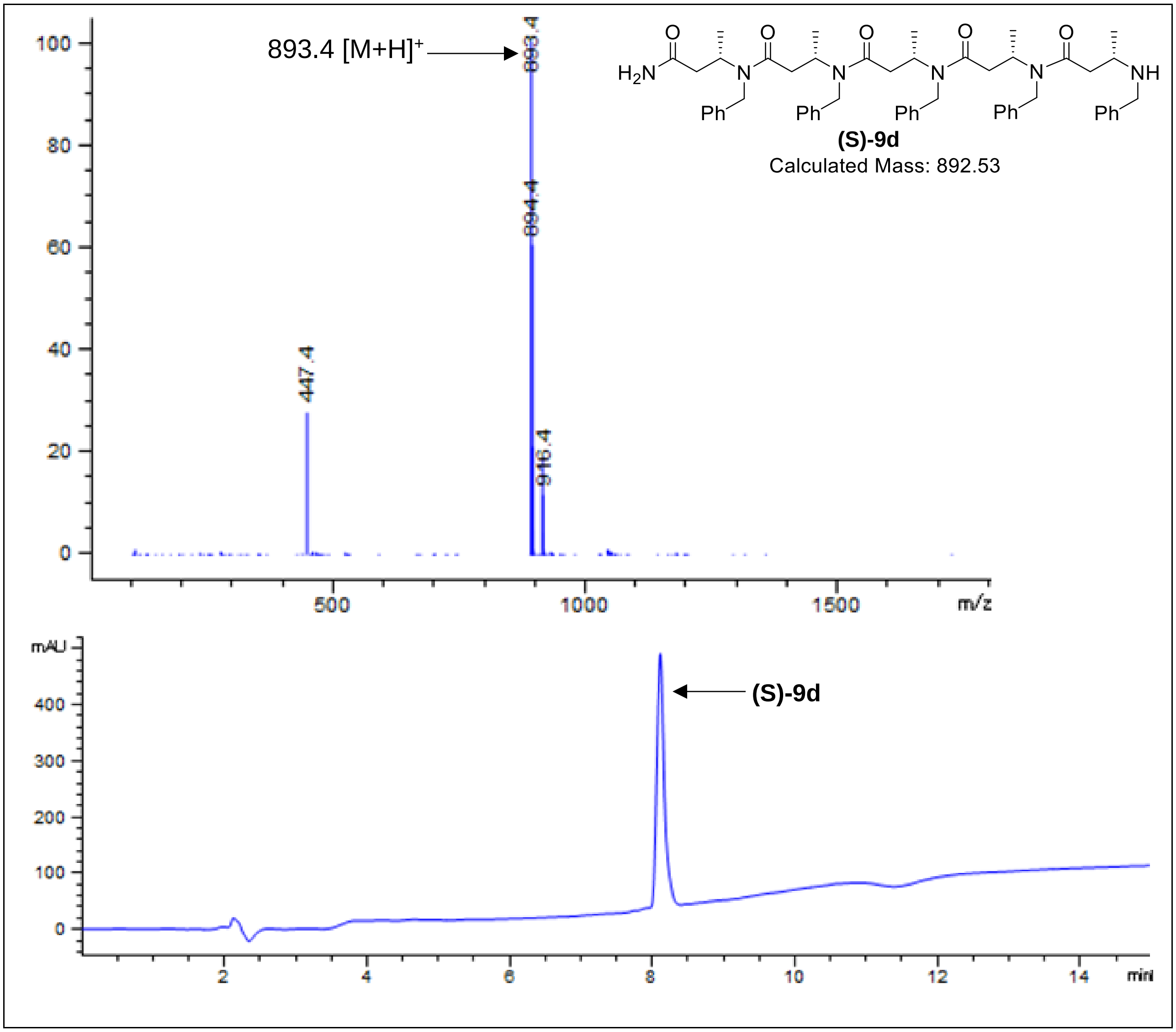

Figure S4, continued

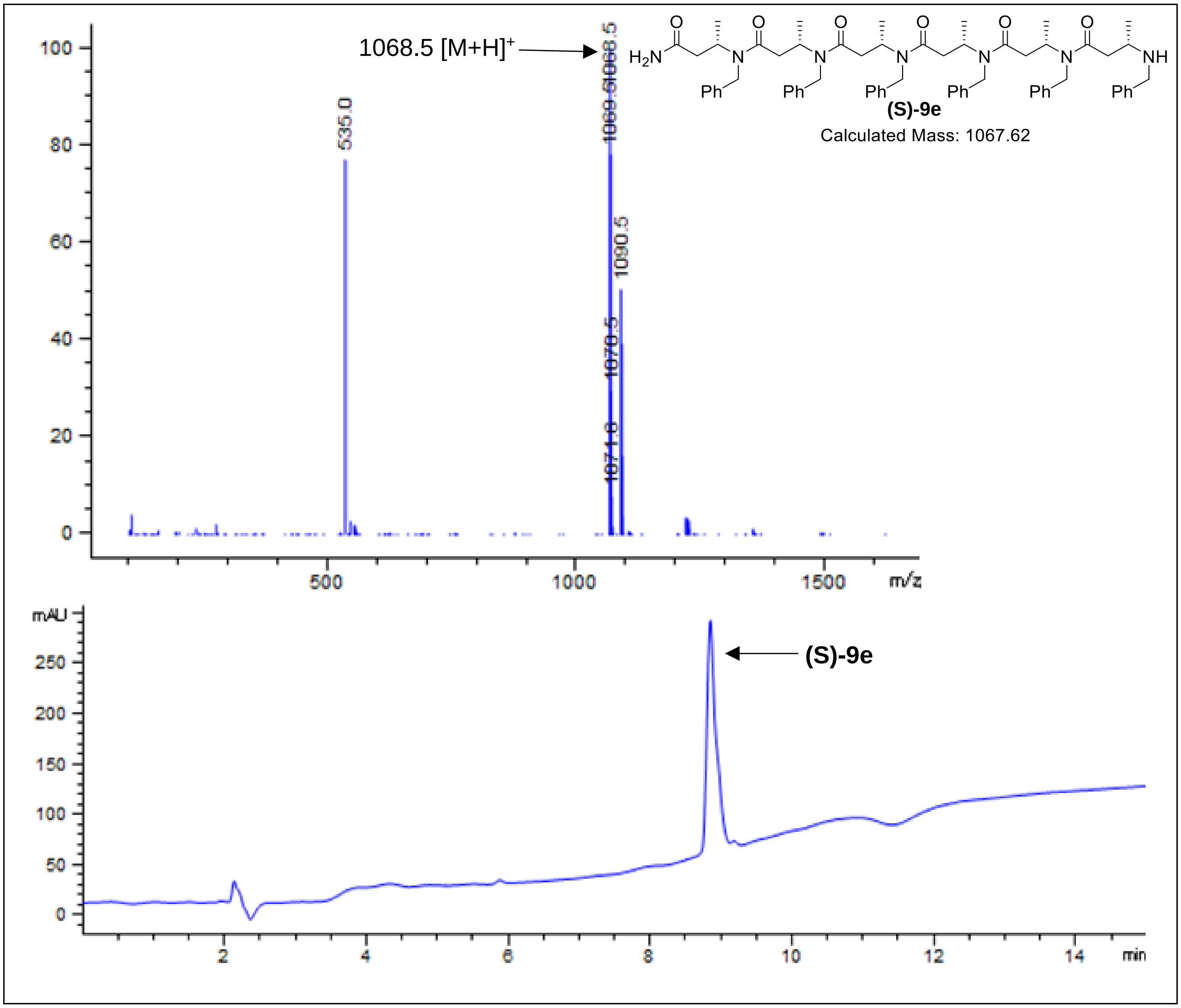

Figure S4, continued

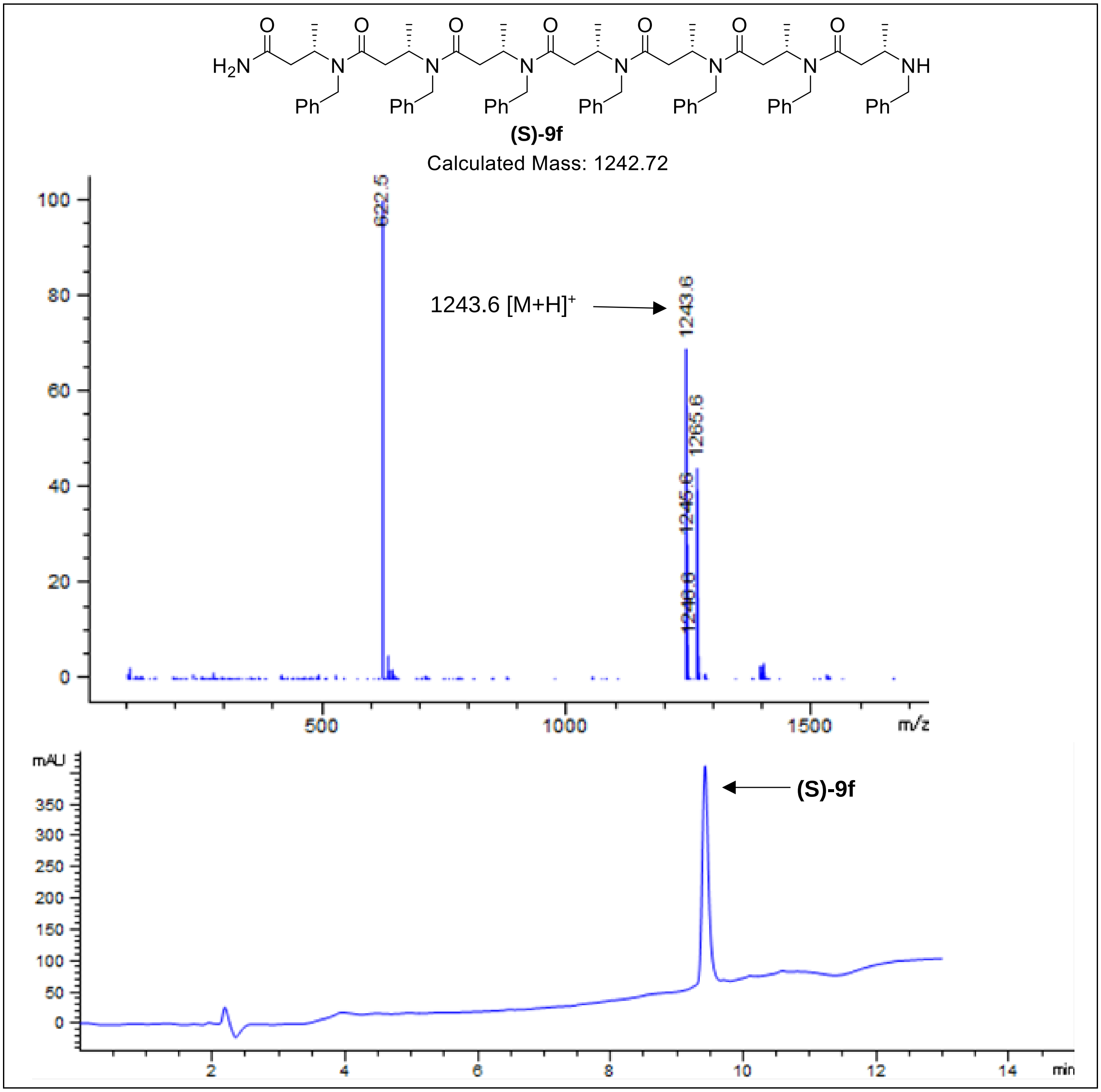

Figure S4, continued

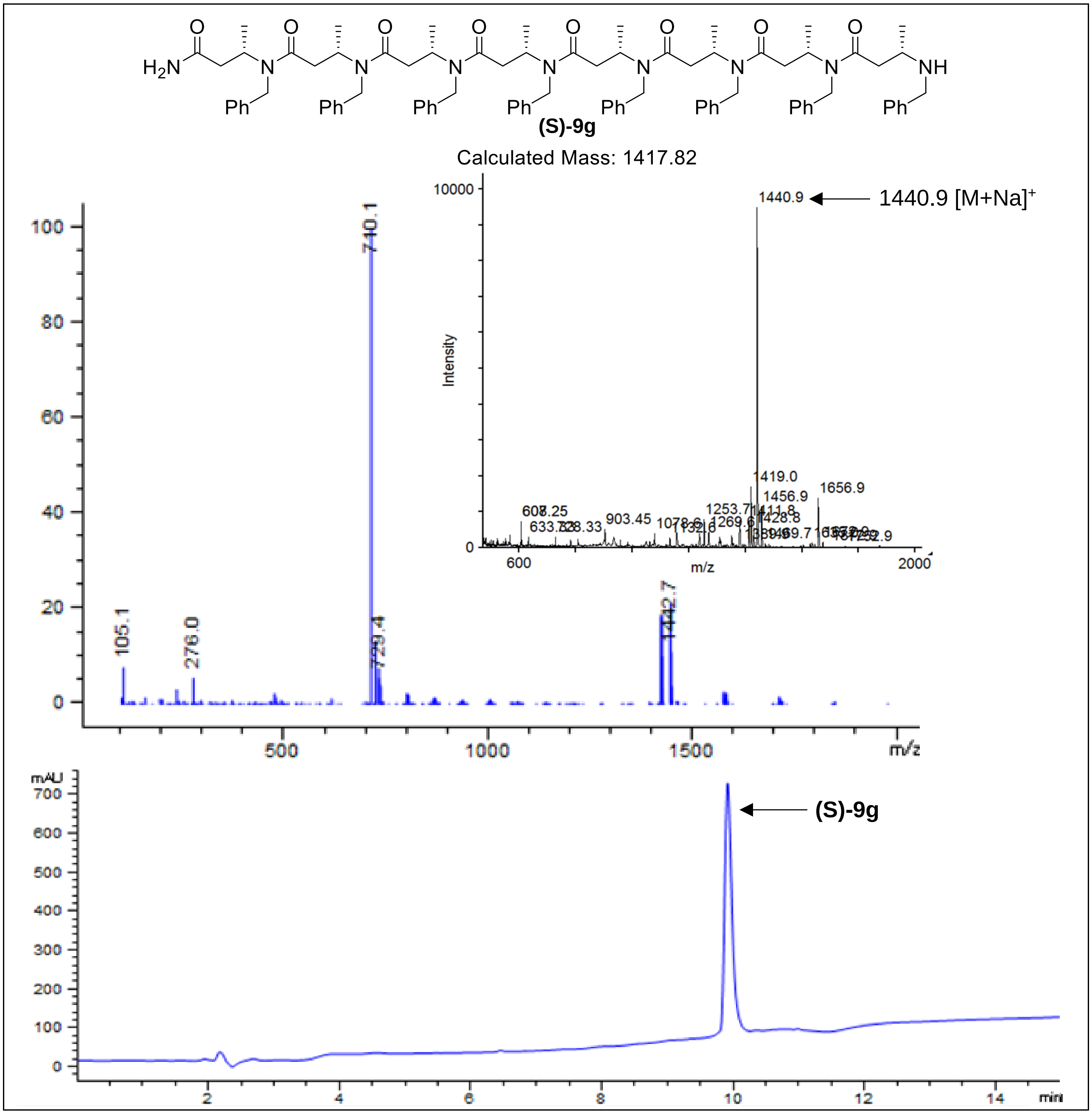

Figure S4, continued

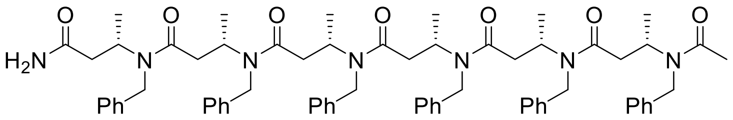

(S)-9e-Ac

Calculated Mass: 1109.64

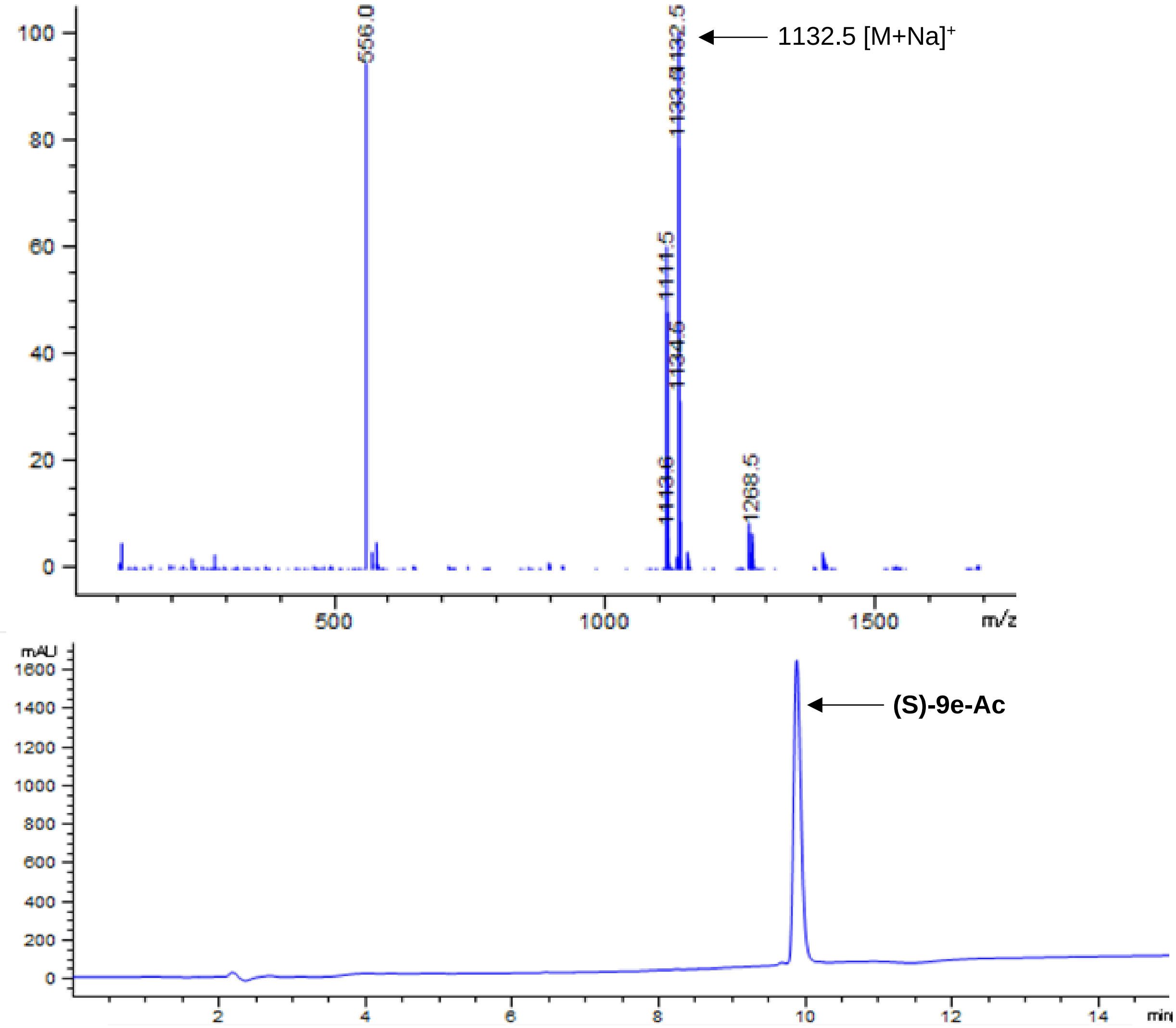

Figure S4, continued

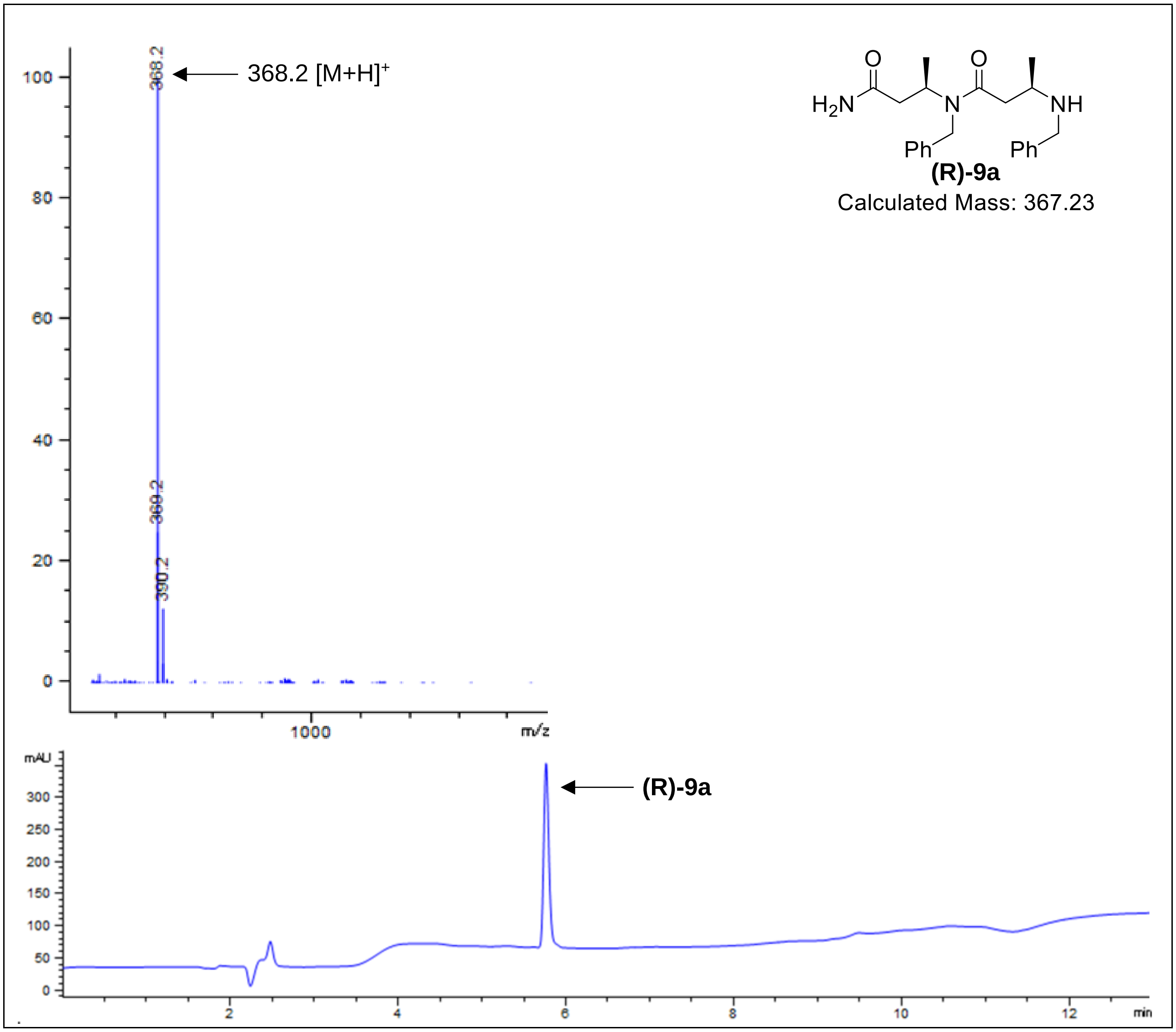

Figure S4, continued

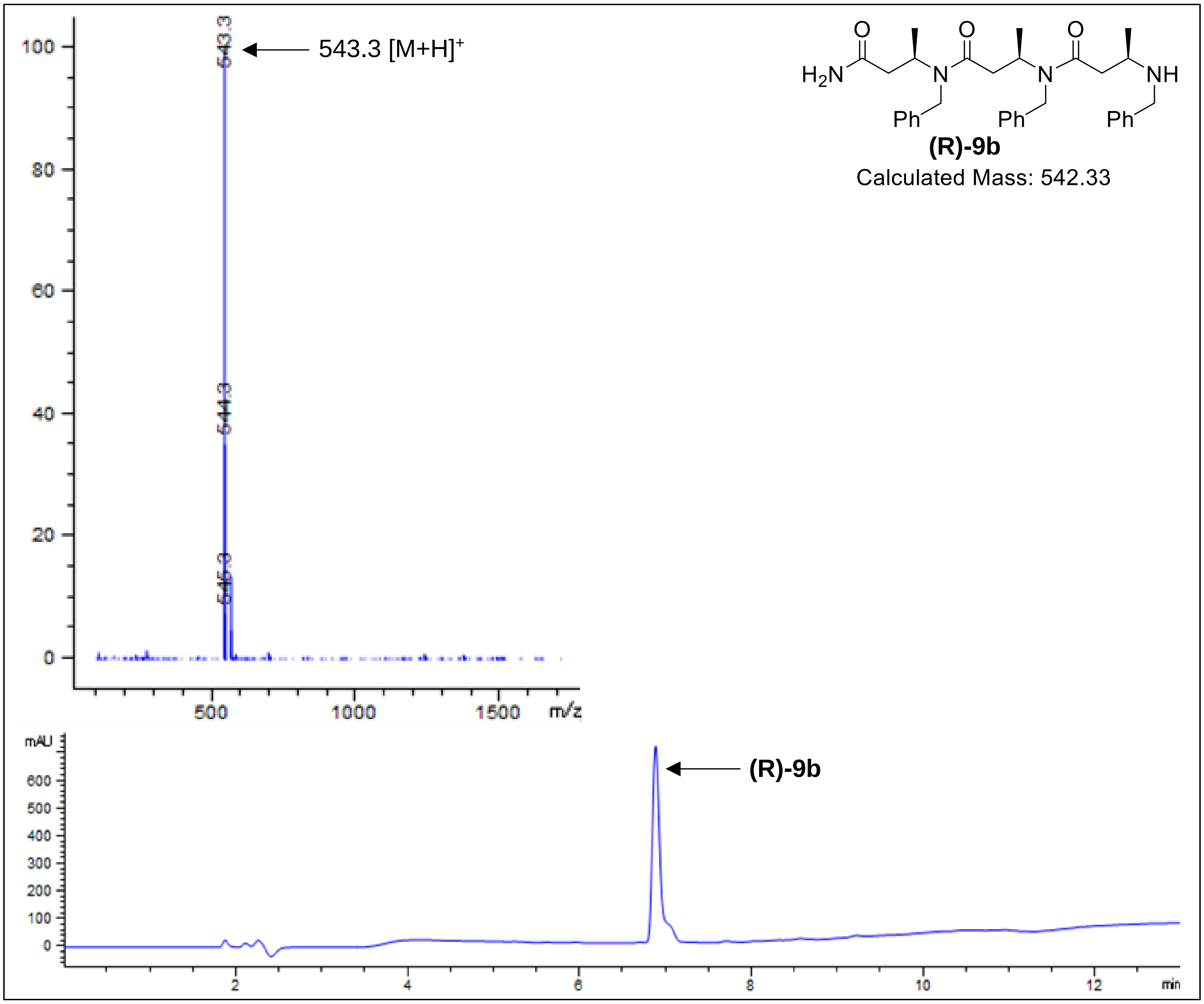

Figure S4, continued

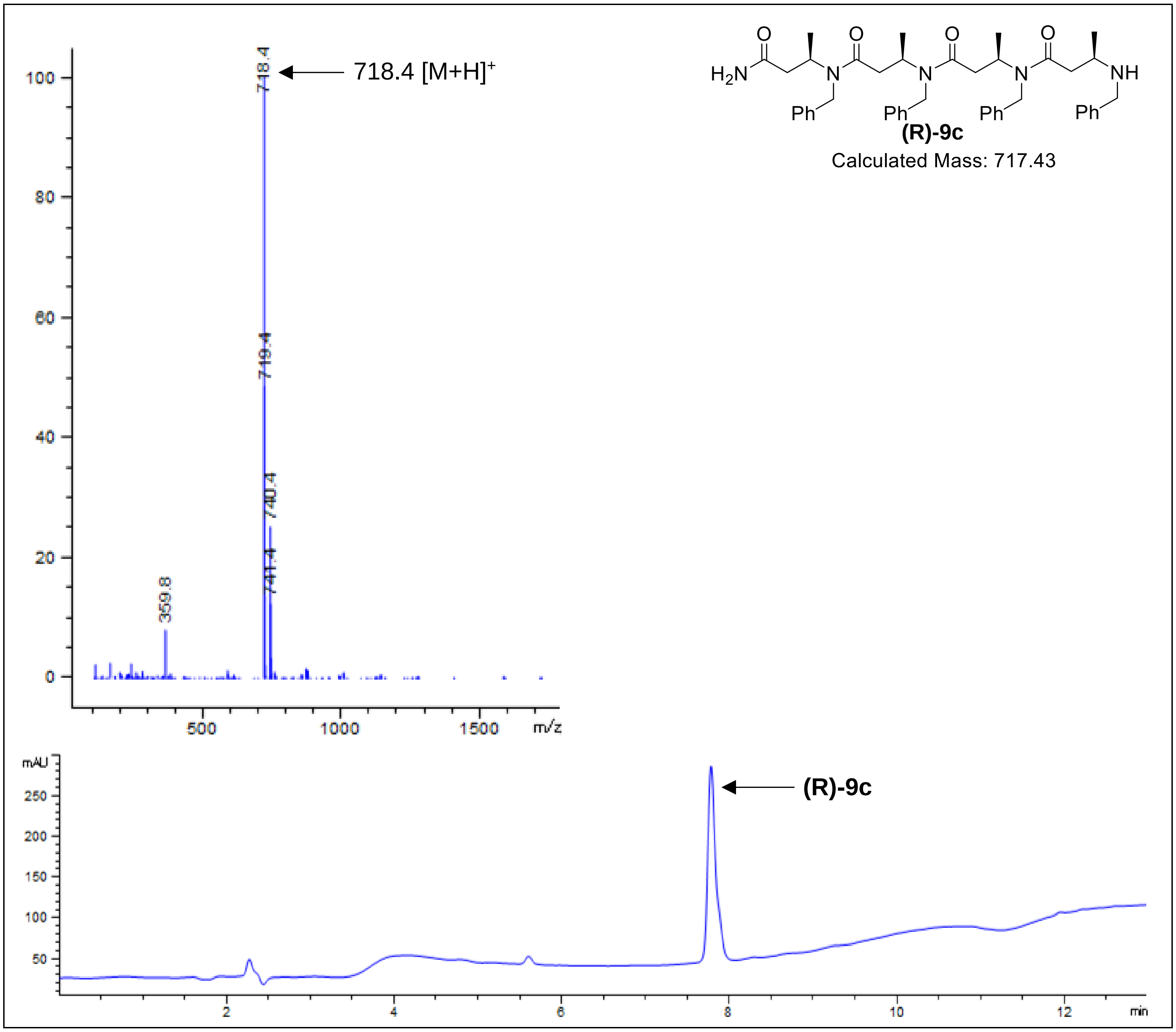

Figure S4, continued

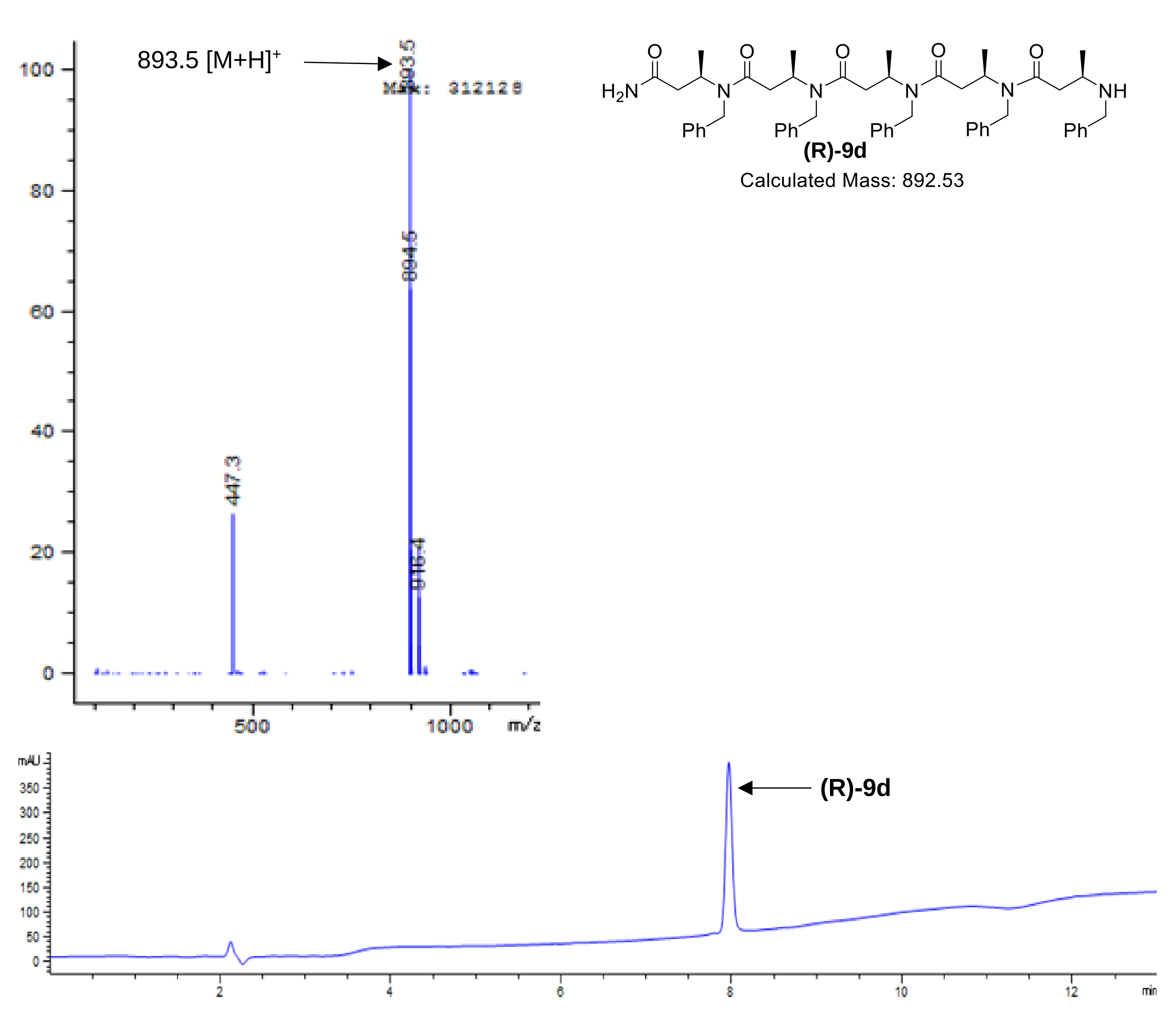

Figure S4, continued

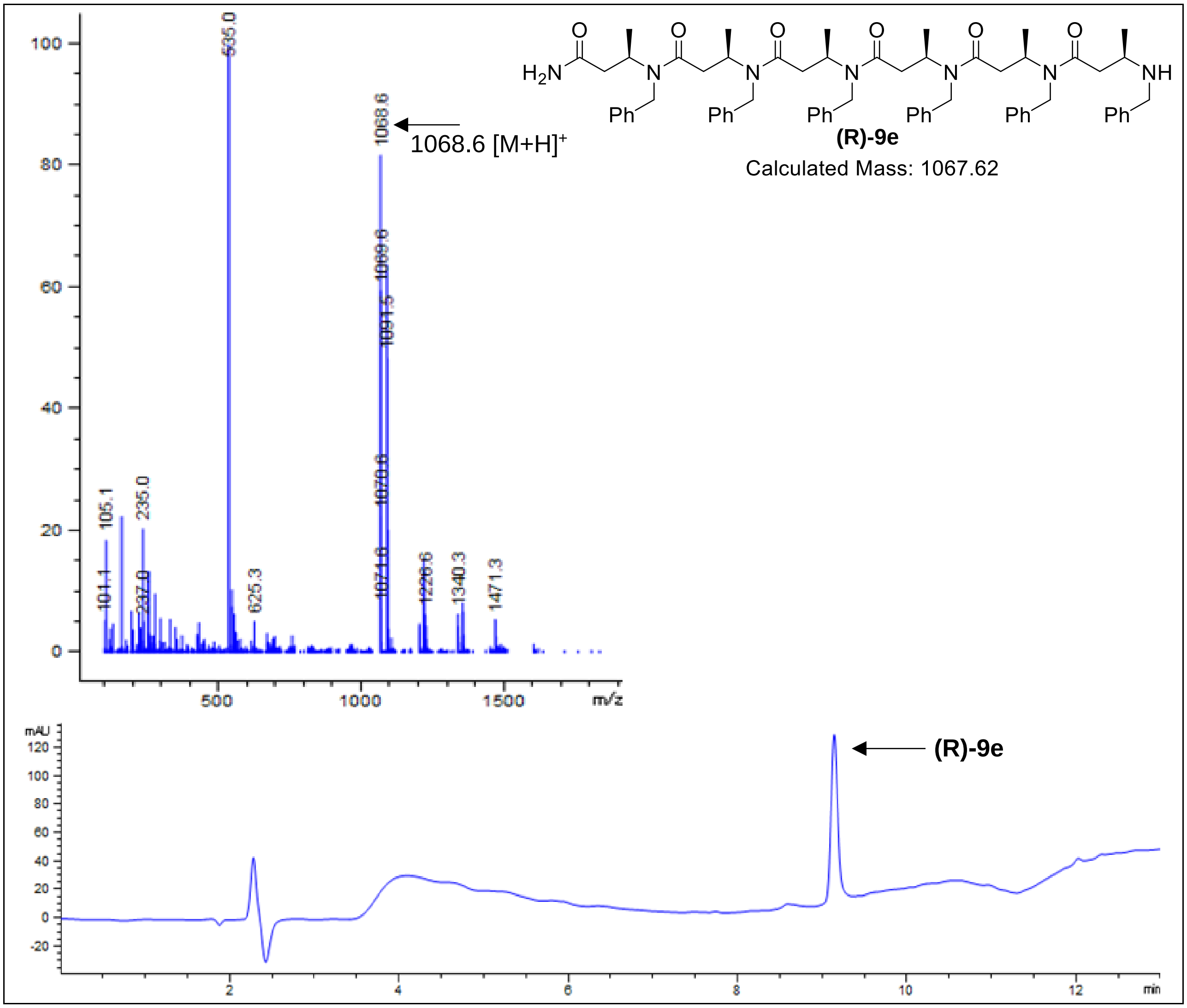

Figure S4, continued

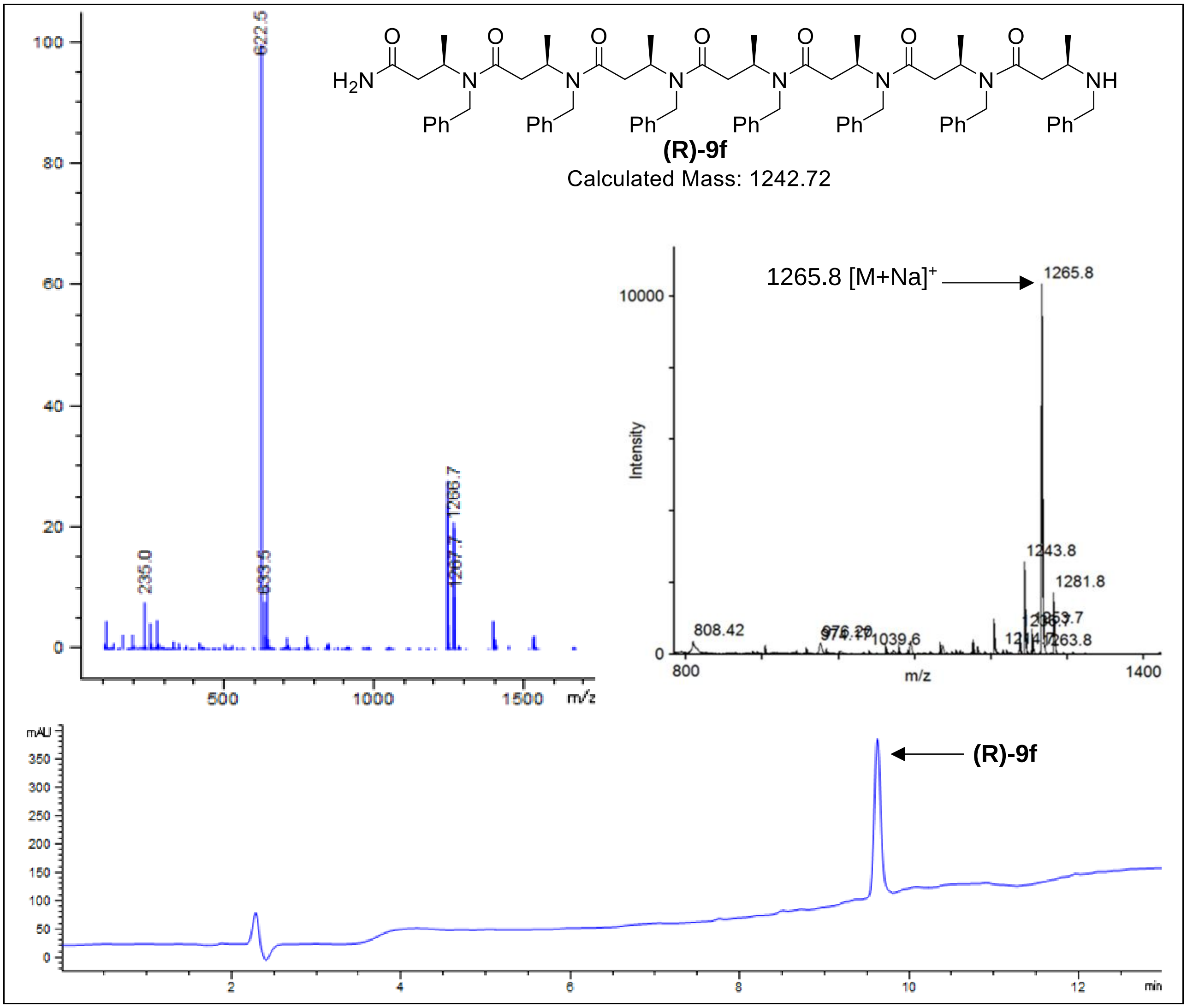

Figure S4, continued

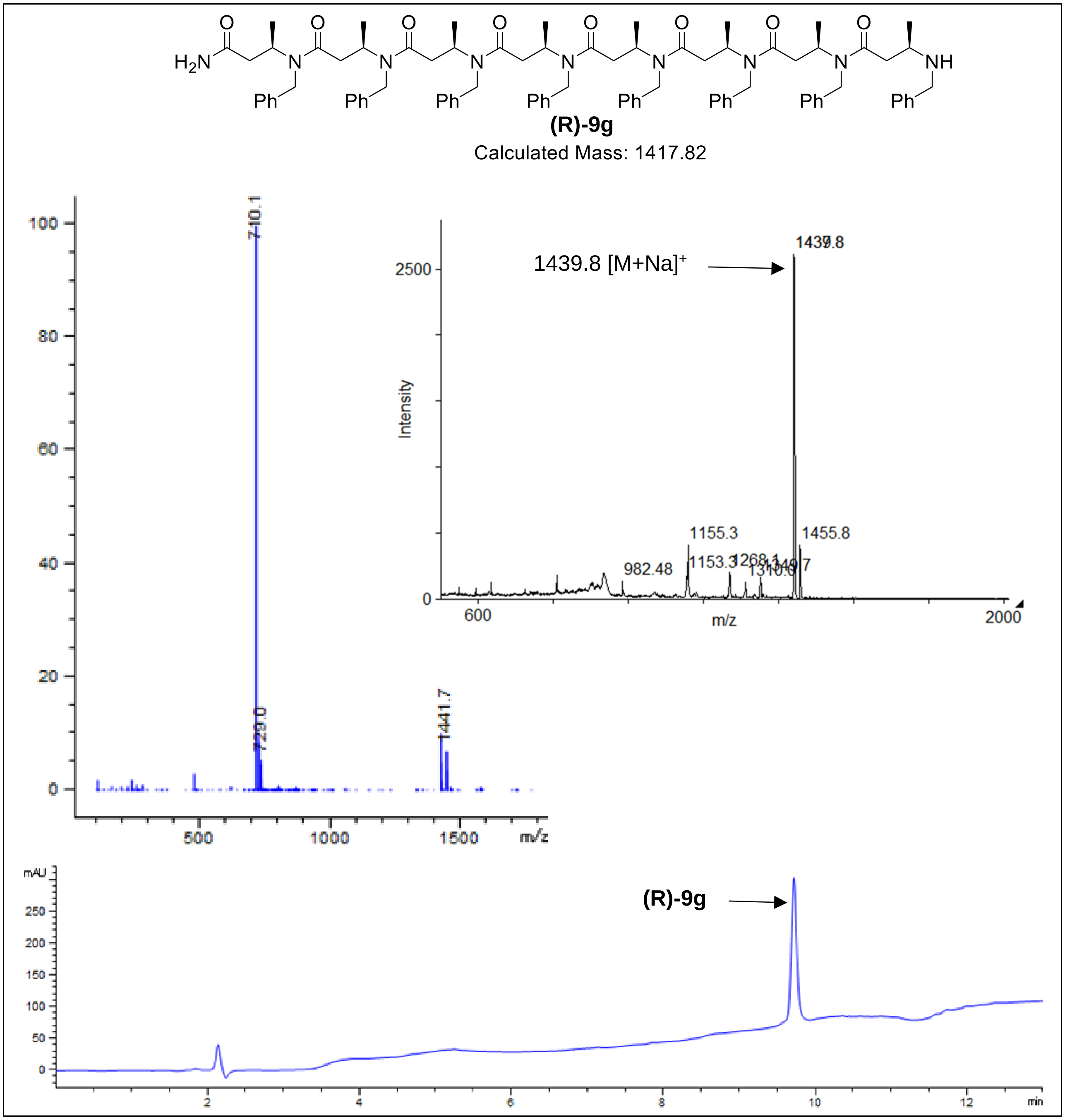

Figure S4, continued

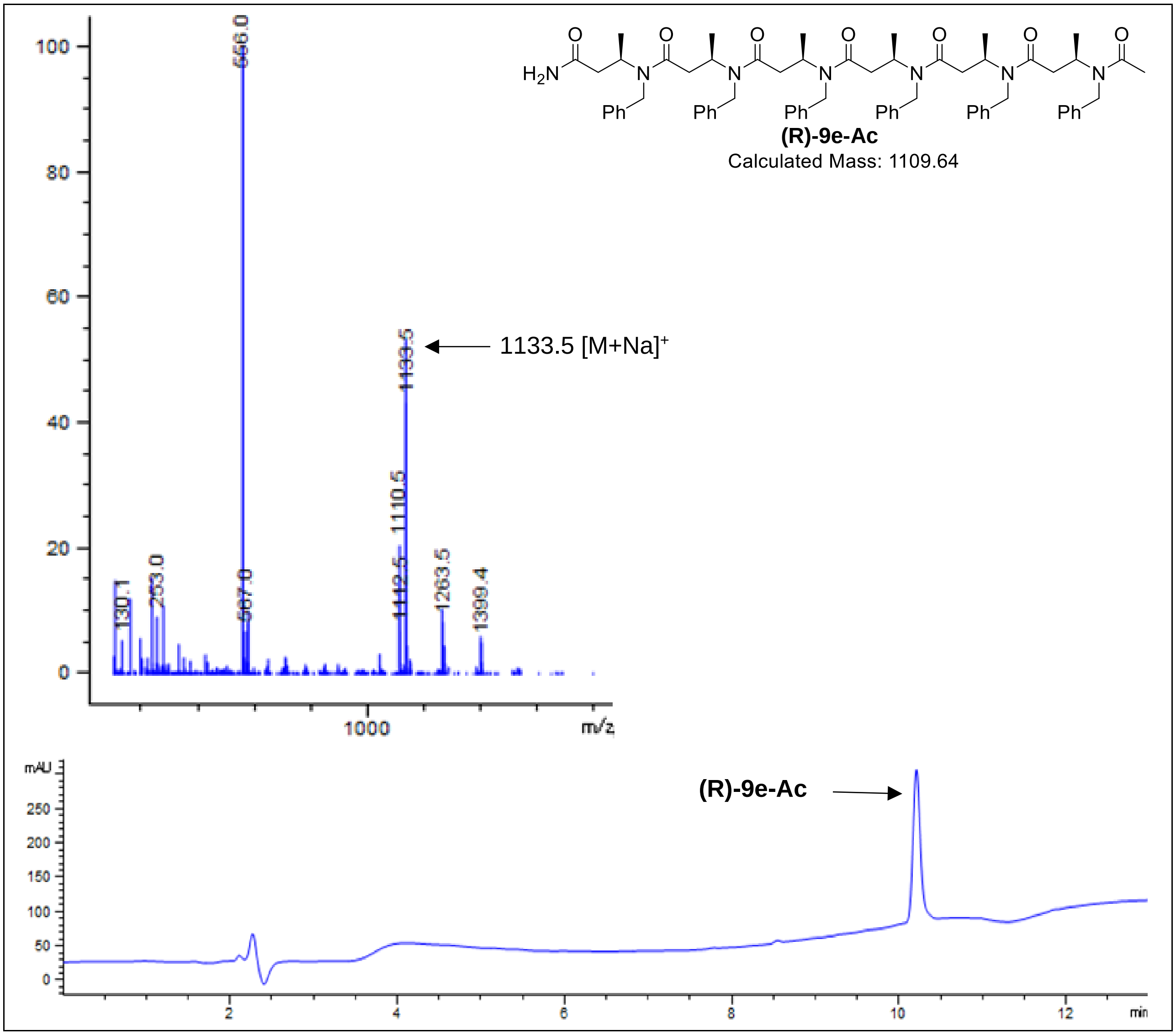

Figure S4, continued

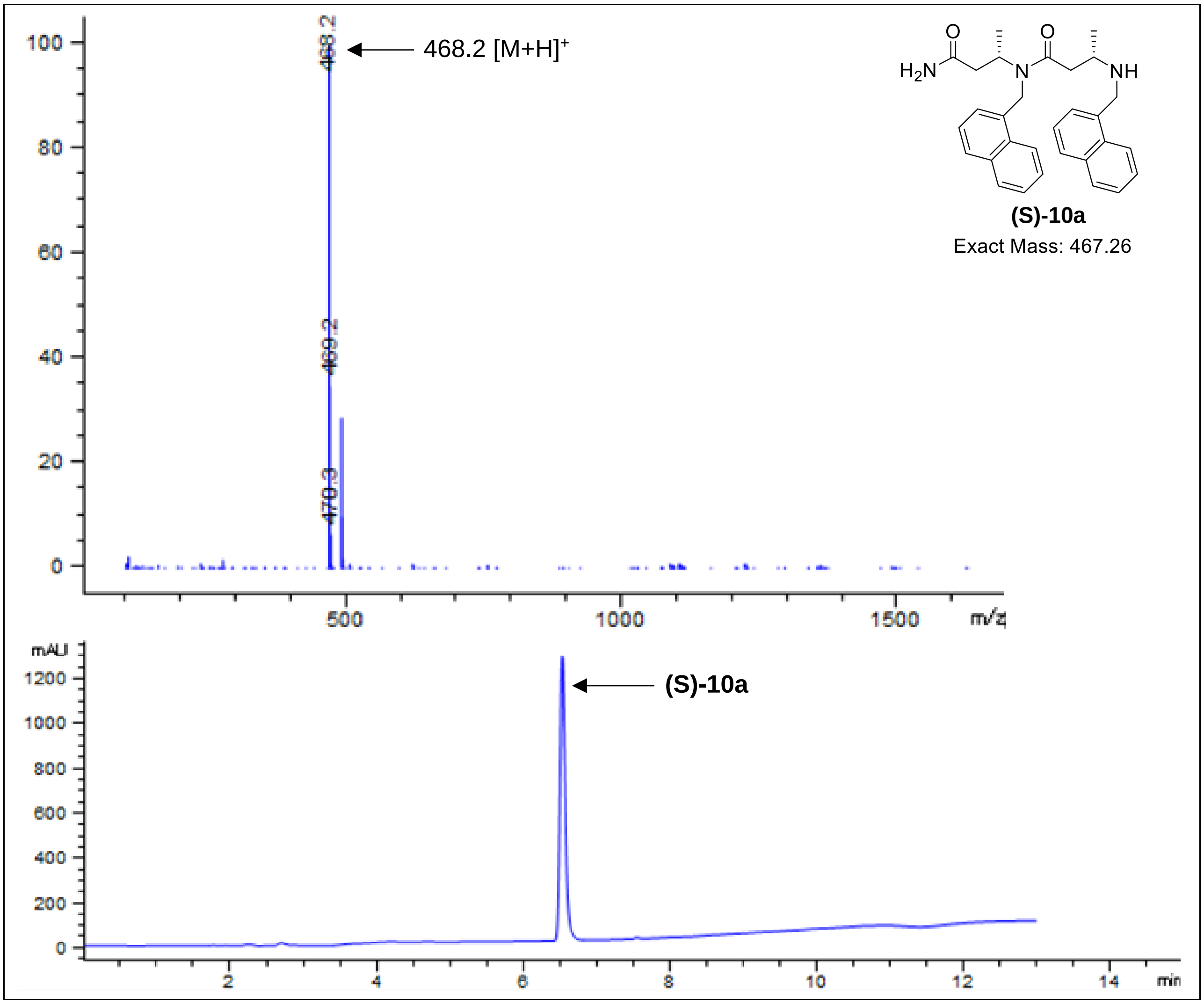

Figure S4, continued

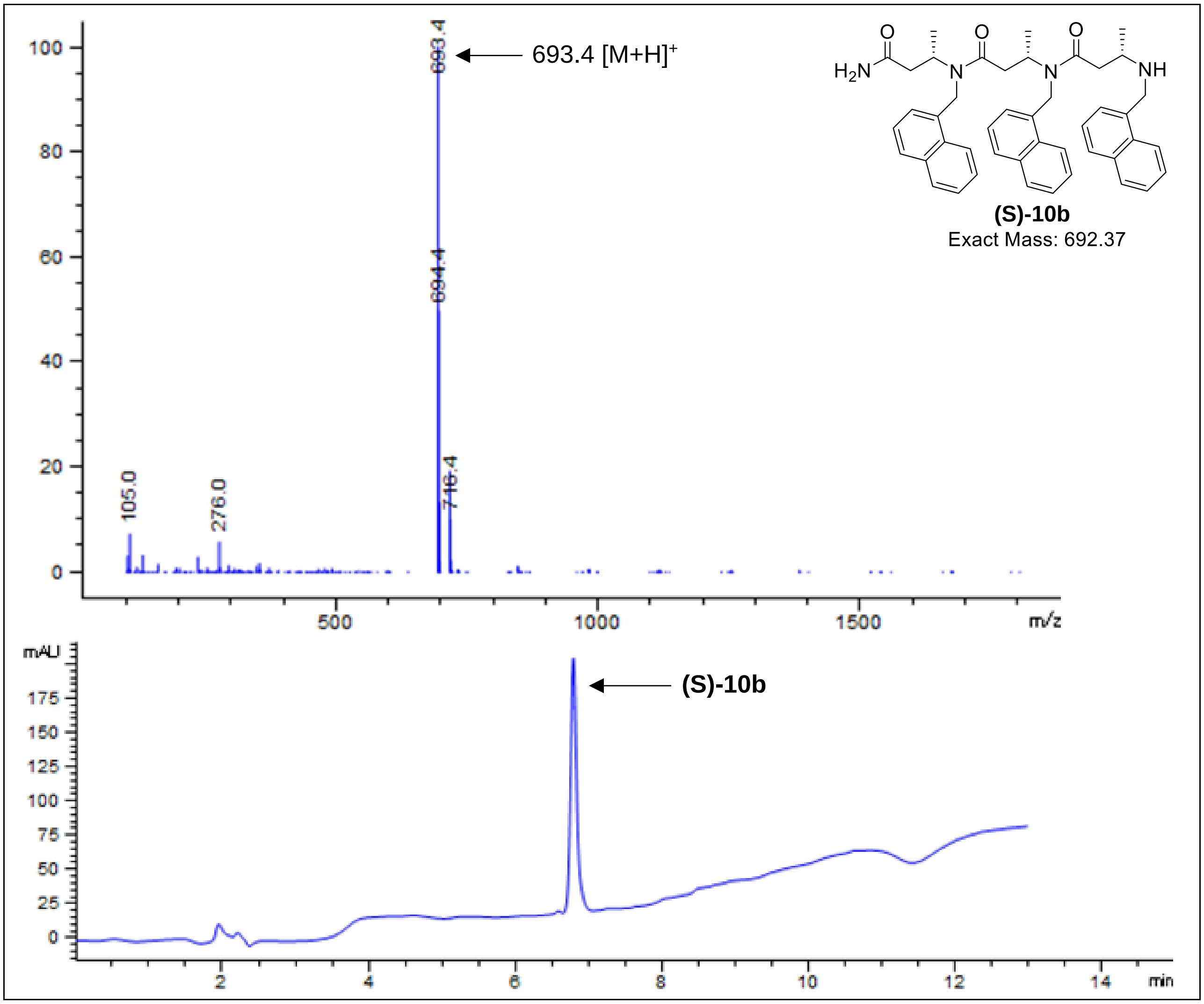

Figure S4, continued

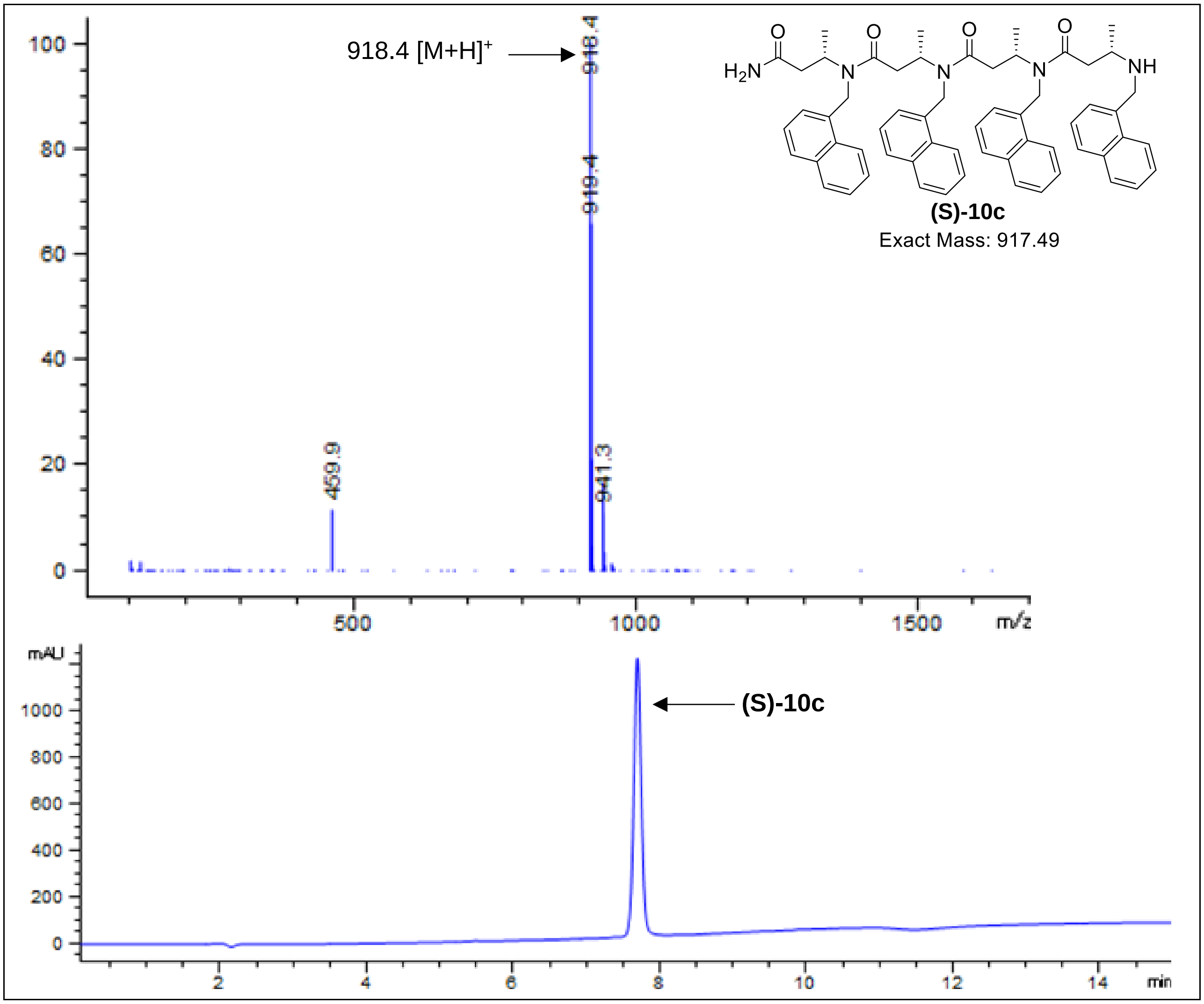

Figure S4, continued

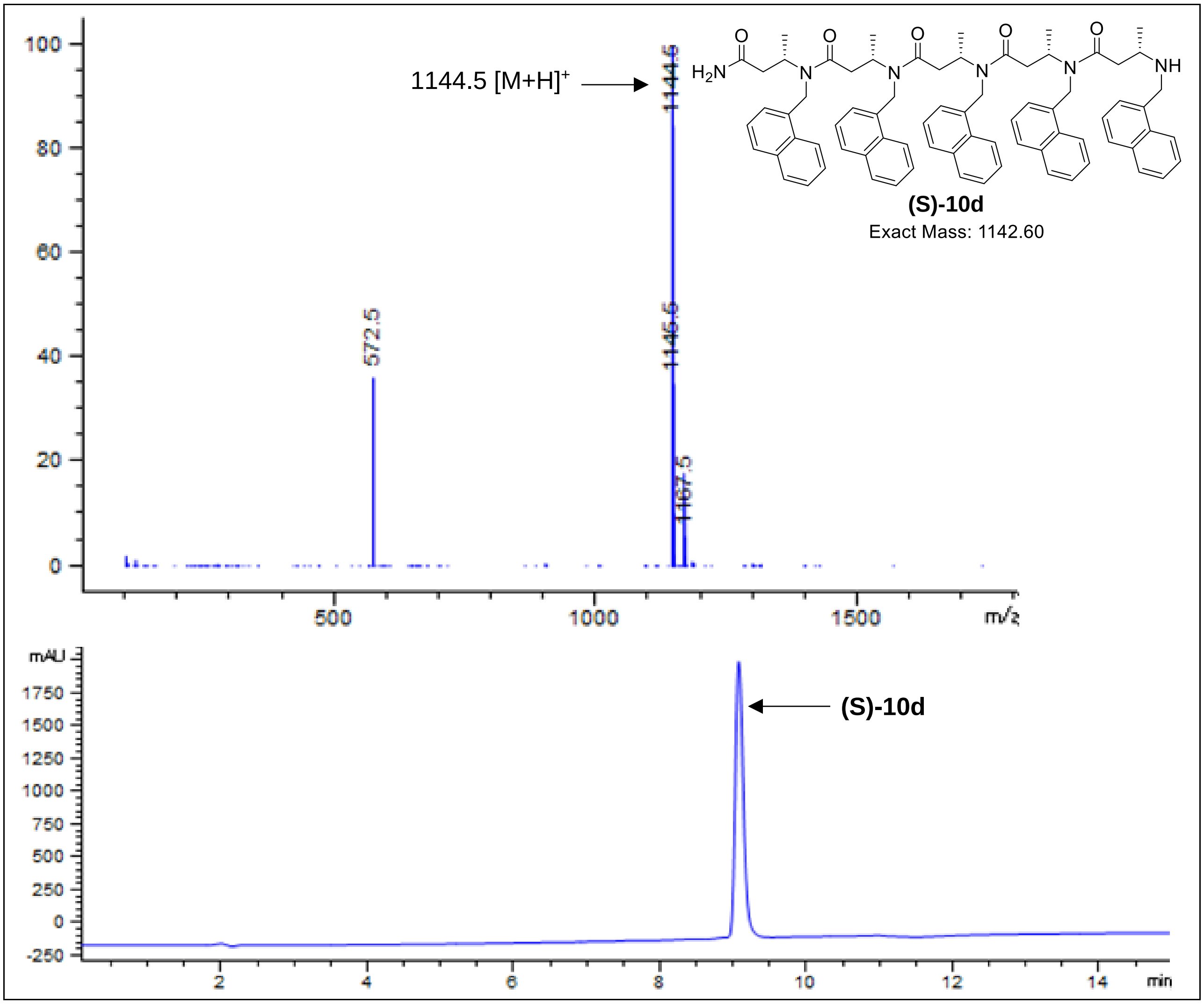

Figure S4, continued

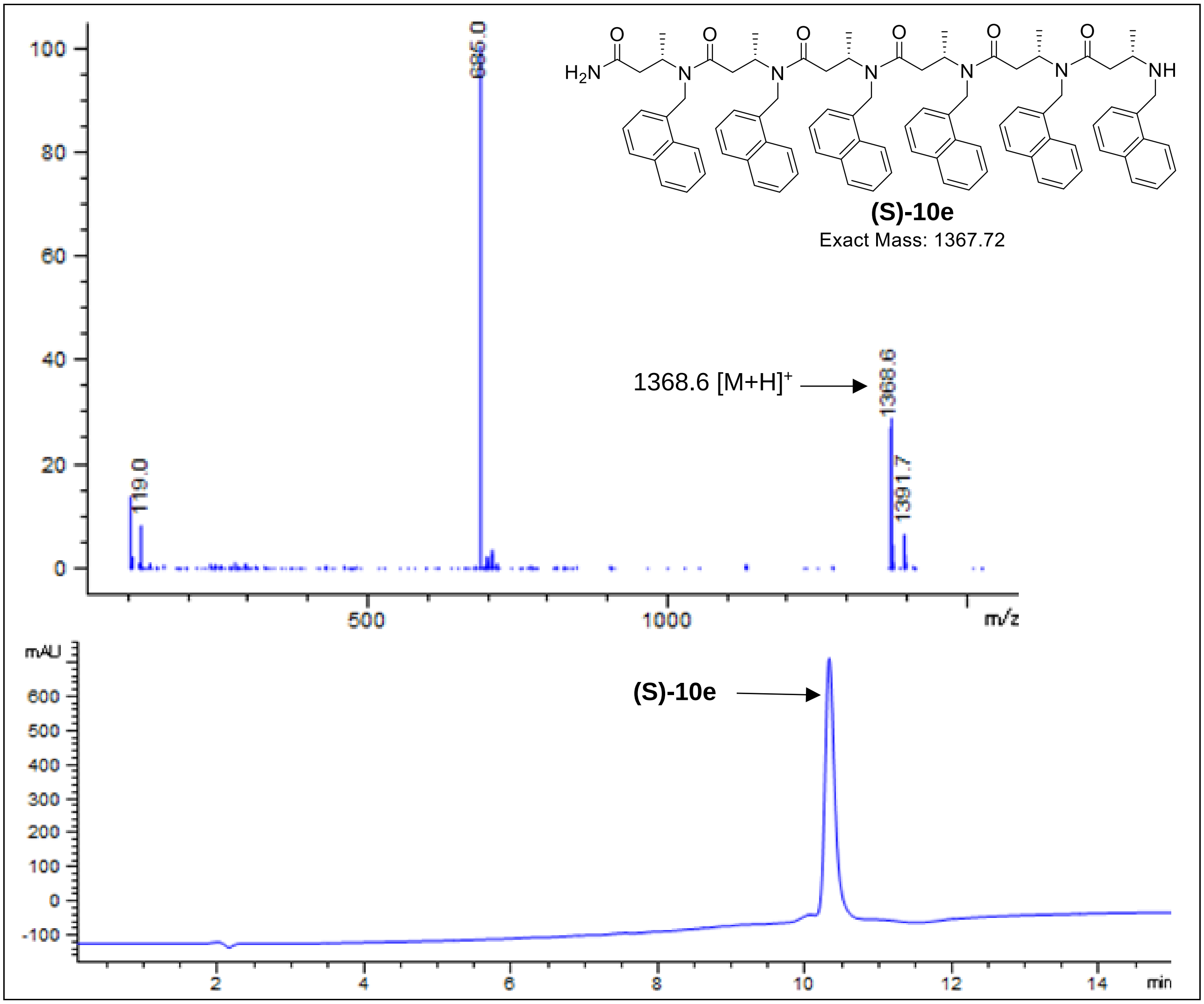

Figure S4, continued

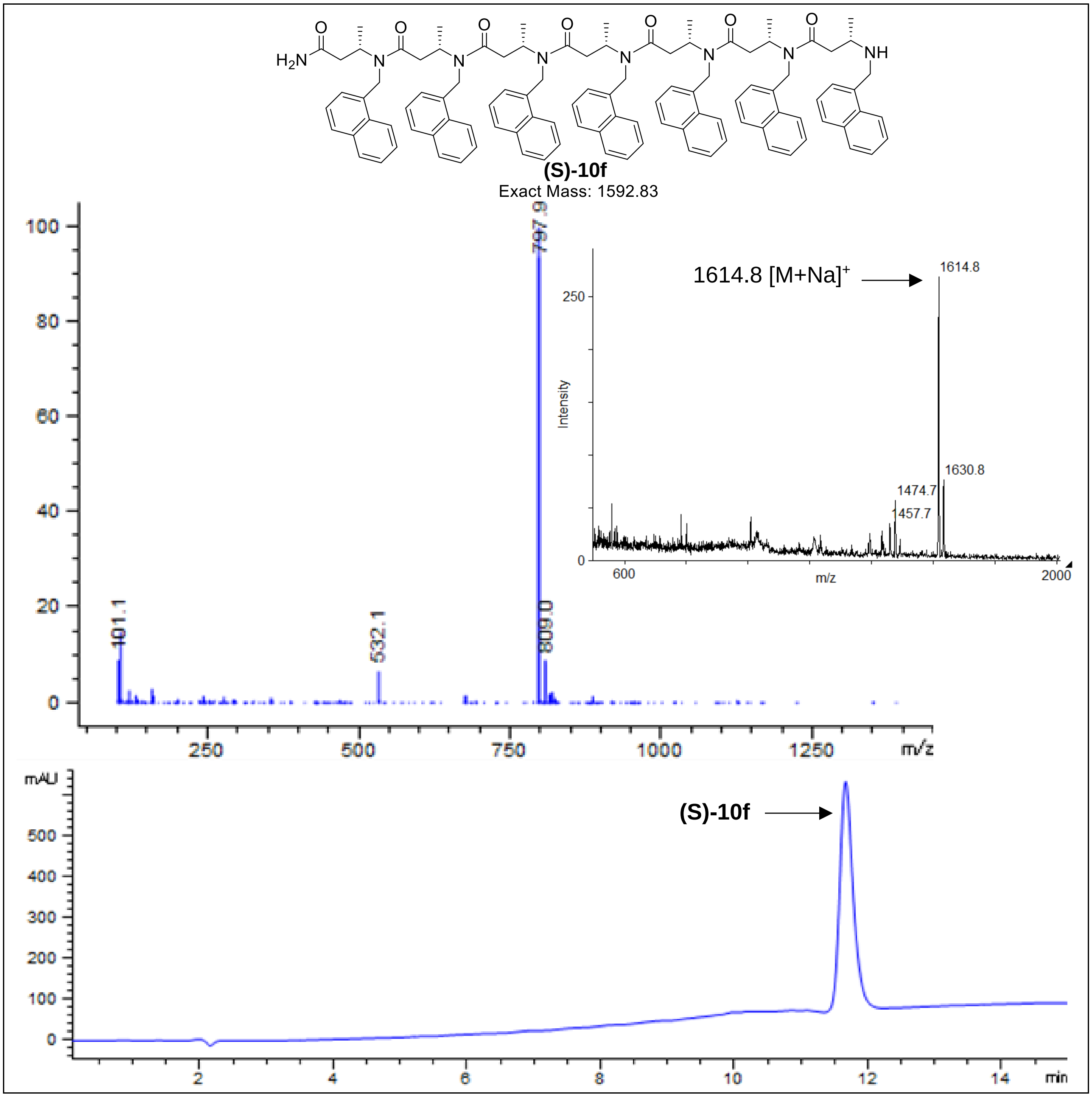

Figure S4, continued

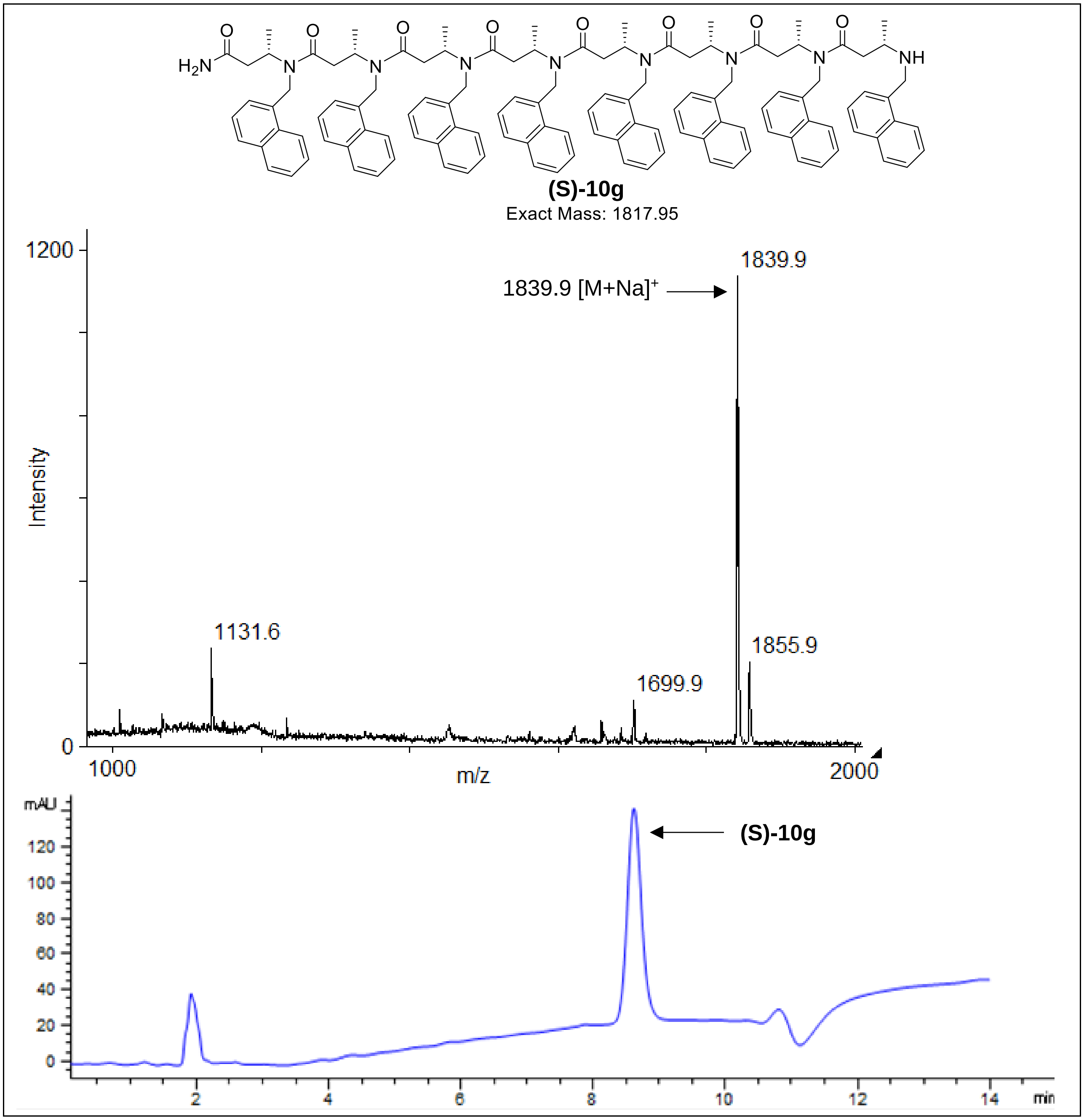

Figure S4, continued

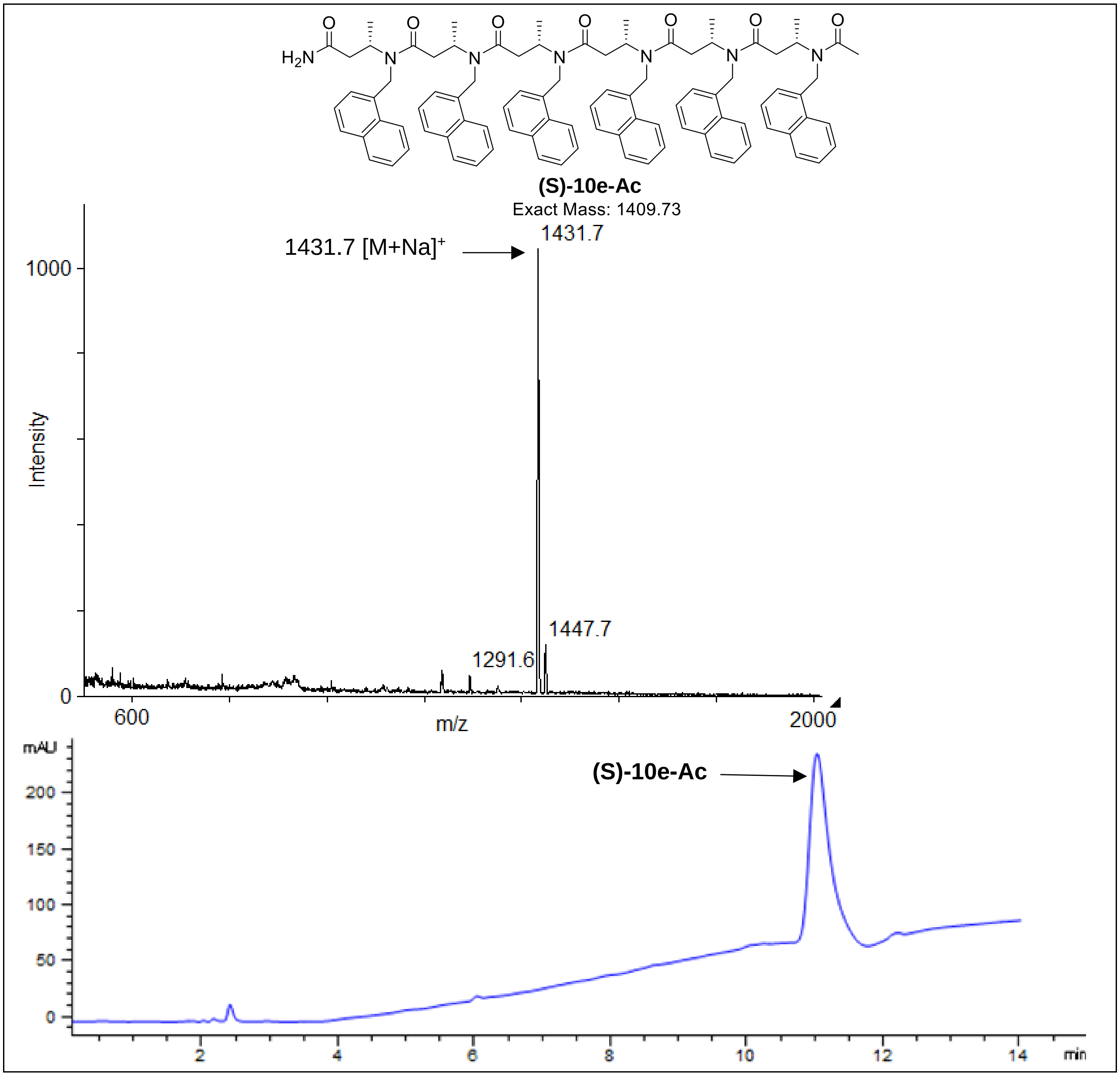

Figure S5. CD data of *N*-benzylated *β*-ABpeptoid oligomers in PBS-ACN (1:3) 60 μM (**a**) oligomers of (R)-form **(R)-9a-g** and, (**b**) (S)-form **(S)-9a-g**.

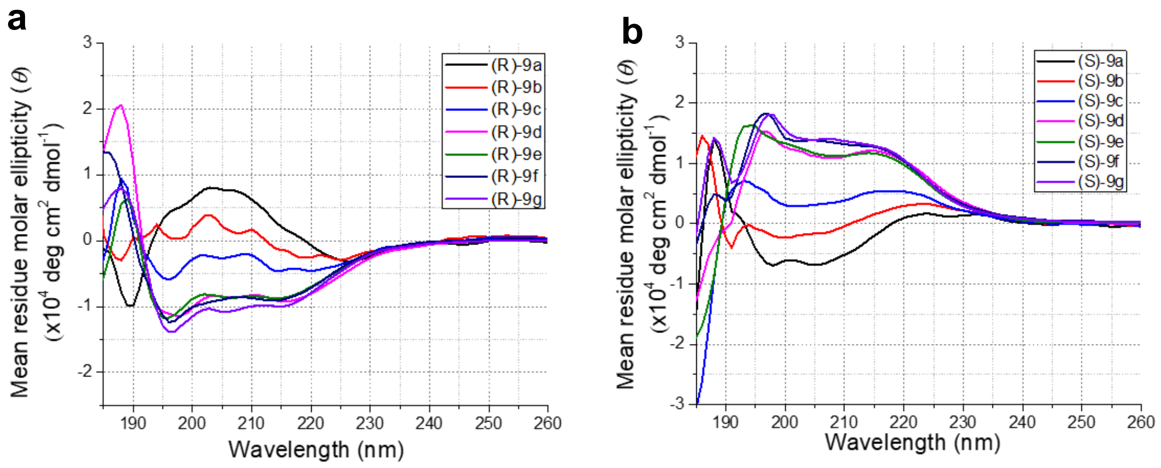

Figure S6. CD data of (S)-form of *N*-benzylated *β*-ABpeptoid oligomers (**S**)**-9a-g** in; **(a)** MeOH (60 μM) and, **(b)** TFE (60 μM).

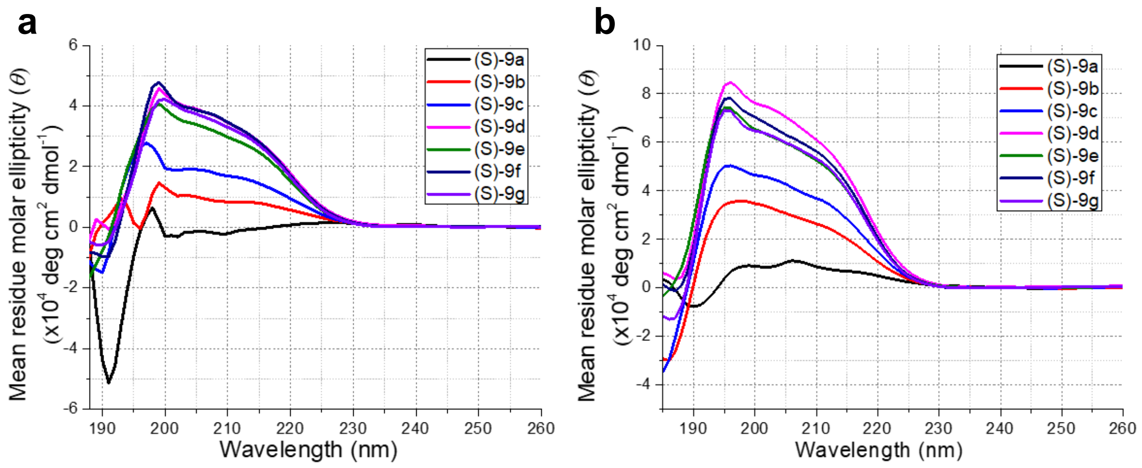

Figure S7. CD data of (S)-form of *N*-napthylmethyl *β*-ABpeptoid oligomers (**S**)**-10a-g** in; **(a)** PBS-ACN (1:3, 60 μM), **(b)** MeOH (60 μM), **(c)** TFE (60 μM).

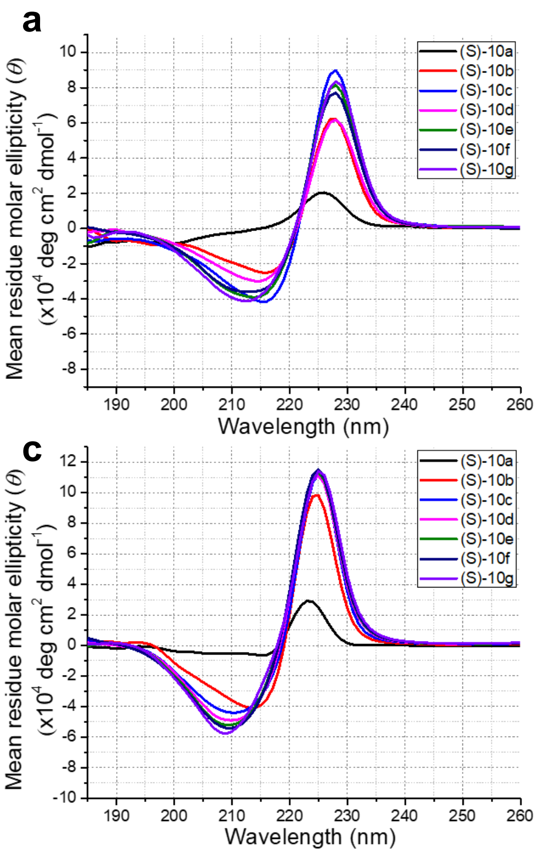

Figure S8. CD spectra (S)-form of *N*-napthylmethyl *β*-ABpeptoid octamer (**S**)**-10g** in ACN (60 μM) measured at 20 °C before and after heating to 70 °C.

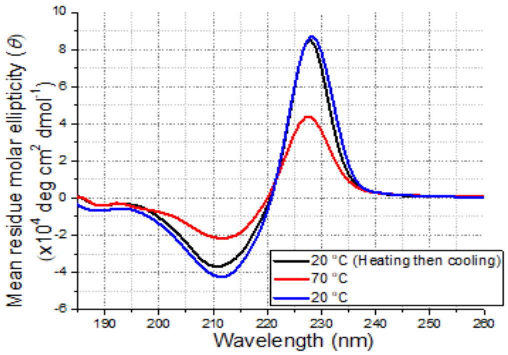

Figure S9. ^{1}H and ^{13}C NMR data of; **(R)-1**, **(S)-4**, **(S)-5a**, **(S)-5b**, **(S)-6a**, and **(S)-6b.**

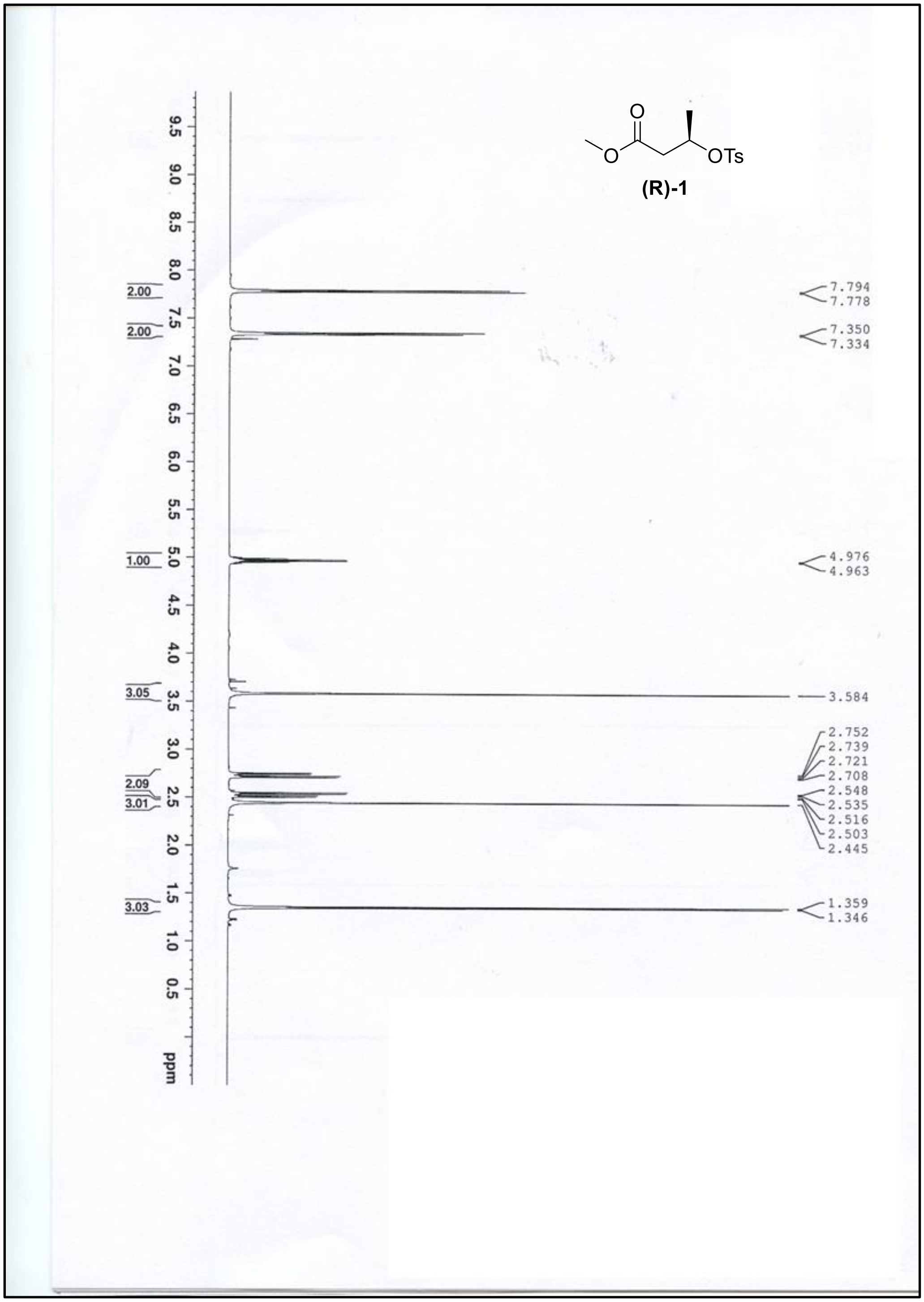

Figure S9, continued

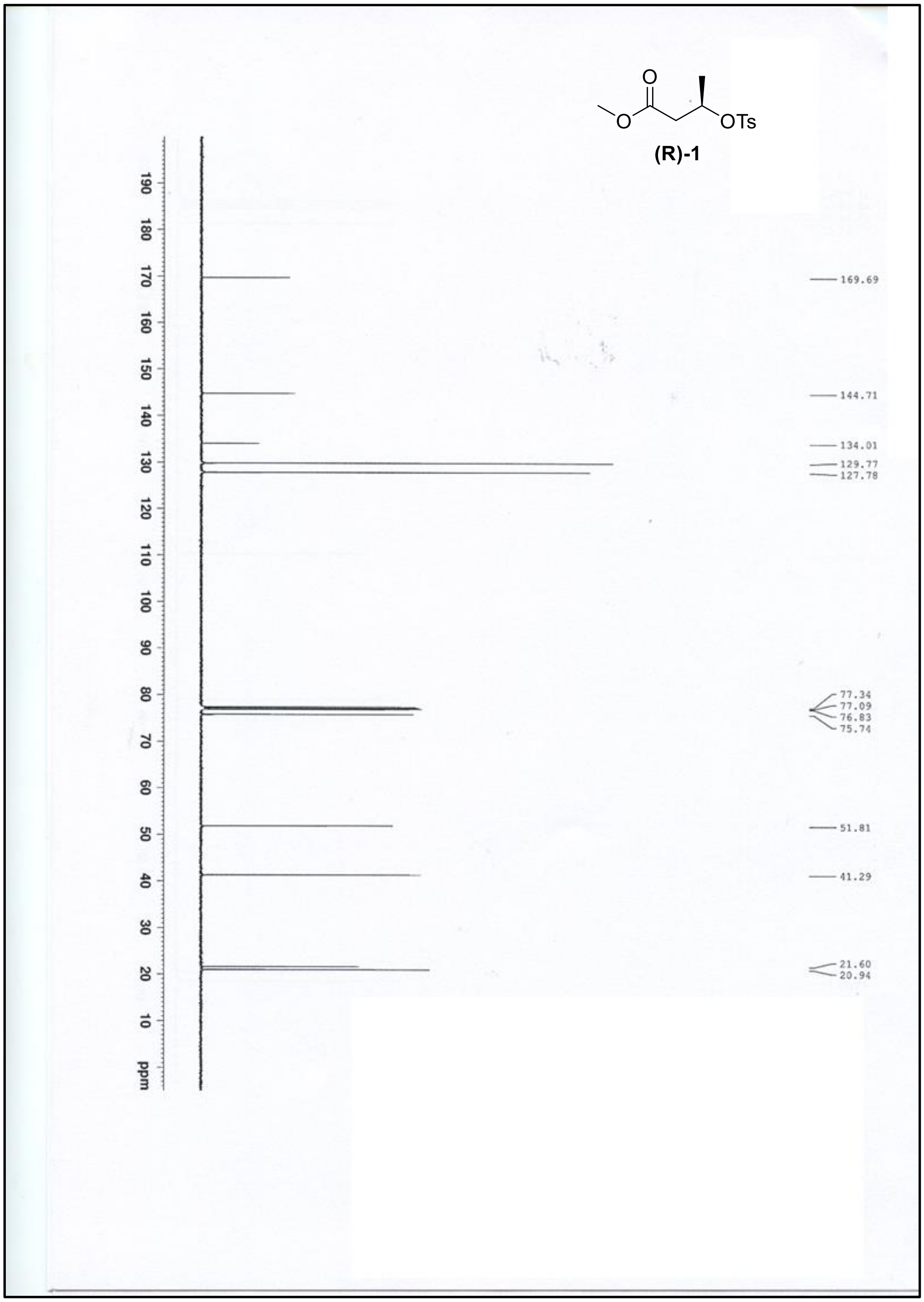

Figure S9, continued

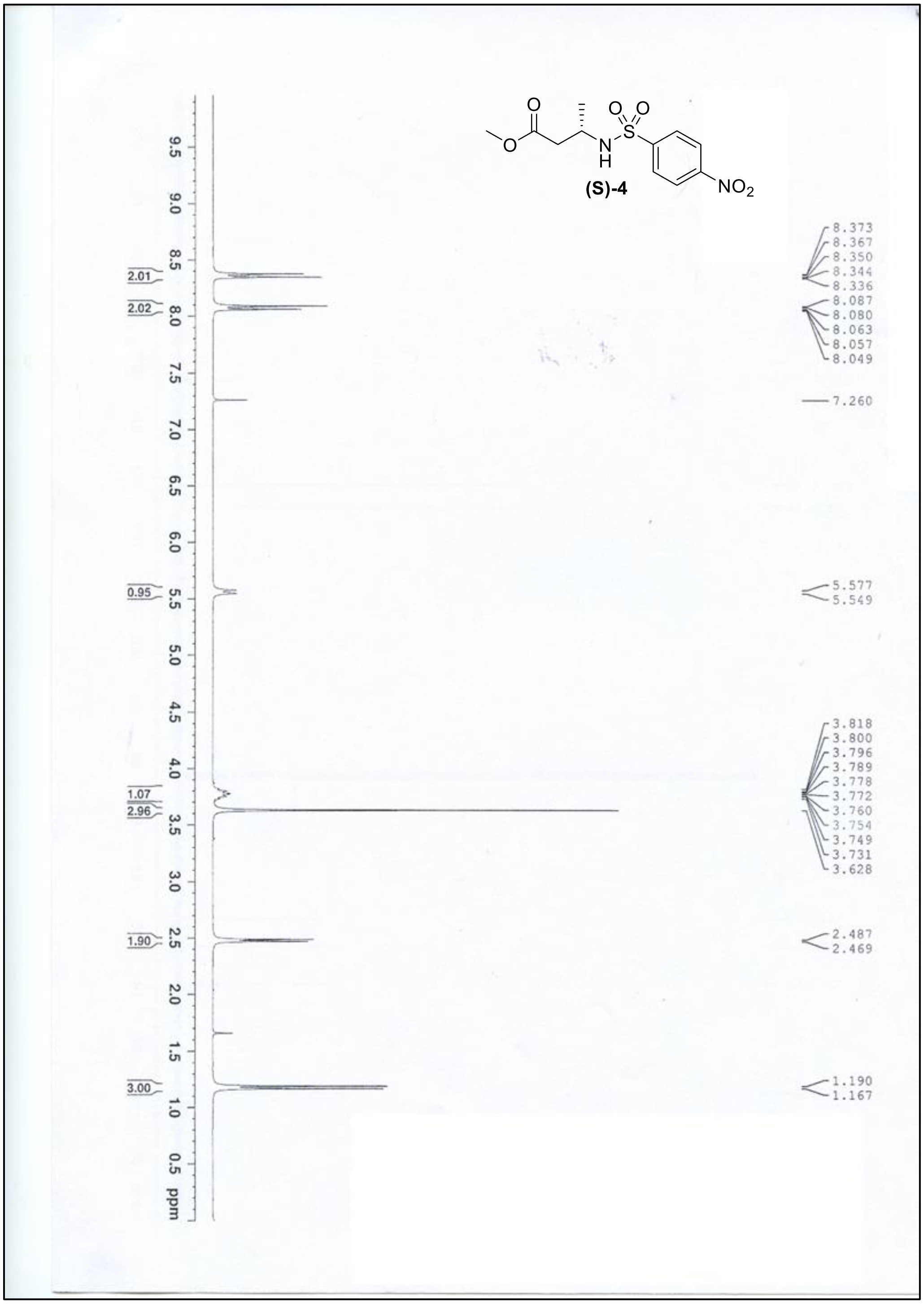

Figure S9, continued

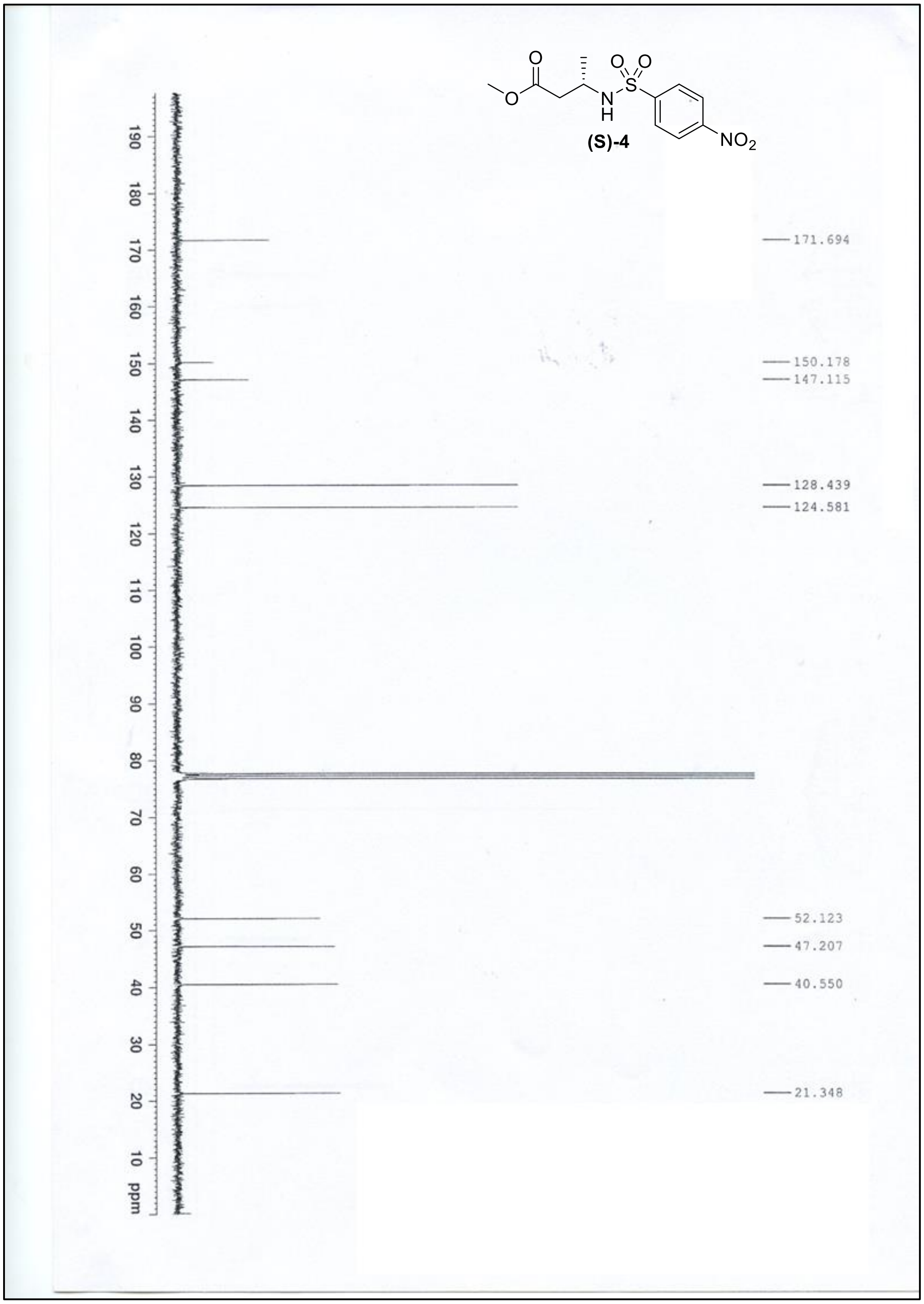

Figure S9, continued

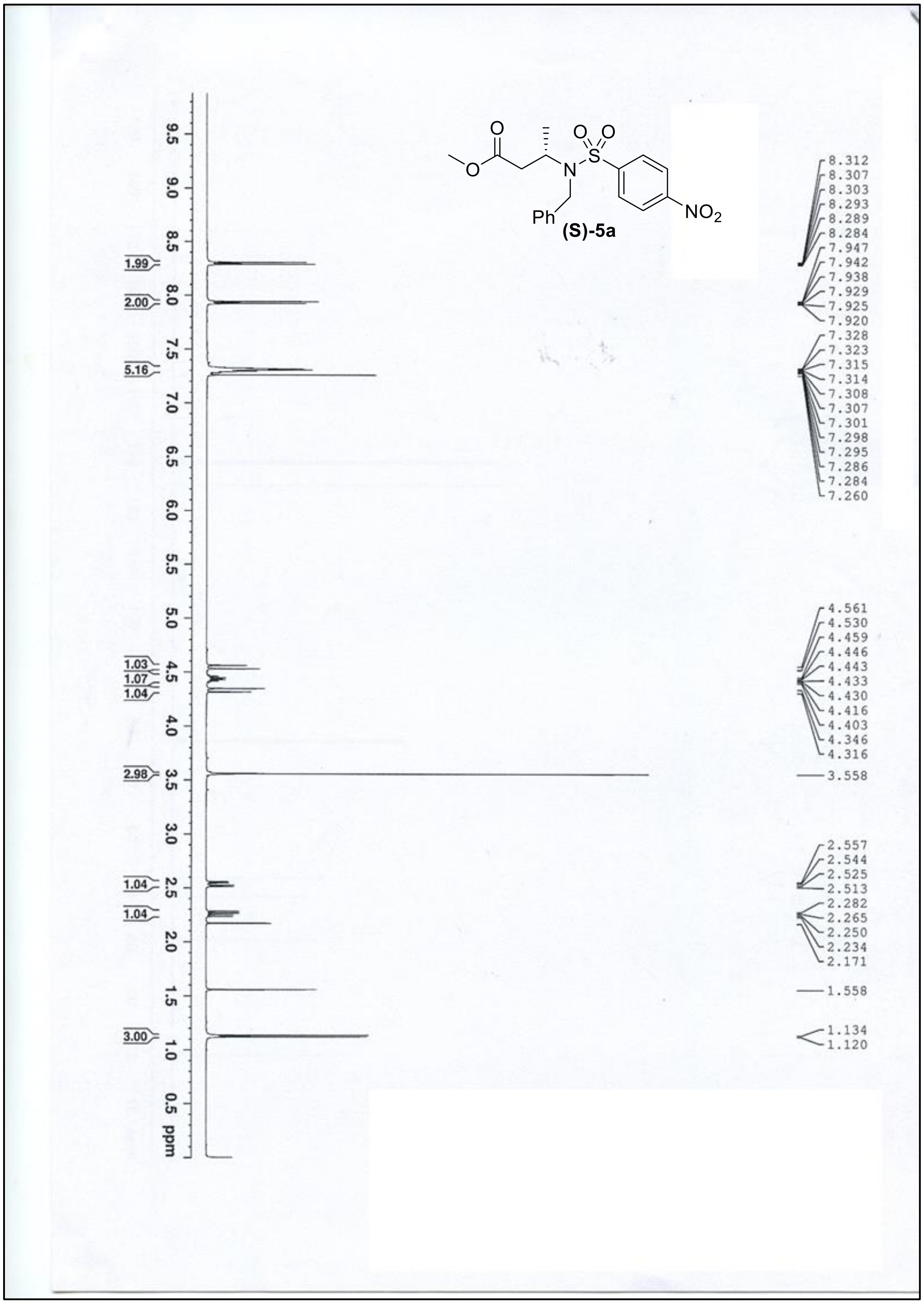

Figure S9, continued

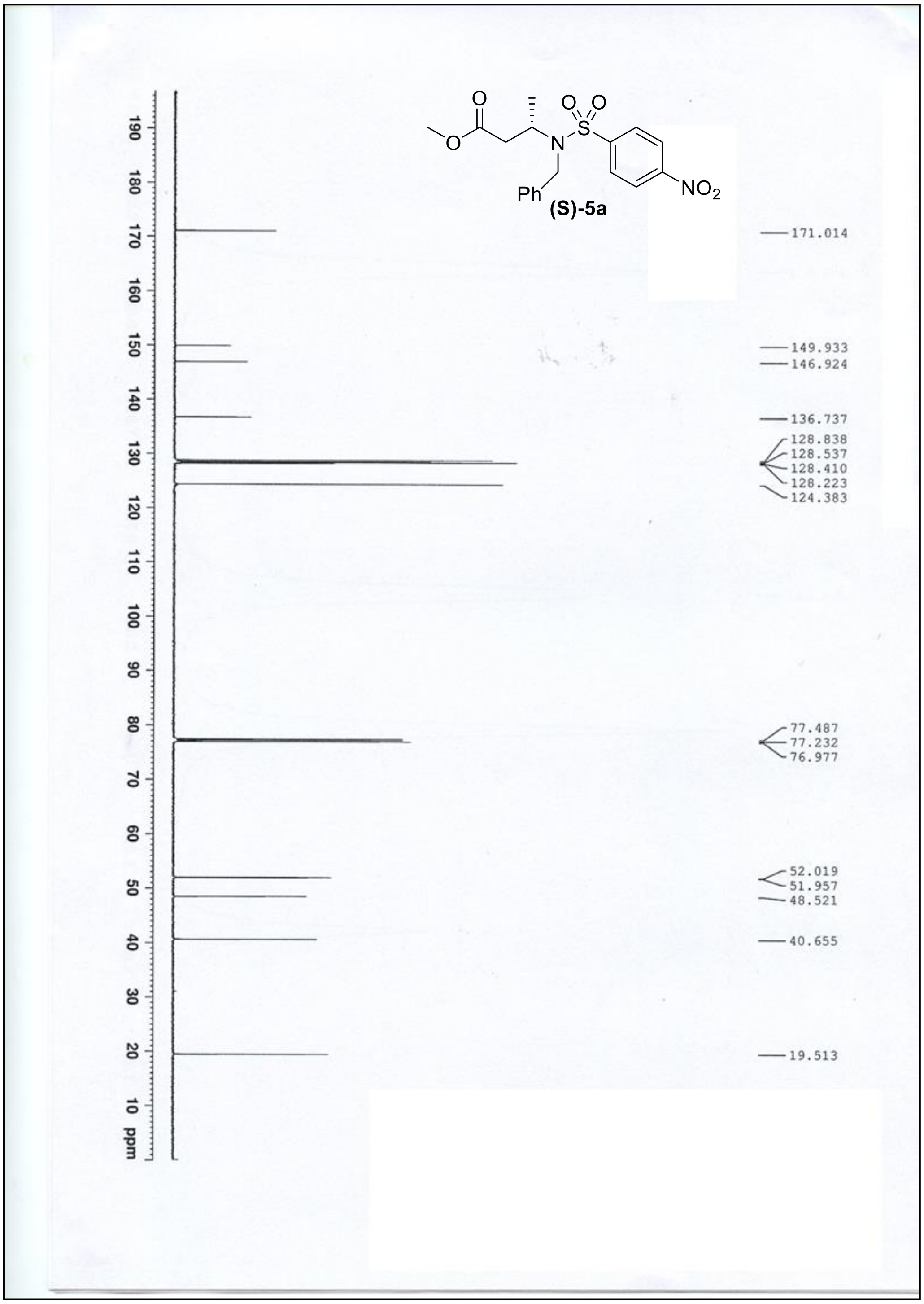

Figure S9, continued

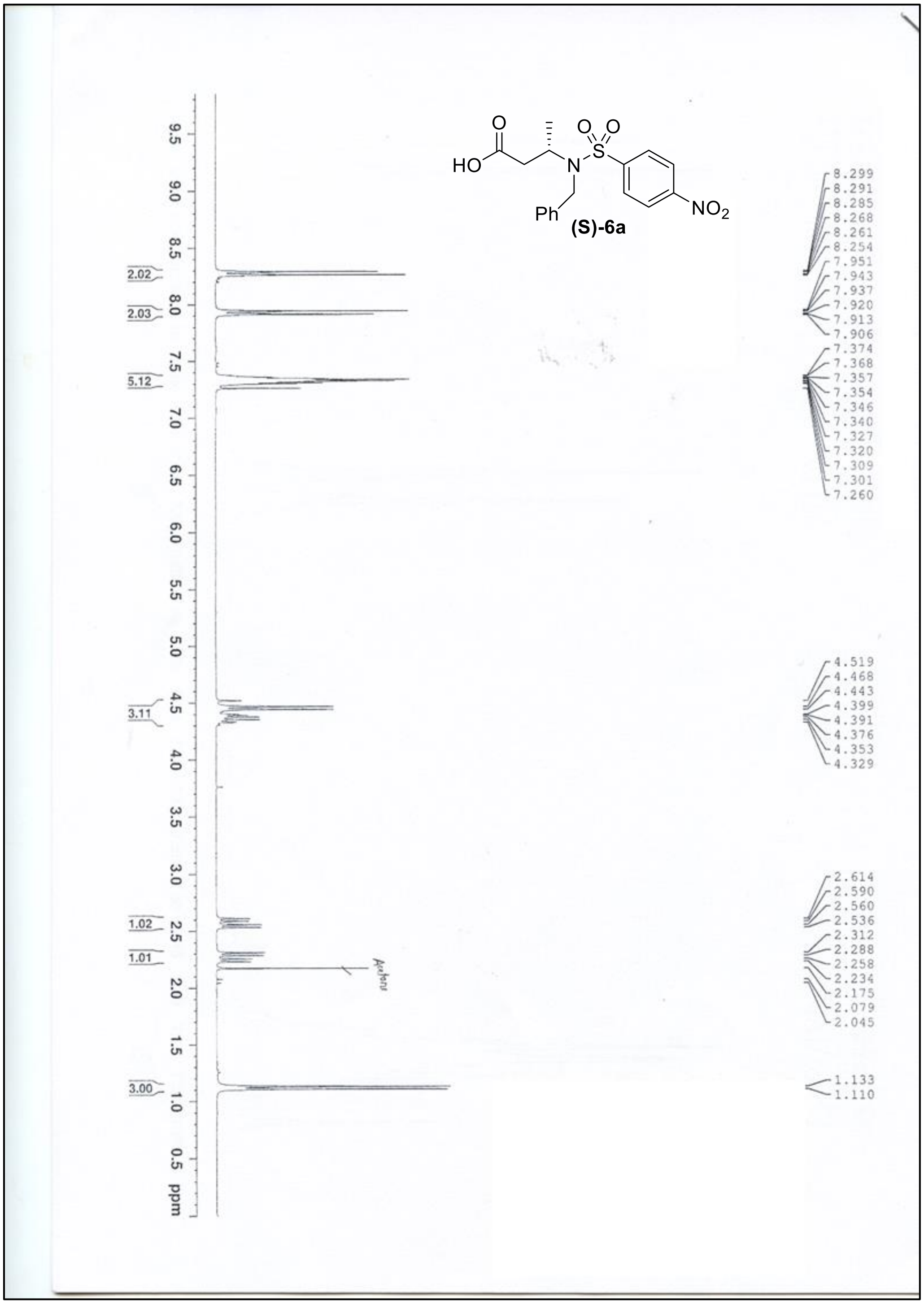

Figure S9, continued

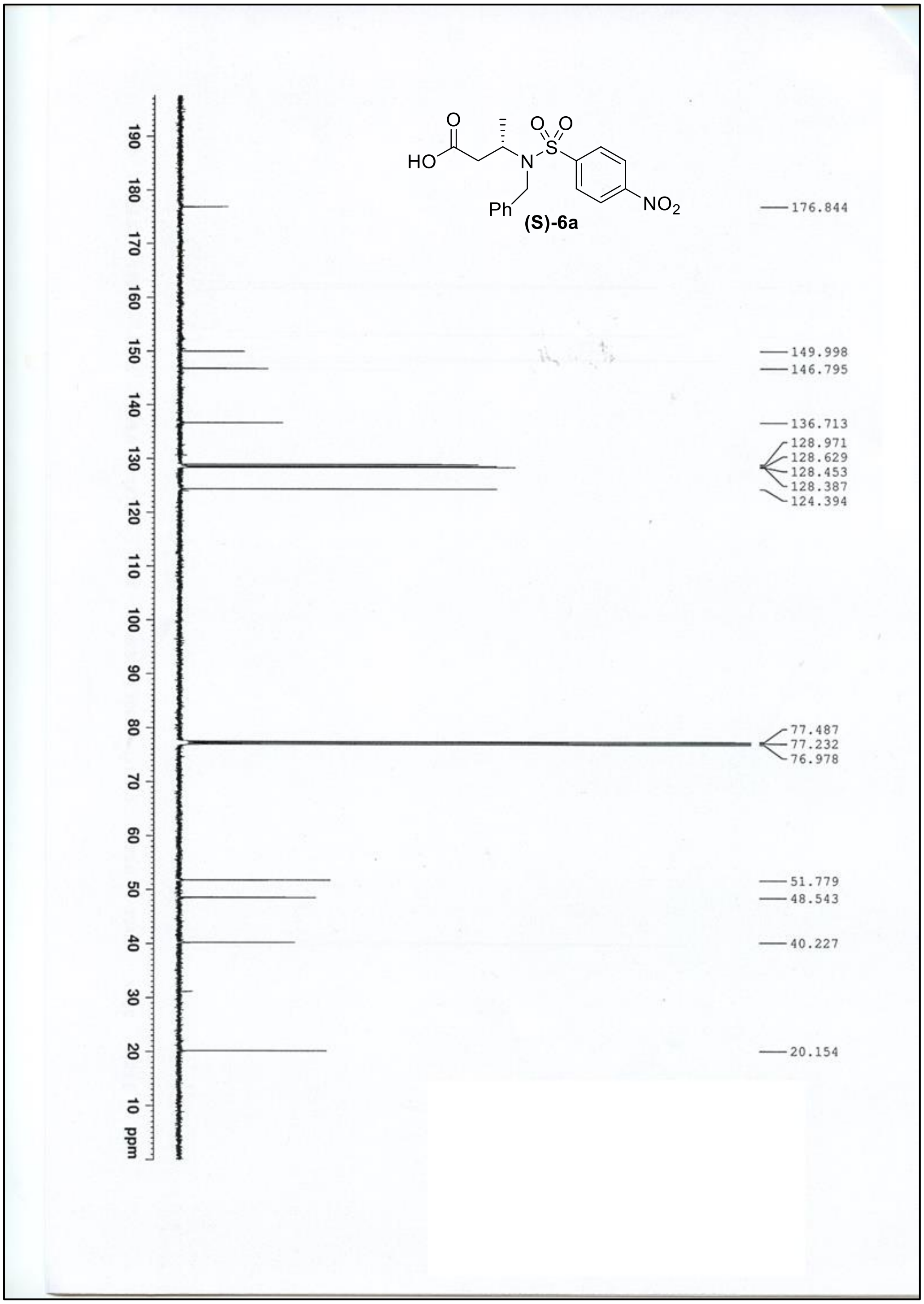

Figure S9, continued

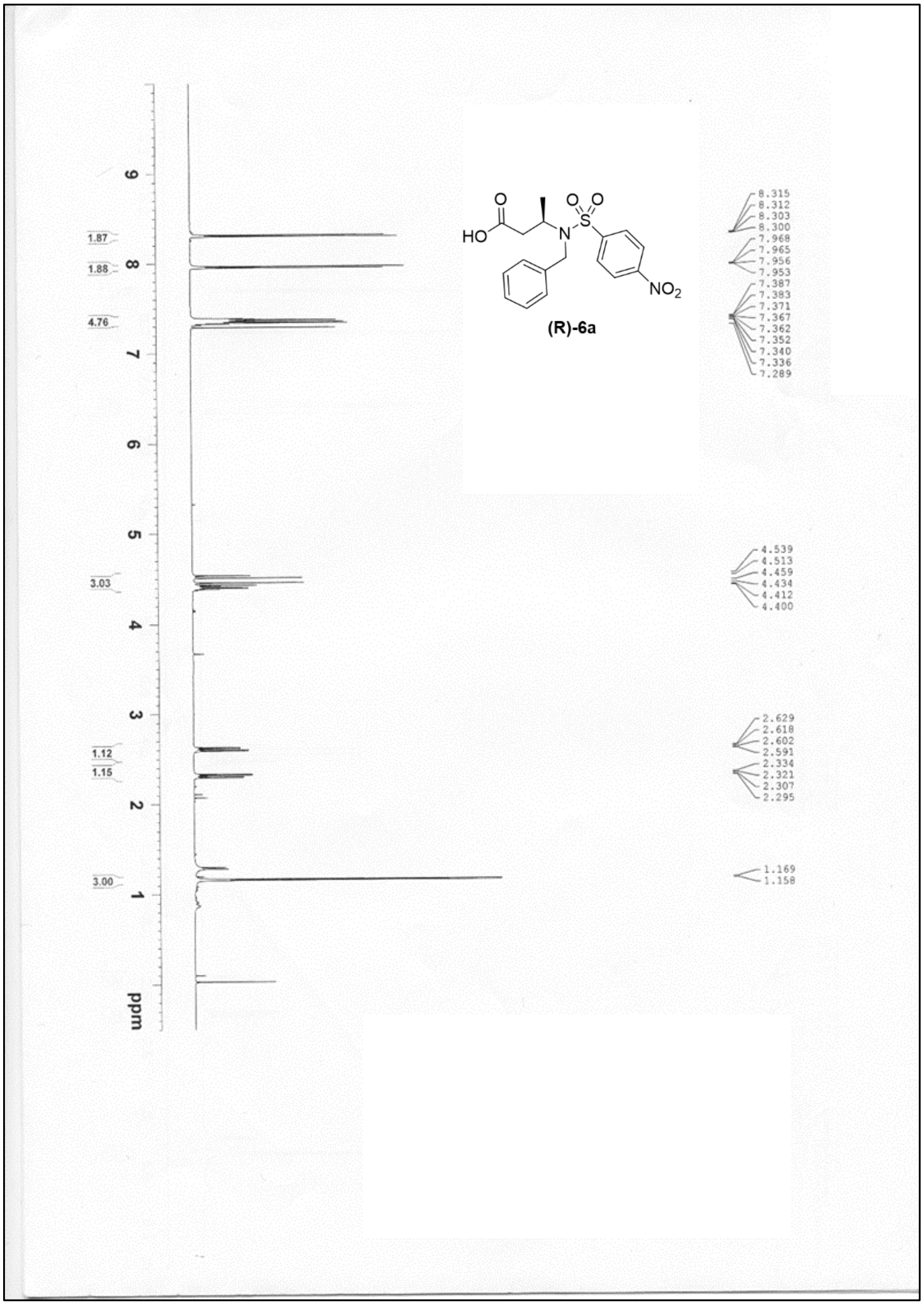

Figure S9, continued

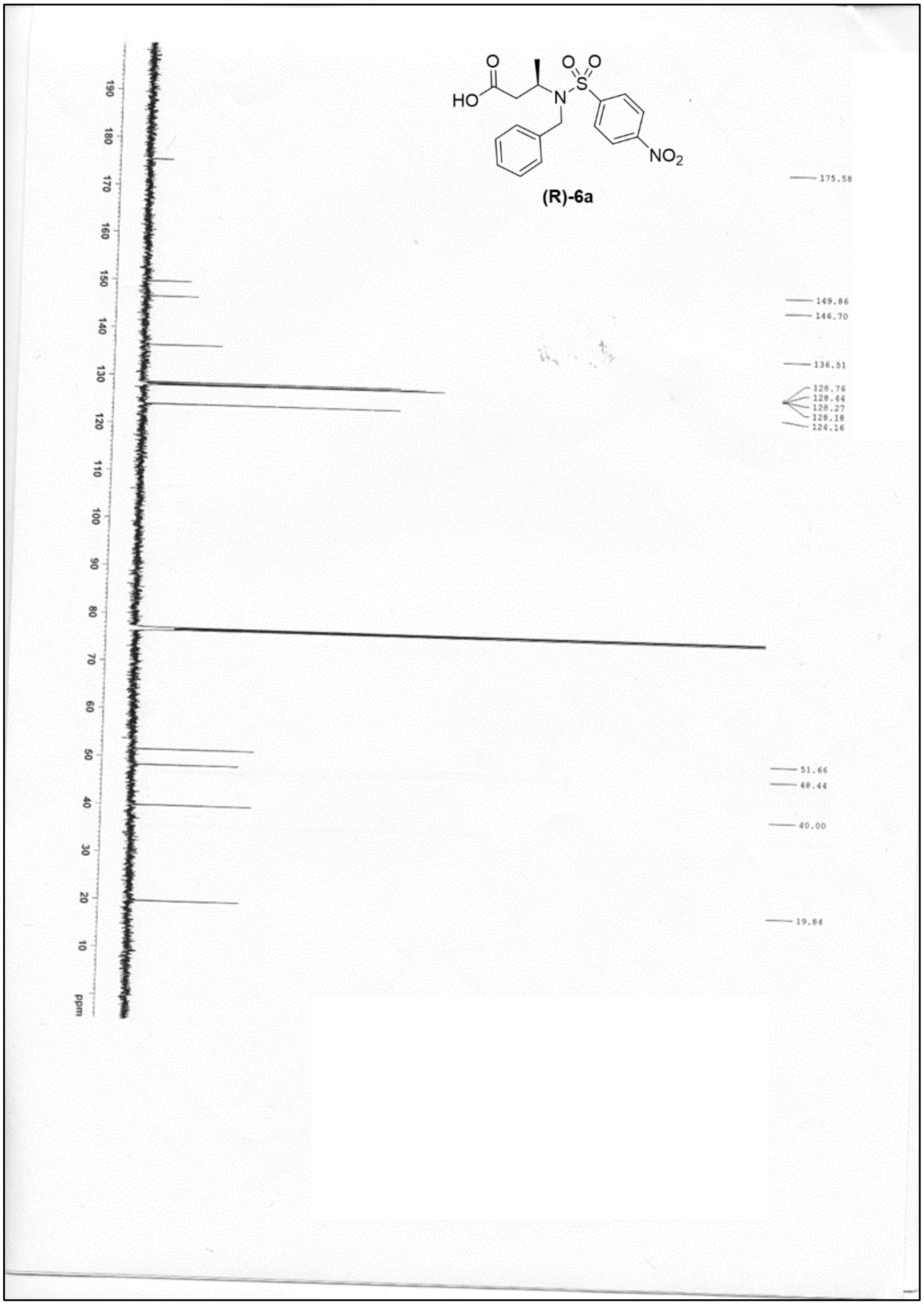

Figure S9, continued

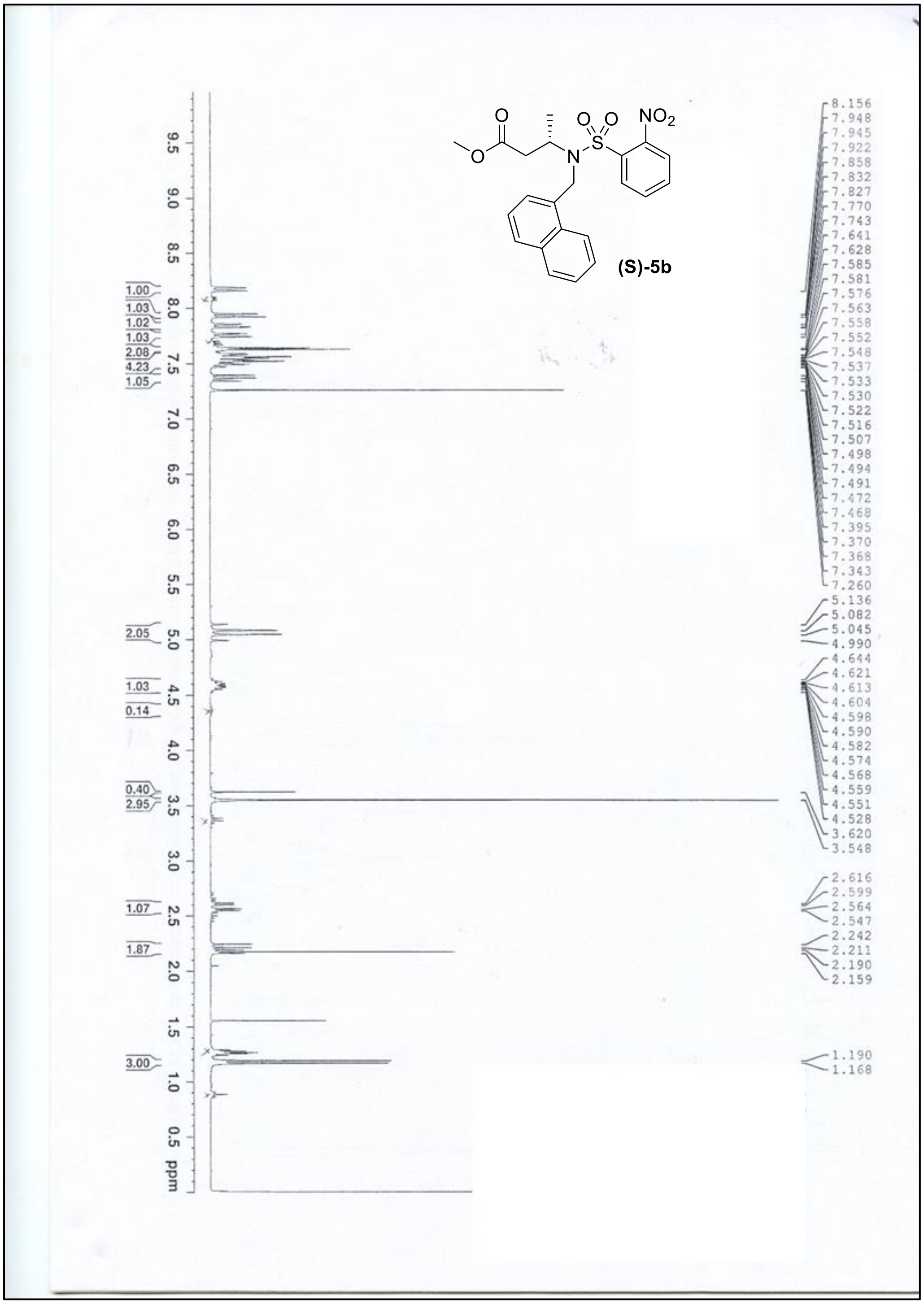

Figure S9, continued

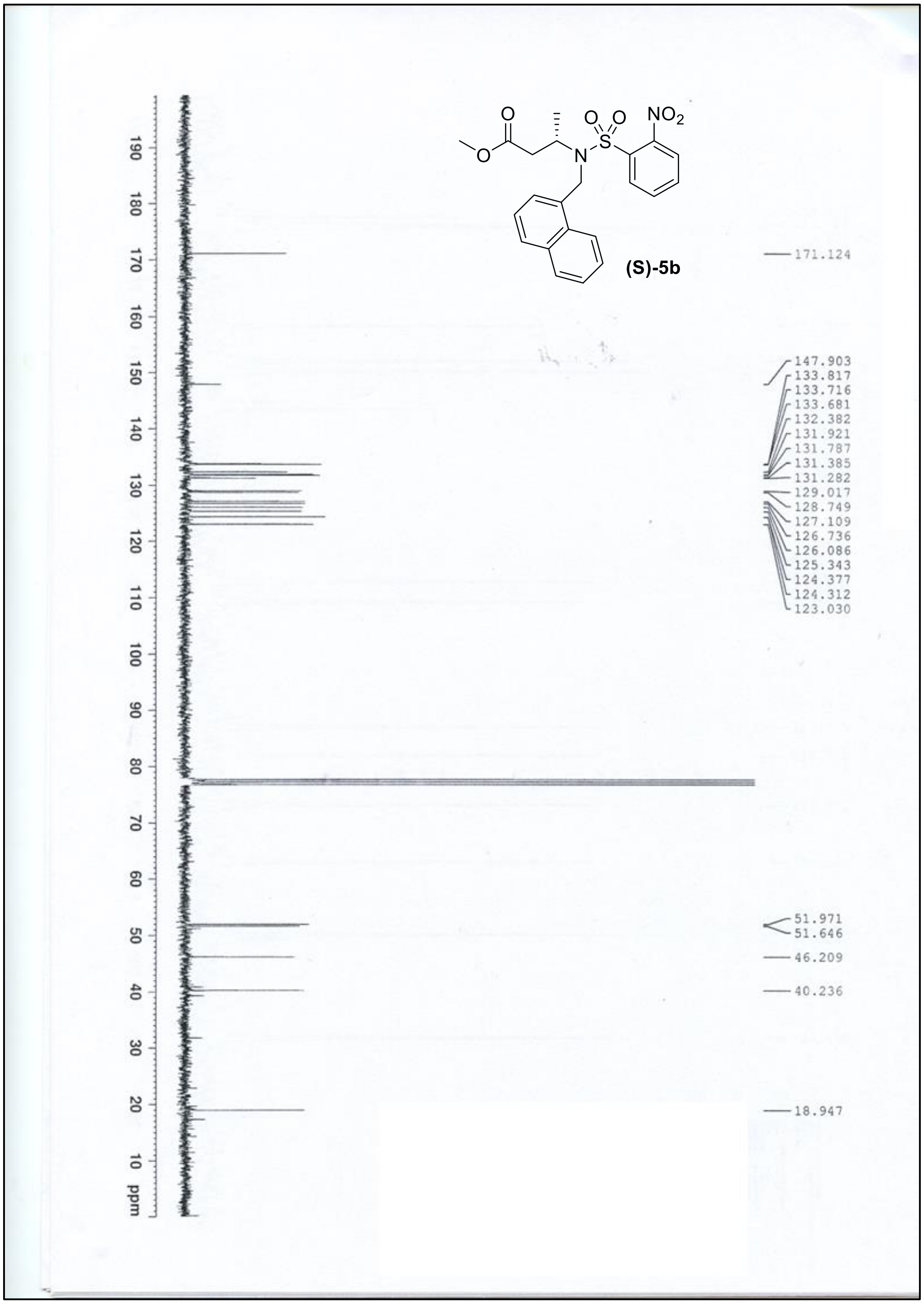

Figure S9, continued

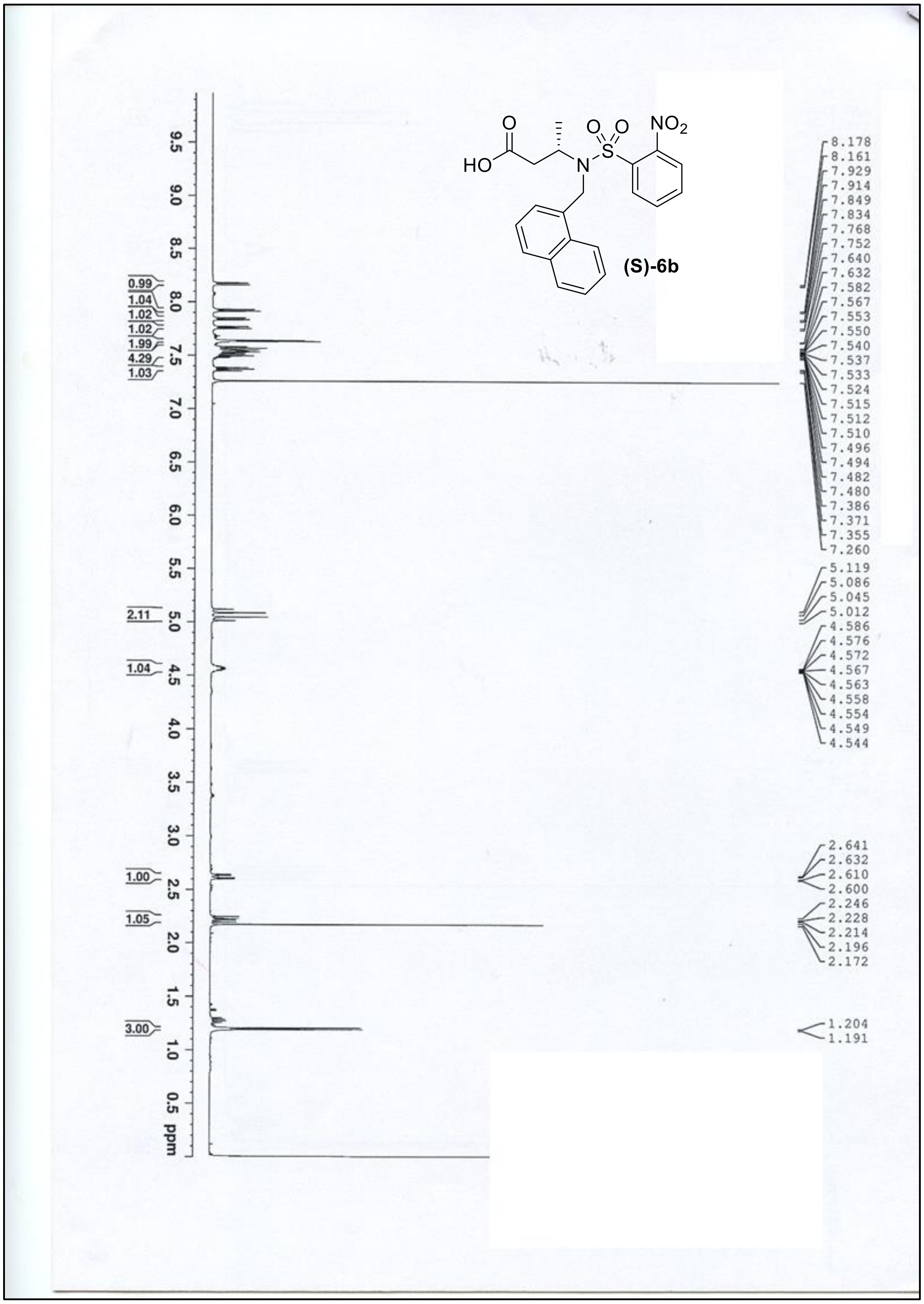

Figure S9, continued

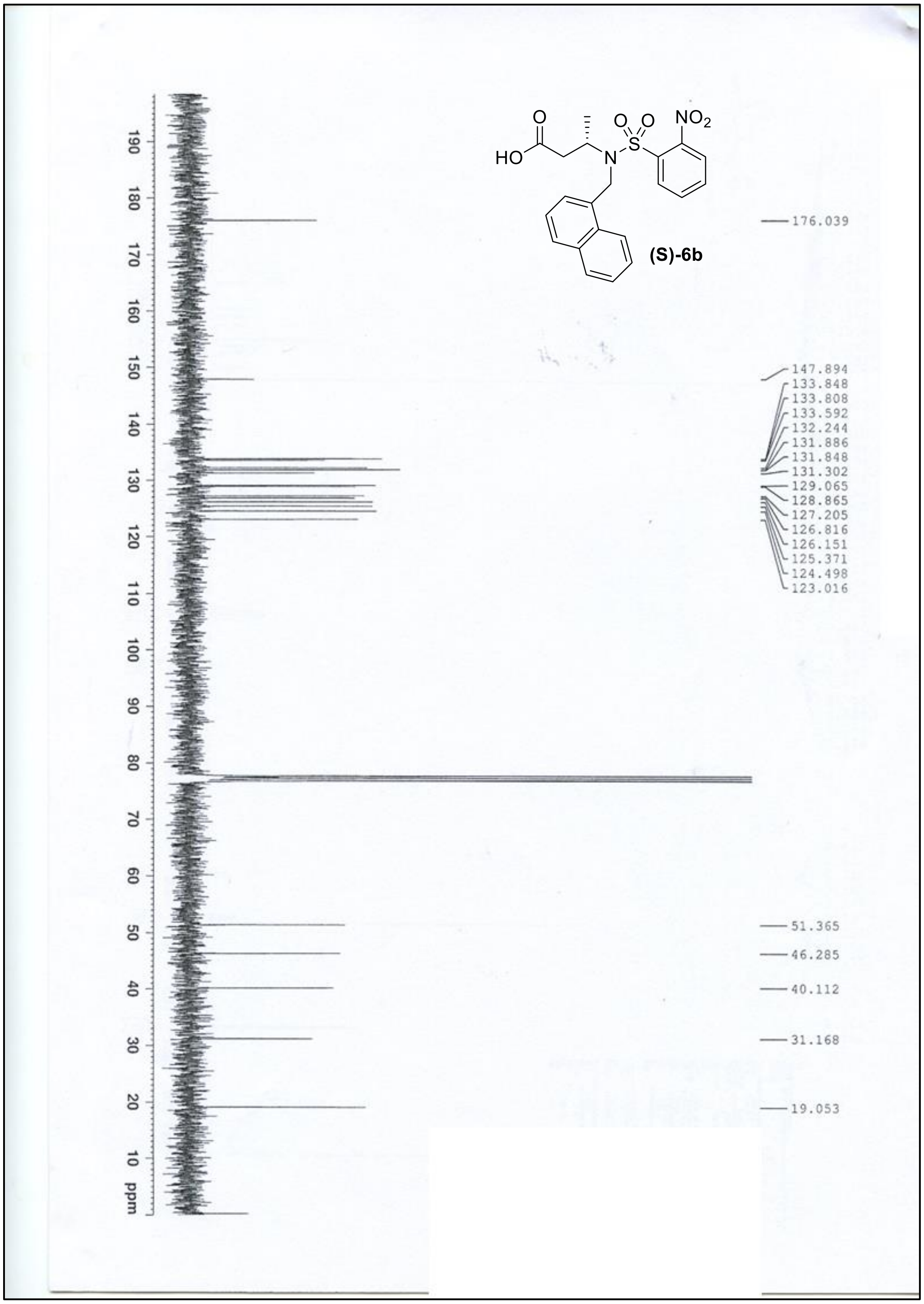

Figure S9, continued

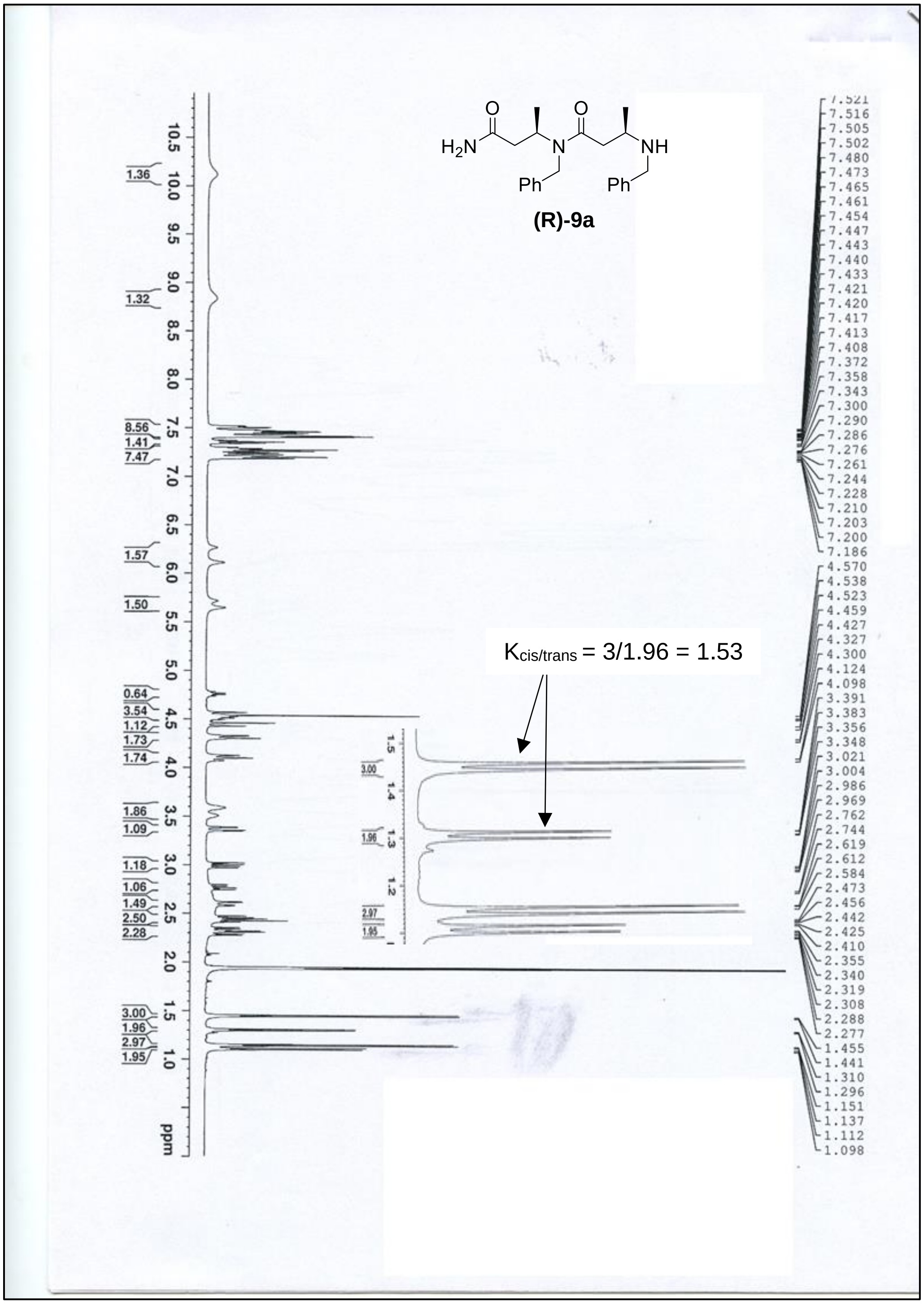

Figure S9, continued

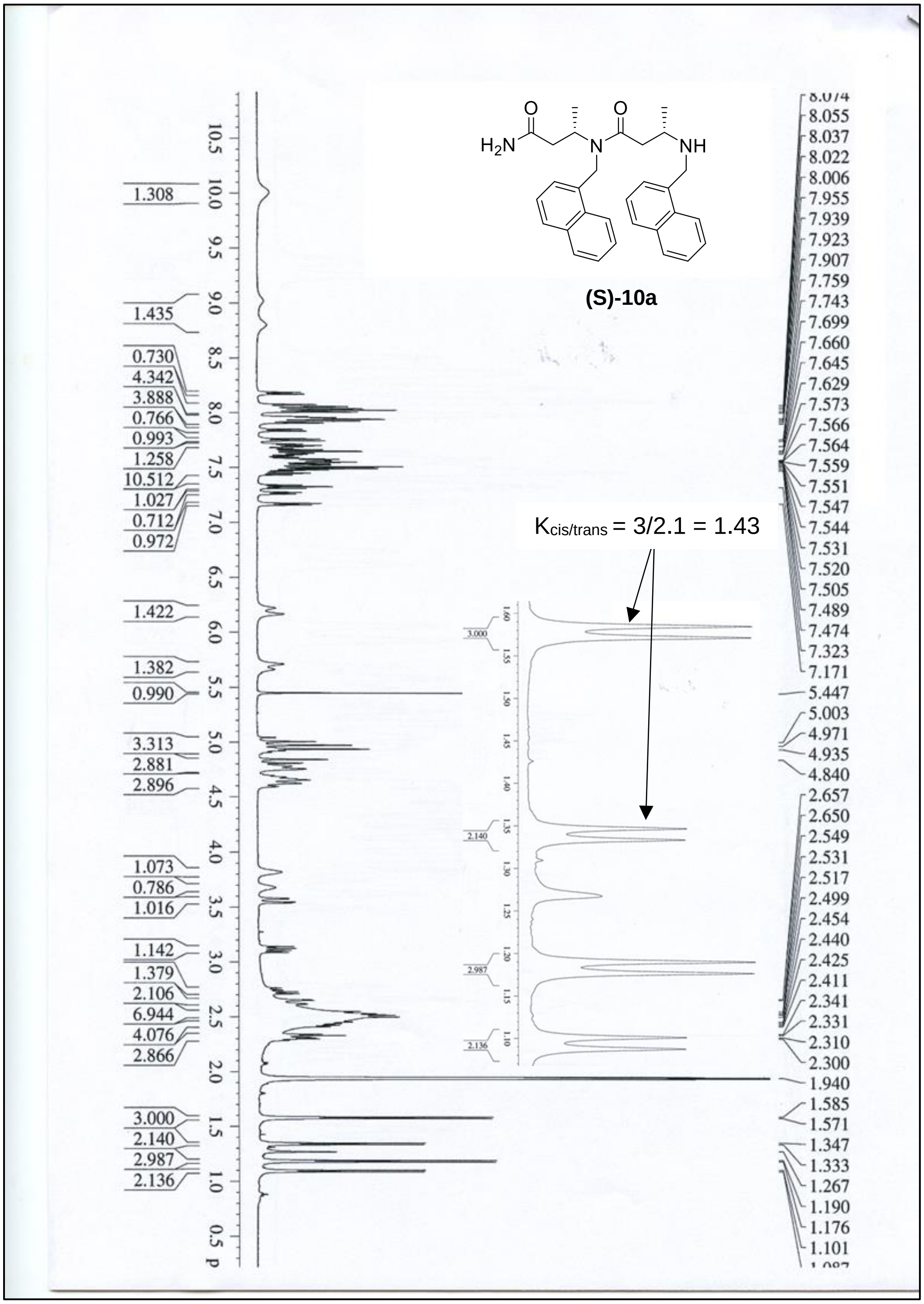

Figure S9, continued

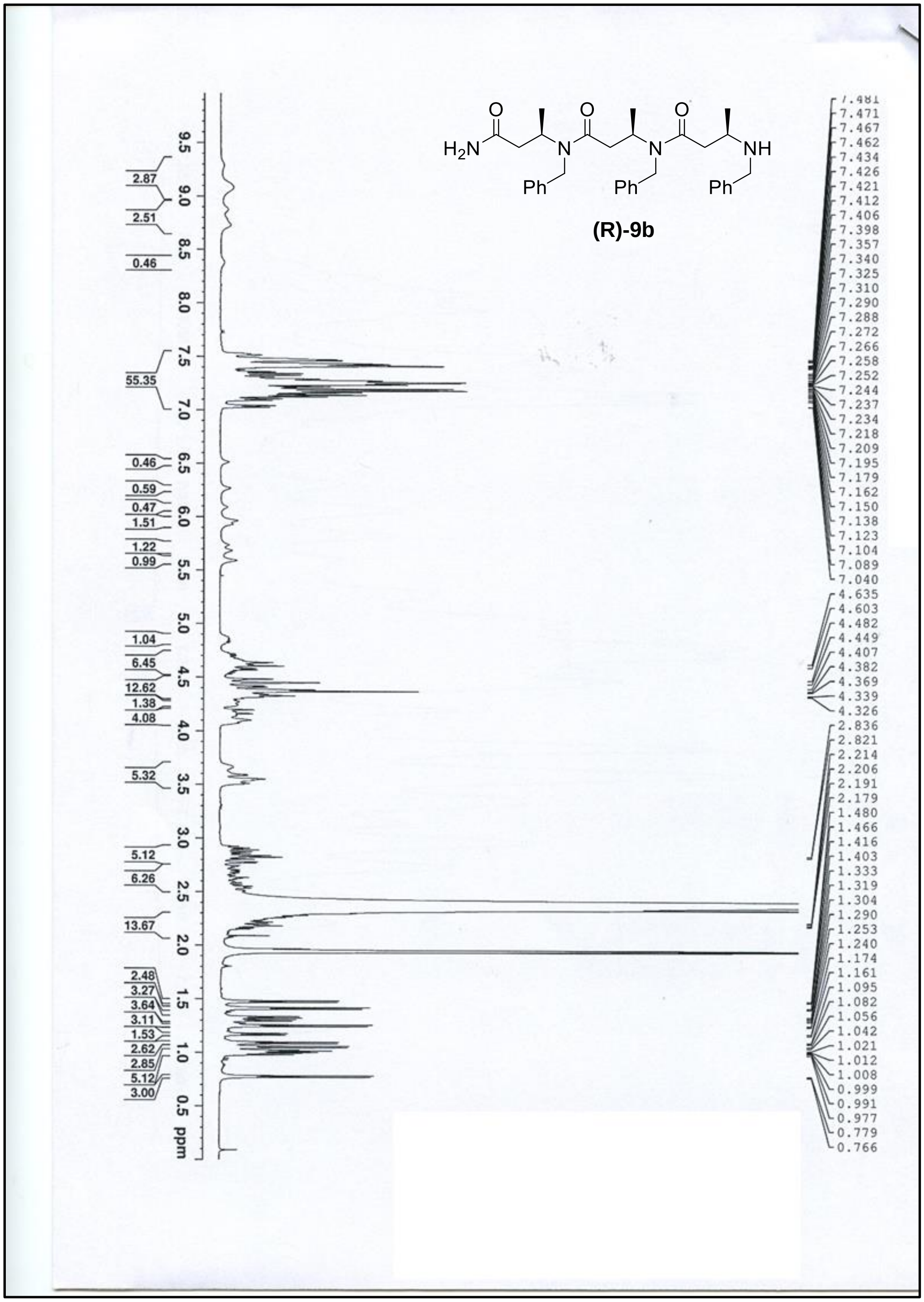

Figure S9, continued

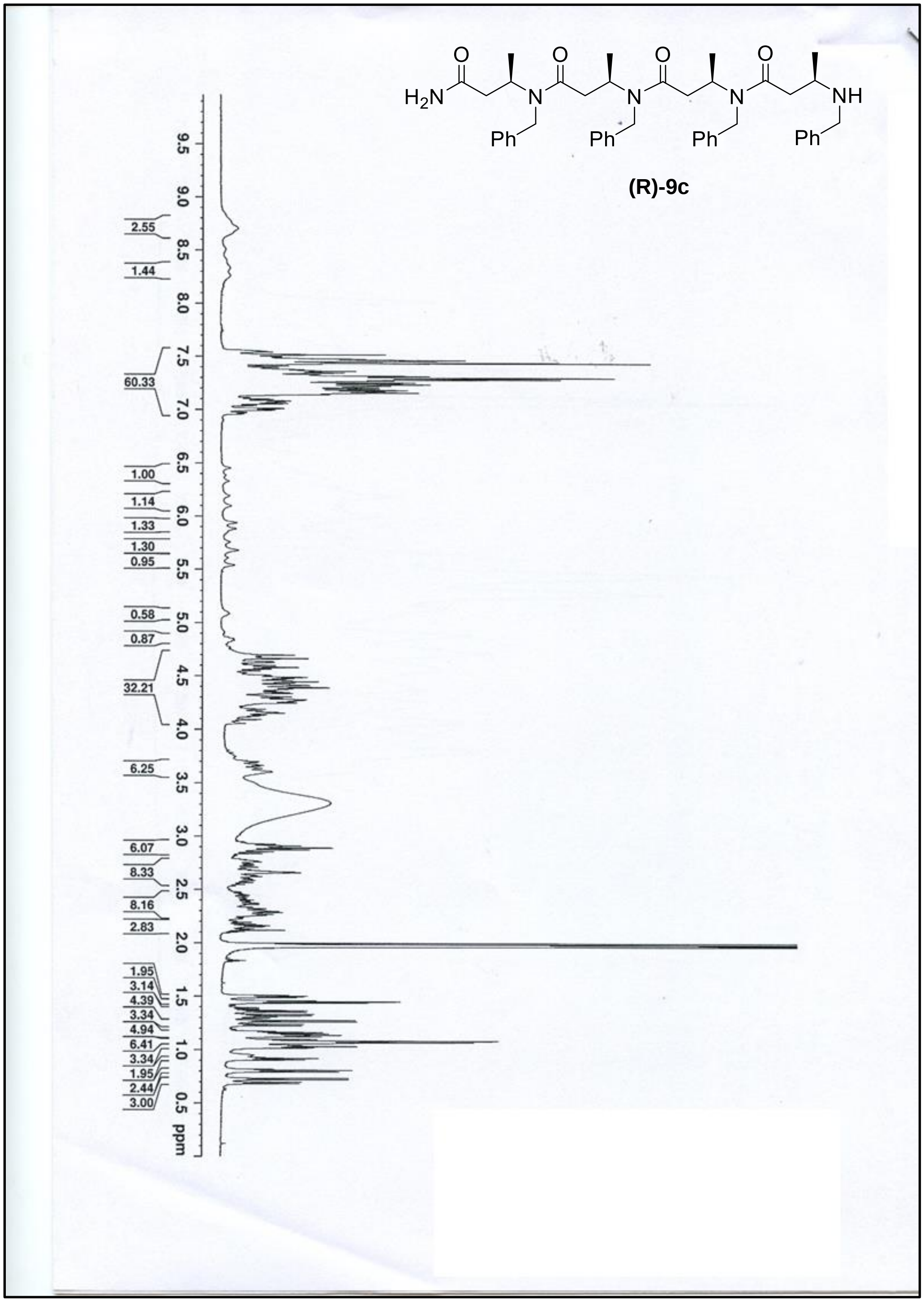

Figure S10. 2D ^{1}H NOESY spectra of (a) 2mer **(R)-9a** (b) 3mer **(R)-9b** and, (c) 4mer **(R)-9c**. [Dotted square regions indicate increase in additional NOEs from 2mer to 4mer, presumably due to the conformationally (cis) dominant ordered arrangement across the amide bond.]

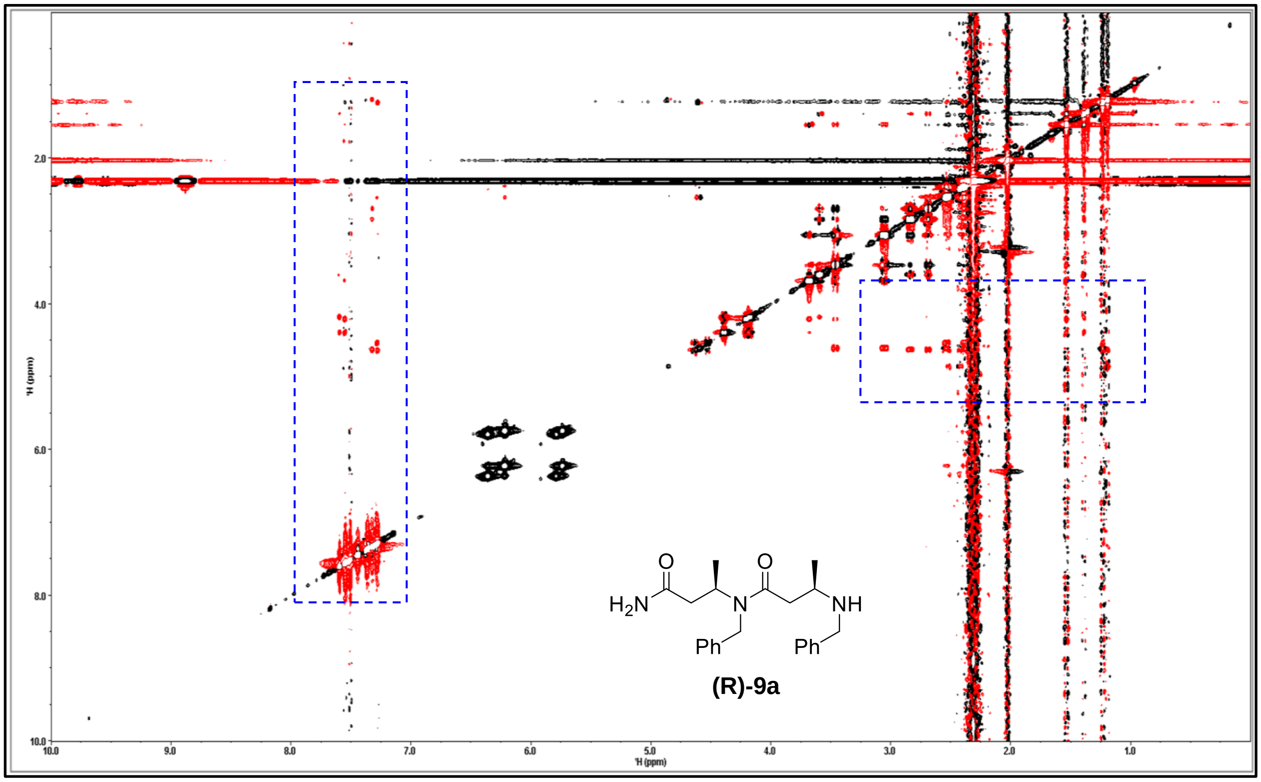

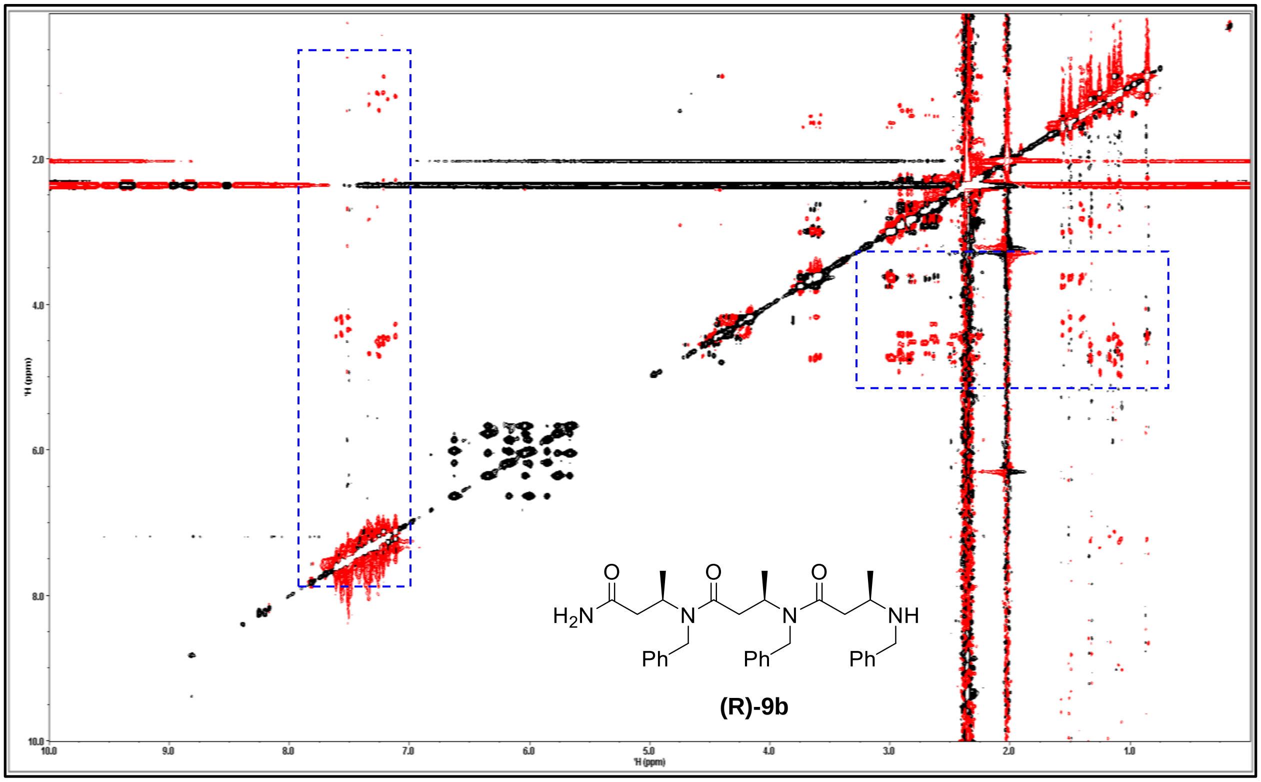

Figure S10, continued

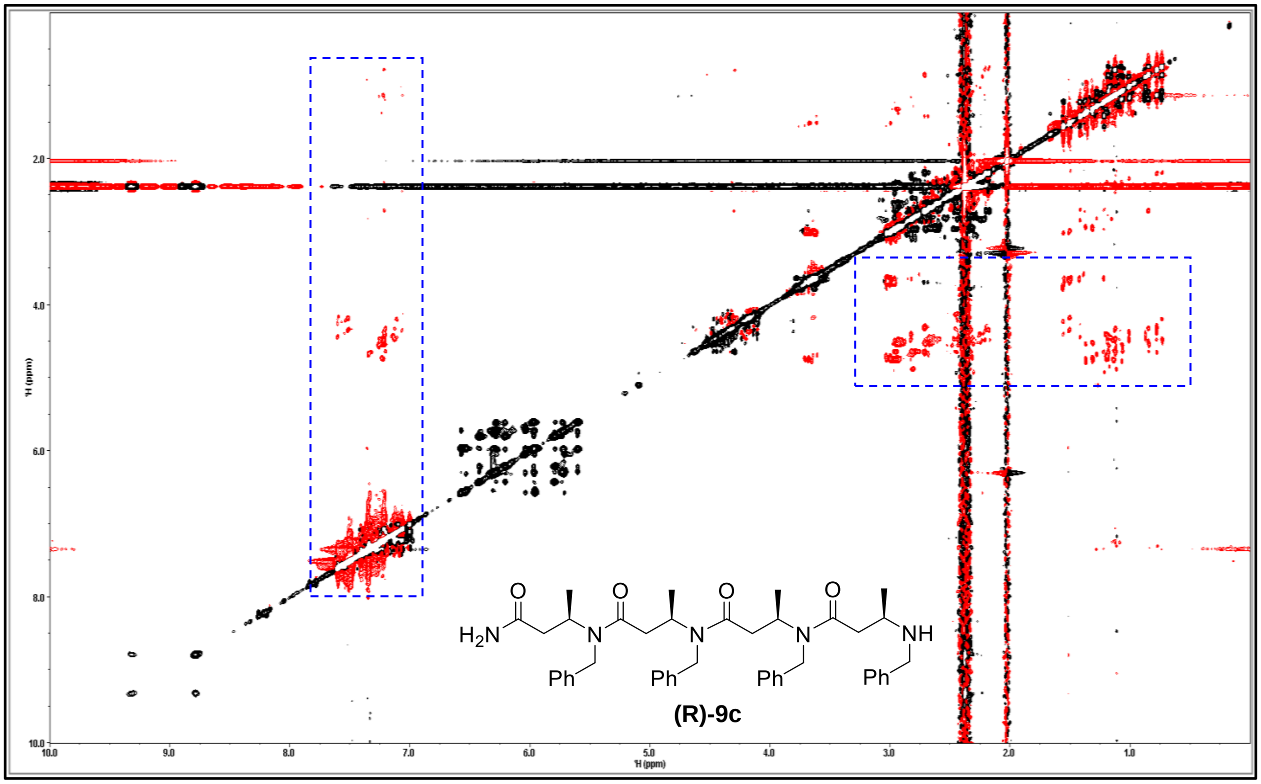

Supplement: Supplementary file 1 [file molecules-24-00178-s001.pdf]
